# *Ganoderma* (Ganodermataceae, Basidiomycota) Species from the Greater Mekong Subregion

**DOI:** 10.3390/jof7100819

**Published:** 2021-09-30

**Authors:** Thatsanee Luangharn, Samantha C. Karunarathna, Arun Kumar Dutta, Soumitra Paloi, Itthayakorn Promputtha, Kevin D. Hyde, Jianchu Xu, Peter E. Mortimer

**Affiliations:** 1 Centre for Mountain Futures (CMF), Kunming Institute of Botany, Kunming 650201, China; l.thatsanee1990@gmail.com (T.L.); samanthakarunarathna@gmail.com (S.C.K.); kdhyde3@gmail.com (K.D.H.); J.C.Xu@cgiar.org (J.X.); 2 CIFOR-ICRAF, World Agroforestry Centre (ICRAF), Kunming 650201, China; 3 Center of Excellence in Fungal Research, Mae Fah Luang University, Chiang Rai 57100, Thailand; 4 Department of Botany, West Bengal State University, Barasat 700126, India; arun.botany@gmail.com; 5 National Center for Genetic Engineering and Biotechnology (BIOTEC), National Science and Technology Development Agency (NSTDA), 113 Thailand Science Park, Phahonuyothin Rd., Khlong Nueng, Khlong Luang, Pathum Thani 12120, Thailand; soumitrabotany@gmail.com; 6 Department of Biology, Faculty of Science, Chiang Mai University, Chiang Mai 50200, Thailand; itthayakorn.p@cmu.ac.th; 7 Research Center in Bioresources for Agriculture, Industry and Medicine, Chiang Mai University, Chiang Mai 50200, Thailand; 8 Institute of Plant Health, Zhongkai University of Agriculture and Engineering, Haizhu District, Guangzhou 510225, China

**Keywords:** two new species, biogeography, ecological aspects, Lingzhi, medicinal mushroom, morphology

## Abstract

The cosmopolitan fungal genus *Ganoderma* is an important pathogen on arboreal plant hosts, particularly in tropical and temperate regions. It has long been used as a traditional medicine because of its medicinal properties and chemical constituents. In this study, *Ganoderma* collections were made in the Greater Mekong Subregion (GMS), encompassing tropical parts of Laos, Myanmar, Thailand, Vietnam, and temperate areas in Yunnan Province, China. The specimens used in this study are described based on micro-macro-characteristics and phylogenetic analysis of combined ITS, LSU, TEF1α, and RPB2 sequence data. In this comprehensive study, we report 22 *Ganoderma* species from the GMS, namely, *G. adspersum*, *G. applanatum*, *G. australe*, *G. calidophilum*, *G. ellipsoideum*, *G. flexipes*, *G. gibbosum*, *G. heohnelianum*, *G. hochiminhense*, *G. leucocontextum*, *G. lucidum*, *G. multiplicatum*, *G. multipileum*, *G. myanmarense*, *G. orbiforme*, *G. philippii*, *G. resinaceum*, *G. sichuanense*, *G. sinense*, *G. subresinosum*, *G. williamsianum*, and *G. tsugae*. Some of these species were reported in more than one country within the GMS. Of these 22 species, 12 were collected from Yunnan Province, China; three were collected from Laos; three species, two new records, and one new species were collected from Myanmar; 15 species and four new records were collected from Thailand, and one new species was collected from Vietnam. Comprehensive descriptions, color photographs of macro- and micro-characteristics, the distribution of *Ganoderma* within the GMS, as well as a phylogenetic tree showing the placement of all reported *Ganoderma* from the GMS are provided.

## 1. Introduction

*Ganoderma* was established by Karsten [1] with *Polyporus lucidus* (Curtis) Fr. (=*Ganoderma lucidum* (Curtis) P. Karst.) as the type species [2]. The genus has a worldwide distribution but is predominantly found in tropical and temperate regions, including Africa, America, Europe, and Asia [3,4,5,6]. Most members of *Ganoderma* are pathogenic in nature, inflicting various diseases to plants such as white rot and stem rot as well as wood decay [3,6,7,8,9]. The genus is circumscribed by sessile to stipitate basidiomata with double-walled basidiospores and interwall pillars [1,2]. Kirk et al. [10] mentioned that globally there are 80 species of *Ganoderma*, while two global fungal databases viz. Index Fungorum [11] and MycoBank [12] hold 459 and 503 records, respectively.

In light of an earlier taxonomic framework, *Ganoderma* included three subgenera viz. *Ganoderma* P. Karst., *Elfvingia* P. Karst., and *Trachyderma* Imazeki [13]. The subgenus *Ganoderma* includes two sections. *Ganoderma* and *Phaenema* contain laccate species (a cutis surface consisting of a palisade of inflated hyphal ends) with a shiny upper surface, whereas all non-laccate species (palisade is absent) with a dull upper surface are under the subgenus *Elfvingia* [14,15]. The subgenus *Trachyderma* has been deemed illegitimate and is treated as a synonym of *Ganoderma* due to the presence of the lichenized genus *Trachyderma* [15].

*Ganoderma* mushrooms have long been used as traditional medicine in many Asian countries [4]. In the Pharmacopoeia of the People’s Republic of China, *Ganoderma* species are recorded as being used over two millennia [16]. Traditional Chinese books proposed and classified *Ganoderma* species based on basidiocarp coloration [17]. *Ganoderma* species are suitable sources of natural bioactive compounds of high and low molecular weight, especially polysaccharides, protein, sterols, and triterpenoids [18,19,20]. These compounds are known to possess extensive therapeutic properties, such as antibacterial, antifungal, antiviral, anticancer, antitumor, anti-inflammatory, anti-hypotensive, and antioxidative agents [15,21,22,23,24,25]. Moreover, these compounds also treat many immunological diseases [15,22], making *Ganoderma* species a popular functional food [26].

The Greater Mekong Subregion (GMS) consists of Cambodia, Lao People’s Democratic Republic, Myanmar, Yunnan Province of the People’s Republic of China, Thailand, and Vietnam. The GMS is a biodiversity hotspot with rich fungal diversity [27], as well as featuring a high diversity of endemic and endangered organisms. A variable climate, vegetation type, and habitat primarily characterize the GMS, supporting the existence of thousands of macrofungal species, many of which remain unknown [28,29,30,31]. Wild edible and medicinal macrofungi comprise an important source of income for people residing in the GMS. Past work has shown that the GMS is a hotpot of *Ganoderma* diversity, and thus of interest to the broader community of scientists working on this genus, yet no studies have attempted to bring all this information into one space and update the knowledge the known distribution of species, as well as introduce newly discovered species. Thus, our study seeks to clarify the status of *Ganoderma* in the GMS, where it is both an important medicinal and plant pathogenic mushroom.

There is much debate among taxonomists regarding how to best resolve nomenclatural confusion within *Ganoderma*. This stems from the high degree of phenotypic plasticity found in *Ganoderma* species [4,32,33,34,35,36]. *Ganoderma lucidum* was broadly defined from the original concept [34], with variations occurring in macro-characteristics of the basidiomes, resulting in many synonyms and general redundancy in the taxonomy of this species and genus [34,37].

### 1.1. Biogeography of Ganoderma

*Ganoderma lucidum* (Curtis) P. Karst., the type species of *Ganoderma*, was described based on a specimen collected from the U.K. Moncalvo et al. [38] concluded that *G*. *lucidum* was distributed across northern and southern Europe and likely extended into China. The name *Ganoderma lingzhi* (Lingzhi) has been adopted in place of *G. lucidum* for specimens native to East Asia [4]. Among *Ganoderma* species, several species similar to *G. lucidum* have also been described worldwide: *G. multipileum* [39], *G. Sichuanense* [4,16,40] from China; *G. resinaceum* from Europe [41]; and *G. oregonense*, *G. sessile*, *G. tsugae*, and *G. zonatum* from the U.S. [42,43]. These species are accepted as members of the *G. lucidum* complex.

**Figure 1:**
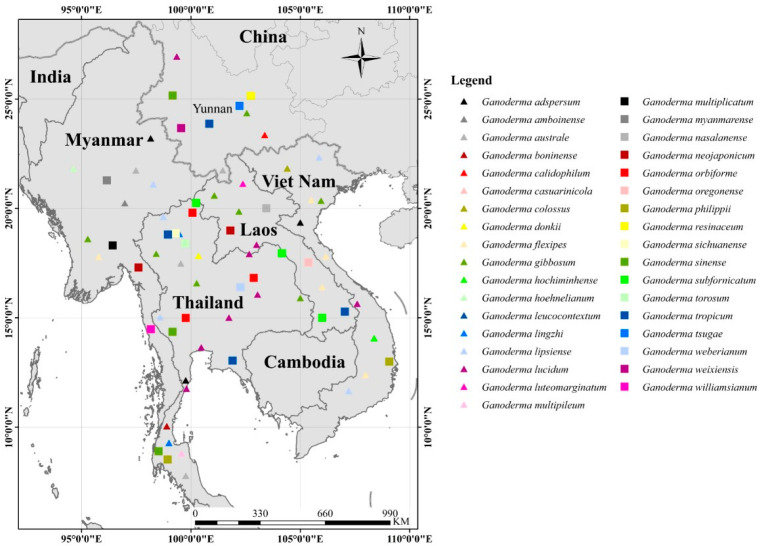
Distribution of *Ganoderma* species in the GMS.

**Figure 2:**
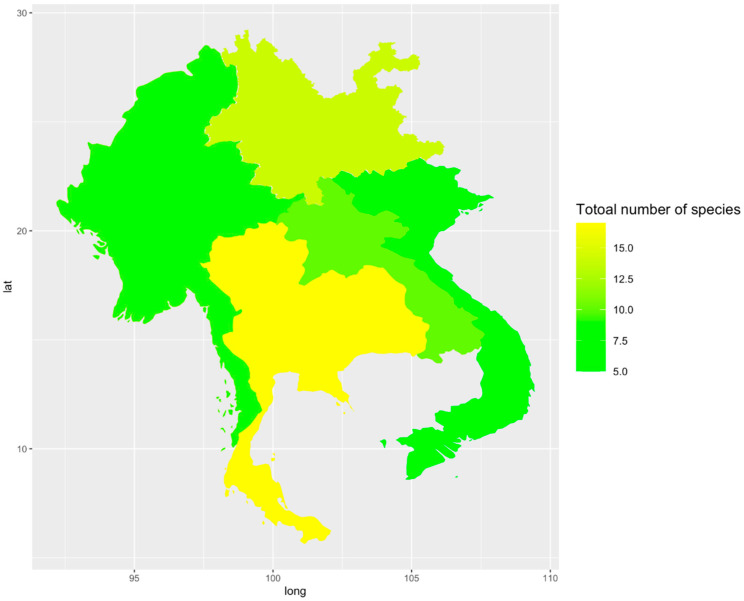
Species diversity (SD) hotspots of *Ganoderma* species in the GMS.

Taxonomic treatments of *Ganoderma* species remain unclarified due to the high degree of phenotypic plasticity [44,45]. Some *Ganoderma* species are distributed worldwide, such as *G. applanatum* and *G. australe*, while others are known only from specific localities [2,46]. Limited holotypes and unsettled geographical distributions have resulted in much ambiguity among *Ganoderma* taxa. *Ganoderma* has been reported from tropical Laos, Myanmar, Thailand, and Vietnam [30,31] and temperate China [6,31] in the GMS. Members of *Ganoderma* can be of significant importance in horticulture since they infect landscape plants, fruit trees, perennial crops, and economically important trees [27,29], which can be resulted in the death of affected trees. *Ganoderma* has been recorded mostly in a wide range of tree species, especially on hardwood trees [8]. *Ganoderma* has not been reported as host-specific. They are often found in natural forests (deciduous forests) and dominated on plantations trees [30,31]. Although *Ganoderma* has long been regarded as one of the genera with a worldwide distribution [30], the diversity and composition of *Ganoderma* in the GMS remains poorly understood. Here, we summarize and update the *Ganoderma* species distribution in the GMS based on location data from known collections (Figure 1). The extant *Ganoderma* species in the GMS are compiled in Table S1, and species diversity (SD) hotspots of *Ganoderma* in GMS are shown in Figure 2.

### 1.2. Ecological Aspects of Ganoderma

Wu and Dai [47] suggested that the morphology of *Ganoderma* species varies based on factors such as climate, nutrition, vegetation, and geography; moreover, morphology is not associated with the genetic material of a particular species. Basidiome features are influenced by the interaction of both intrinsic (genetic and physiological) and extrinsic (environmental) factors [48,49,50,51]. Therefore, comprehensive documentation of species ecology is necessary when studying *Ganoderma*. Criteria for identification vary: some authors strictly focus on geographical distribution, host-specificity, and macro-characteristics of basidiomes, while others focus on spore morphology as the primary taxonomic characteristic [52,53]. In the present study, ecological factors such as collection period, season, climate, host plant, and substrate of *Ganoderma* species were recorded.

*Ganoderma* species are known as both important wood-decaying fungi and pathogens that can survive on a wide range of hosts [54]. These fungi decay lignin, cellulose, and hemicellulose, resulting in a severe loss of woody plant strength [9,55]. They possess lignocellulose-decomposing enzymes useful for bioenergy production and bioremediation [56]. Diseases caused by *Ganoderma* species result in lower yields in economically important trees [54].

In this paper, we update biogeography records of *Ganoderma* by reporting species distributed across the Greater Mekong Subregion (GMS) as well as conducting morphological and phylogenetic studies on *Ganoderma* collected from temperate regions in Yunnan Province, China, and tropical regions of Laos, Myanmar, Thailand, and Vietnam (Table 1).

## 2. Materials and Methods

### 2.1. Study Sites

*Ganoderma* specimens were collected from the temperate parts of Yunnan Province, China, and tropical parts of Laos, Myanmar, Thailand, and Vietnam (Figure S1). Detailed collection site information, such as location, climate, the monthly temperature during the rainy season, host trees species, and native forest type, are provided in Table 2.

### 2.2. Sample Collection and Isolation

Fresh basidiomes of *Ganoderma* species were collected from China, Laos, Myanmar, Thailand, and Vietnam. The samples were photographed and transported back to the laboratory, where fresh macroscopic details were described. The cultures were aseptically isolated by using heat sterilized forceps, transferring sections of internal tissue from fruiting bodies onto potato dextrose agar (PDA) medium and incubated at 25–30 °C, for 1–3 weeks, under dark conditions [59]. After incubation, the agar surface was fully covered with white mycelium. The pure stock culture was then covered with mineral oil and deposited in the voucher culture collection of the Mae Fah Luang University culture collection (MFLUCC) Chiang Rai, Thailand. The cultures are being maintained at 4 °C for further studies. The voucher samples were then air-dried at 40 °C for 48 h until they were completely dehydrated. Finally, the material was deposited in the herbarium of Mae Fah Luang University (MFLU Herb.) with duplicates in the herbarium of Kunming Institute of Botany, Academia Sinica (HKAS), Yunnan Province, China.

### 2.3. Morphological Study

Macro-characteristics were described following the method of Lodge et al. [65], while color notations were recorded following Ridgeway [66]. Macroscopic characteristics were determined according to the methodology described by Largent [67]. To observe microscopic characteristics, free-hand sections were made under a dissecting microscope (OLYMPUS SZ61) and mounted on a glass slide containing 3–5% KOH, 1–3% Congo red, and Melzer’s reagent for highlighting all tissues [68]. Microphotography was performed with a Nikon ECLIPSE Ni (Nikon, Tokyo, Japan) compound microscope, with a Canon EOS 600D (Tokyo, Japan) digital camera fitted on the top of the microscope. Basidiospores and hyphal system sizes, colors, and shapes were recorded and photographed. Basidiospore measurements were taken using the Tarosoft^®^ Image Framework program v. 0.9.0.7. For measuring basidiospore statistics, the Tulloss’ standard format [69] was followed [Q = L/W], where Q, the quotient of basidiospore length to width (L/W) in side view and Qm, the mean of Q-values ± SD. The calculation was performed by measuring at least 50 basidiospores from each basidiomata [70]. Photographs were edited in Adobe Illustrator CS v. 3.0.

### 2.4. DNA Extraction, PCR Amplification, and Sequencing

Dried internal tissues of the basidiomes were grounded, and total DNA was extracted using the Biospin Fungus Genomic DNA Extraction Kit (BioFlux^®^). The ITS, LSU, RPB2, and TEF1α genes were amplified by polymerase chain reaction (PCR). The PCR amplifications were performed in a total volume of 25 μL of PCR mixtures containing 9.5 μL ddH_2_O, 12.5 μL of PCR master mix, 1 μL of DNA template, and 1 μL of each primer (10 μM). PCR amplification was carried out using primer pairs ITS5/ITS4 for internal transcribed spacer rDNA region (ITS1, 5.8S rDNA and ITS2), LROR/LR5 for the nuclear ribosomal large subunit 28S rDNA (LSU), fRPB2-5F/fRPB2-7cR for the partial RNA polymerase second largest subunit region (RPB2) [71,72,73], and EF1-983F/EF1-2218R for the partial translation elongation factor 1-alpha (TEF1α) [74]. The PCR cycling amplification conditions differed following amplified markers. For ITS and LSU, the following condition was used: 3 min at 94 °C, followed by 35 cycles of 95 °C for 30 s, 55 °C for 1 min, 72 °C for 1 min, followed by a final extension at 72 °C for 10 min. The amplification condition for TEF1α consisted of initial denaturation at 5.30 min at 95 °C, followed by 35 cycles of 94 °C for 1 min, 57 °C for 30 s and 72 °C for 1.30 min, followed by a final extension at 72 °C for 10 min. The cycling profile of 3 min at 94 °C followed by 35 cycles of 95 °C for 1 min, 52 °C for 2 min and 72 °C for 1 min, followed by a final extension at 72 °C for 10 min, was used for RPB2. The sequencing of PCR products was carried out by Sangon Biotech (Shanghai) Co., Ltd., Shanghai, China. The nuclear ribosomal internal transcribed spacer region (nrITS) of the fungi was amplified, and the sequence was deposited in GenBank to obtain the accession number.

### 2.5. Sequence Alignment and Phylogenetic Analyses

*Ganoderma* specimens’ sequences were subjected to standard BLAST searches of GenBank to determine the primary identity of the *Ganoderma* species. All the other sequences of taxa closely related to our *Ganoderma* species were retrieved from GenBank. Sequences with high similarity indices were determined from BLAST searches to find the closest matches with taxa and from recently published literature [31,37,75]. All sequences used to construct the phylogenetic tree are listed in Table 3. *Sanguinoderma rugosum* (Blume and T. Nees) Torrend (Cui 9011), and *Tomophagus colossus* (TC-02) were used as the outgroup taxa [62,76]. Sequences were aligned with MAFFT online server [77] and manually adjusted using Bioedit v. 7.2.5 [78] and Clustal X softwares [79]. Alignments were checked manually and optimized to allow maximum sequence similarity. Gaps were treated as missing data. Maximum parsimony (MP) analysis was performed using the PAUP beta 10 software version 4.0 [80].

Clades inferred from the MP analyses were further assessed by maximum likelihood (ML) bootstraps with 1000 replicates using random step-wise sequence additions, performed using RAxML-HPC2 on XSEDE v. 8.2.8 [81] on the CIPRES webportal [82], and carried out using raxmlGUI v. 1.3.1 [83]. The best-fitting substitution model for each single gene partition and the concatenated data set was determined in MrModeltest 2.3 [84]. Bayesian posterior probabilities (PP) with the GTR+I+G model were used for each partition. Bayesian Markov chain Monte Carlo (MCMC) analyses were conducted in MrBayes v. 3.2.2 [85]. The number of generations was set at 3,500,000, with trees being sampled every 100th generations (a total of 35,000 trees), resulting in an average standard deviation of split frequencies below 0.01. Based on the tracer analysis, the first sampled topologies of 8,750 trees representing 25% of total trees were discarded in the burn-in phase. The remaining 26,250 trees were used for calculating posterior probability (PP) values in the majority rule consensus tree.

Phylogenetic trees were visualized with FigTree v. 1.4.0 [86] and edited using Microsoft Office PowerPoint 2010 before being exported to Adobe Illustrator CS v. 3.0. Maximum likelihood (ML) and maximum parsimony (MP) bootstrap values equal to or greater than 70% and Bayesian posterior probabilities (BP) equal to or greater than 0.95 are presented above the branches.

**Table 3:** GenBank accession numbers for ITS, LSU, TEF1α, and RPB2 sequence data of the taxa used in this study and procured from GenBank based on the earlier studies for conducting phylogenetic analyses. Details of newly amplified sequences are represented in bold.

Fungal Species	Voucher	Locality	GenBank Accession no.				References
			ITS	LSU	RPB2	TEF1α	
*G* *. adspersum*	GACP15061220	Thailand	MK345425	–	MK371437	MK371431	[30]
***G*** ***. adspersum***	**MFLU 19-2177**	**Laos**	**MN396652**	–	**MN423113**	–	**This study**
***G*** ***. adspersum***	**MFLU 19-2178**	**Thailand**	**MN396653**	–	**MN423114**	**MN423149**	**This study**
***G*** ***. adspersum***	**MFLU 19-2220**	**Thailand**	**MN396655**	**MN428663**	**MN423116**	**MN423151**	**This study**
*G* *. angustisporum*	Cui 13817 (holotype)	Fujian, China	MG279170	–	MG367507	MG367563	[37]
*G* *. angustisporum*	Cui 14578	Guangdong, China	MG279171	–	–	MG367564	[37]
*G* *. applanatum*	FIN131R610	–	EF060004	–	–	–	GenBank
***G*** ***. applanatum***	**MFLU 19-2175**	**Thailand**	**MN396333**	–	–	–	**This study**
***G*** ***. applanatum***	**MFLU 19-2188**	**China**	**MN396332**	–	–	–	**This study**
*G* *. aridicola*	Dai 12588 (holotype)	Durban, South Africa	KU572491	–	–	KU572502	[87]
*G* *. australe*	GACP14081671	Hainan Island, China	MH106871	–	–	–	[88]
*G* *. australe*	GACP14061914	China	MK345428	–	–	MK371432	[30]
*G* *. australe*	MFLU 13-0534	Thailand	KP142173	–	–	MN423152	[59]
***G*** ***. australe***	**HKAS 97397**	**China**	**MN396656**	**MN428664**	–	–	**This study**
***G*** ***. australe***	**MFLU 19-2171**	**Lao**	**MN396657**	–	–	–	**This study**
*G* *. austroafricanum*	CBS138724	South Africa	KM507324	KM507325	–	–	[9]
*G* *. boninense*	WD 2028	Japan	KJ143905	KU220015	KJ143964	KJ143924	[76]
*G* *. boninense*	WD 2085	Japan	KJ143906	–	KJ143965	KJ143925	[76]
*G* *. boninense*	GbHap1	–	MK713555	–	–	–	GenBank
*G* *. boninense*	GB001	–	KX092000	–	–	–	GenBank
***G*** ***. calidophilum***	**MFLU 19-2174**	**Yunnan, China**	**MN398337**	–	–	–	**This study**
*G* *. carocalcareum*	DMC 322 (holotype)	Cameroon	EU089969	–	–	–	[46]
*G* *. carocalcareum*	DMC 513	Cameroon	EU089970	–	–	–	[46]
*G* *. casuarinicola*	Dai 16336 (holotype)	Guangdong, China	MG279173	–	MG367508	MG367565	[37]
*G* *. casuarinicola*	HKAS104639	Thailand	MK817650	MK817654	MK840868	MK871328	[31]
*G* *. chocoense*	QCAM3123	Ecuador	MH890527	–	–	–	[89]
*G* *. curtisii*	CBS 100132	USA	JQ781849	–	KJ143967	KJ143927	[76]
*G* *. destructans*	CMW43670	South Africa	KR183856	KR183860	–	–	[9]
*G* *. destructans*	CMW42146	South Africa	MG020245	–	–	MG020200	[9]
*G* *. ecuadorense*	ASL799	Ecuador	KU128524	KX228350	–	–	[90]
*G* *. ecuadorense*	PMC126	Ecuador	KU128525	KU128529	–	–	[90]
*G* *. ellipsoideum*	GACP14080966 (holotype)	Hainan, China	MH106867	–	–	–	[88]
*G* *. ellipsoideum*	GACP14080968	Hainan, China	MH106868	–	–	–	[88]
***G*** ***. ellipsoideum***	**MFLU 19-2221**	**Thailand**	**MN398339**	–	–	**MN423157**	**This study**
*G* *. enigmaticum*	Dai 15970	Africa	KU572486	–	MG367513	KU572496	[91]
*G* *. enigmaticum*	Dai 15971	Africa	KU572487	–	MG367514	KU572497	[91]
*G* *. flexipes*	Wei 5494	China	JN383979	–	–	–	[6]
***G*** ***. flexipes***	**MFLU 19-2198**	**Yunnan, China**	**MN398340**	**MN428665**	–	–	**This study**
*G* *. gibbosum*	SFC20150630- 23	Korea	KY364264	–	–	–	[92]
***G*** ***. gibbosum***	**HKAS 97411**	**Yunnan, China**	**MN398341**		–	–	**This study**
*G* ***. gibbosum***	**MFLU 19-2176**	**Thailand**	**MN396311**	–	**MN423118**	–	**This study**
***G*** ***. gibbosum***	**MFLU 19-2190**	**Laos**	**MN396310**	–	**MN423117**	–	**This study**
***G*** ***. hochiminhense***	**MFLU 19-2224** (holotype)	**Vietnam**	**MN398324**	**MN396390**	–	**MN423176**	**This study**
***G*** ***. hochiminhense***	**MFLU 19-2225**	**Vietnam**	**MN396662**	**MN396391**	–	**MN423177**	**This study**
*G* *. hoehnelianum*	Dai11995	Yunnan, China	KU219988	–	MG367497	MG367550	[91]
***G*** ***. hoehnelianum***	**MFLU 19-2168**	**Myanmar**	**MN396316**	–	**MN423123**	**MN423158**	**This study**
*G* *. leucocontextum*	Dai 15601	China	KU572485	–	MG367516	KU572495	[37]
***G*** ***. leucocontextum***	**HKAS 97401**	**Yunnan, China**	**MN396317**	**MN428670**	**MN423124**	–	**This study**
*G* *. lingzhi*	Wu 1006-38 (holotype)	Hubei, China	JQ781858	–	JX029980	JX029976	[4]
*G* *. lobatum*	JV1212/10J	USA	KF605676	–	–	KU572501	GenBank
*G* *. lucidum*	Rivoire 4195	France	KJ143909	–	KJ143969	–	[76]
*G* *. lucidum*	Cui 14404	Sichuan, China	MG279181	–	MG367519	MG367573	[37]
***G*** ***. lucidum***	**MFLU 19-2161**	**Yunnan, China**	**MN396338**	–	**MN423135**	**MN423168**	**This study**
***G*** ***. lucidum***	**MFLU 19-2162**	**Thailand**	**MN396341**	–	**MN423138**	–	**This study**
*G* *. martinicense*	LIP SWMart08-55 (holotype)	Martinica, France	KF963256	–	–	–	[93]
*G* *. mbrekobenum*	UMN7-3GHA (holotype)	Ghana	KX000896	KX000897	–	–	[90]
*G* *. mizoramense*	UMN-MZ4 (holotype)	India	KY643750	–	–	–	[94]
*G* *. multiplicatum*	Dai 13122	China	KU572488	–	–	KU572498	[95]
***G*** ***. multiplicatum***	**MFLU 19-2152**	**Yunnan, China**	**MN401405**	–	–	**MN423171**	**This study**
*G* *. multipileum*	CWN 04670	Taiwan PRC, China	KJ143913	–	KJ143972	KJ143931	[87]
***G*** ***. multipileum***	**MFLU 19-2166**	**Thailand**	**MN401406**	–	**MN423142**	**MN423172**	**This study**
*G* *. mutabile*	Yuan 2289	Yunnan, China	JN383977	–	–	–	[76]
***G*** ***. myanmarense***	**MFLU 19-2167** (holotype)	**Myanmar**	**MN396330**	**MN428672**	–	–	**This study**
***G*** ***. myanmarense***	**MFLU 19-2169**	**Myanmar**	–	**MN398325**	–	–	**This study**
*G* *. nasalaense*	GACP17060211 (holotype)	Laos	MK345441	MK346831	–	–	[30]
*G* *. neojaponicum*	ASI 7032	Korea	JQ520193	–	–	–	[96]
*G* *. orbiforme*	Cui 13918	Hainan, China	MG279186	–	MG367522	MG367576	[37]
*G* *. orbiforme*	GACP14061420	Laos	MK345447	MK346833	–	–	[30]
***G*** ***. orbiforme***	**MFLU 17-1933**	**Thailand**	**MN401408**	–	**MN423144**	–	**This study**
*G* *. oregonense*	CBS 265.88	USA	JQ781875	–	KJ143974	KJ143933	[76]
*G* *. philippii*	E7098	Malaysia	AJ536662	–	–	–	[97]
*G* *. philippii*	E7425	Malaysia	AJ608713	–	–	–	[97]
***G*** ***. philippii***	**MFLU 19-2222**	**Thailand**	**MN401410**	**MN398326**	–	**MN423174**	**This study**
***G*** ***. philippii***	**MFLU 19-2223**	**Thailand**	**MN401411**	**MN398327**	–	**MN423175**	**This study**
*G* *. podocarpense*	QCAM6422 (holotype)	Ecuador	MF796661	MF796660	–	–	[94]
*G* *. resinaceum*	HMAS86599	England	AY884177	–	JF915435	–	GenBank
*G* *. resinaceum*	CBS 194.76	Netherlands	KJ143916	–	–	KJ143934	[76]
***G*** ***. resinaceum***	**MFLU 19-2153**	**Yunnan, China**	**MN398315**	**MN398328**	–	–	**This study**
*G* *. ryvardenii*	HKAS 58053 (holotype)	Cameroon, Africa	HM138671	–	–	–	[98]
*G* *. ryvardenii*	HKAS 58054	Cameroon, Africa	HM138672	–	–	–	[98]
*G* *. sanduense*	GACP18012501 (holotype)	China	MK345450	–	–	–	[30]
*G* *. sanduense*	GACP18012502	China	MK345451	–	–	–	[30]
*G* *. sessile*	JV 1209/9	USA	KF605629	–	–	KJ143936	[76]
*G* *. sessile*	JV 1209/27	USA	KF605630	–	KJ143976	KJ143937	[76]
*G* *. shandongense*	Dai 15785	Shandong, China	MG279190	–	MG367526	MG367580	[37]
*G* *. shandongense*	Dai 15791	Shandong, China	MG279192	–	MG367528	MG367582	[37]
*G* *. sichuanense*	HMAS 42798 (holotype)	Sichuan, China	JQ781877	–	–	–	[4]
***G*** ***. sichuanense***	**MFLU 19-2164**	**Thailand**	**MN396324**	–	**MN423130**	**MN423163**	**This study**
***G*** ***. sichuanense***	**HKAS 97398**	**Yunnan, China**	**MN396319**	–	**MN423126**	**MN423159**	**This study**
*G* *. sichuanense*	CGMCC5.2175	Sichuan, China	KC662402	–	–	–	[99]
*G* *. sinense*	Wei 5327	Hainan, China	KF494998	KF495008	MG367529	KF494976	[37]
***G*** ***. sinense***	**MFLU 19-2172**	**Thailand**	**MN398319**	**MN398332**	**MN423146**	–	**This study**
***G*** ***. sinense***	**MLFU 19-2173**	**Yunnan, China**	**MN398316**	**MN398329**	–	–	**This study**
*G* *. steyaertianum*	MEL:2382783	Australia	KP012964	–	–	–	GenBank
*G* *. steyaertianum*	6 WN 20B	Indonesia	KJ654462	–	–	–	[100]
*G* *. subresinosum*	7-SU-3-C-70 (M)-B	Indonesia	KJ654472	–	–	–	GenBank
***G*** ***. subresinosum***	**MFLU 17-1912**	**Thailand**	**MN398321**	–	–	–	**This study**
*G* *. thailandicum*	HKAS104640 (holotype)	Thailand	MK848681	MK849879	MK875831	MK875829	[31]
*G* *. thailandicum*	HKAS104641	Thailand	MK848682	MK849880	MK875832	MK875830	[31]
*G* *. tropicum*	Yuan 3490	Yunnan, China	JQ781880	–	–	KJ143938	[4]
*G* *. tropicum*	Dai 16434	Hainan, China	MG279194	–	MG367532	MG367585	[37]
***G*** ***. tropicum***	**HKAS 97486**	**Thailand**	**MH823539**	**MH823540**	**MH883621**	–	**[75]**
*G* *. tsugae*	Dai12751b	USA	KJ143919	–	KJ143977	KJ143939	[76]
***G*** ***. tsugae***	**HKAS 97406**	**Yunnan, China**	**MG279195**	–	**MG367533**	**MG367586**	**This study**
*G* *. valesiacum*	CBS428.84	USA	JQ520218	–	–	–	[96]
*G* *. weberianum*	CBS219.36	Philippines	JQ520219	–	–	–	[96]
*G* *. weberianum*	GanoTK17	Cameroon	JN105705	–	–	–	GenBank
*G* *. wiiroense*	UMN-20-GHA (para type)	Ghana	KT952361	–	–	–	[101]
*G* *. williamsianum*	Dai 16809	Thailand	MG279183	–	MG367535	MG367588	[101]
***G*** ***. williamsianum***	**MFLU 19-2170**	**Myanmar**	**MN398323**	**MN398334**	–	–	**This study**
*G* *. wuzhishanense*	GACP14081689	Hainan, China	KU994772	–	–	–	[76,102]
*G* *. zonatum*	FL-02	USA	KJ143921	–	KJ143979	KJ143941	[76]
*Sanguinoderma rugosum*	Cui 9011	Guangdong, China	KJ531664	–	MG367506	KU572504	[79]
*Tomophagus colossus*	TC-02	Vietnam	KJ143923	–	–	KJ143943	[76]

## 3. Results

In this study, we morphologically and phylogenetically analyze and report 22 Ganoderma species from the GMS, namely, *G. adspersum*, *G. applanatum*, *G. australe*, *G. calidophilum*, *G. ellipsoideum*, *G. flexipes*, *G. gibbosum*, *G. heohnelianum*, *G. hochiminhense*, *G. leucocontextum*, *G. lucidum*, *G. multiplicatum*, *G. multipileum*, *G. myanmarense*, *G. orbiforme*, *G. philippii*, *G. resinaceum*, *G. sichuanense*, *G. sinense*, *G. subresinosum*, *G. williamsianum*, and *G. tsugae*. Of these 22 species, 12 were from Yunnan Province, China; three were from Laos; three species, two new records, and one new species were from Myanmar; 15 species and four new records were from Thailand; and one new species was from Vietnam. The phylogenetic and morphological analyses results of the 22 Ganoderma species are detailed below.

### 3.1. Phylogenetic Analyses

**Figure 3:**
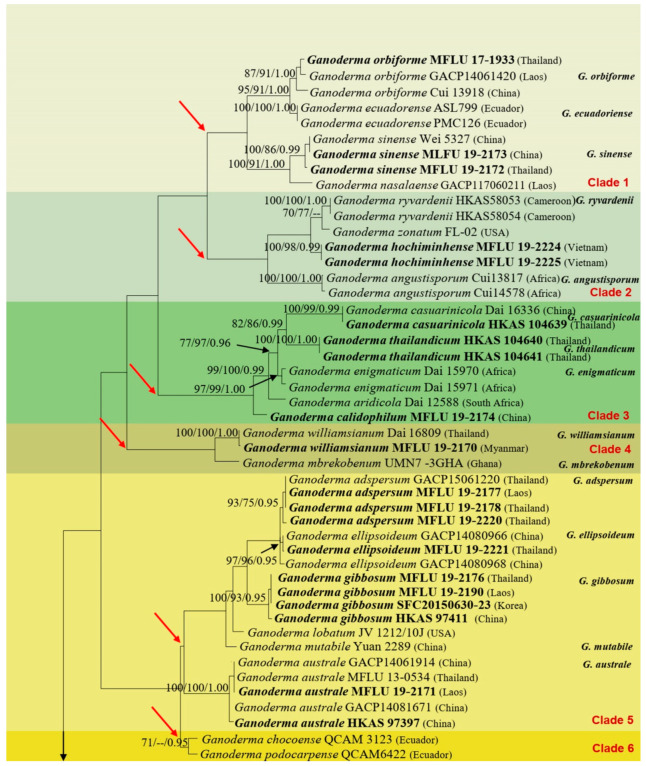

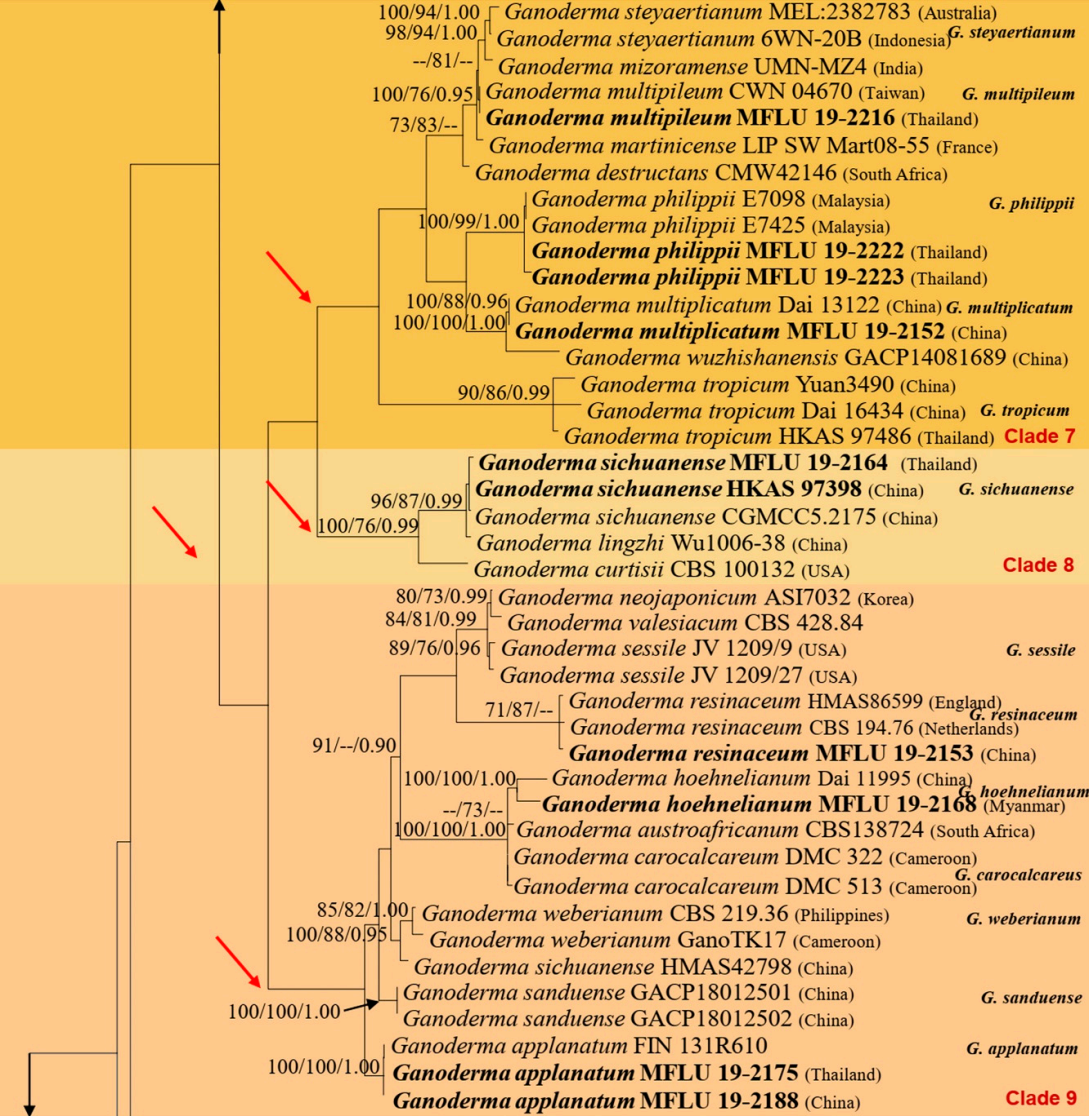

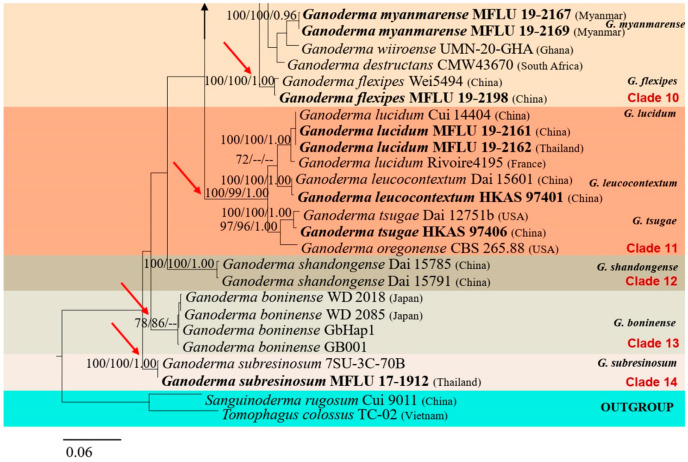
Maximum likelihood phylogenetic tree ML obtained from the DNA sequence data of ITS, LSU, RPB2, and TEF1α data sets. Bootstrap values BS from maximum likelihood ML, left, maximum parsimony MP, middle, equal to or greater than 70% and Bayesian posterior probabilities PP, right, equal to or greater than 0.95 are indicated above or below the nodes as MLBS/MPBS/PP. The tree is rooted with *Sanguinoderma rugosum* Cui 9011 and *Tomophagus colossus* TC-02. New species, new record species, and known species obtained in this study are indicated in bold black.

Phylogenetic analyses were inferred from the combined data set of ITS, LSU, RPB2, and TEF1α sequences of 114 taxa, of which 112 taxa belong to the genus *Ganoderma*, and the remaining 2, *Sanguinoderma rugosum* Blume and T. Nees (Cui 9011) and *Tomophagus colossus* (Fr.) Murrill, Torreya (TC-02) are the outgroup taxa. The data set comprised 3524 characters with gaps (637 characters for ITS, 866 characters for LSU, 1016 characters for RPB2, and 1005 characters for TEF1α), of which 2459 characters were constant, 855 characters were variable and parsimony-informative, and 210 characters were parsimony-uninformative. Tree topologies resulted from the ML analysis were similar to that of the MP and Bayesian analysis. Hence, the best-scoring ML tree is shown in Figure 3.

*Ganoderma* specimens used for this study, based on the collections made from China, Laos, Myanmar, Thailand, and Vietnam, were all placed within the *Ganoderma* clade. The phylogenetic tree includes 14 laccate clades, one non-laccate clade, and an outgroup clade. In this study, of a total of 22 *Ganoderma* species, 13 species (viz. *G*. *australe*, *G*. *calidophilum*, *G*. *flexipes*, *G. gibbosum*, *G. leucocontextum*, *G. applanatum*, *G. lucidum*, *G. multiplicatum*, *G. resinaceum*, *G. sanduense*, *G. sichuanense*, *G. sinense*, *G. tsugae*) from Yunnan Province, China, 3 *Ganoderma* species (*G. adspersum*, *G. australe*, *G. gibbosum*) from Laos, 2 *Ganoderma* species (*G. hoehnelianum*, *G. williamsianum*) from Myanmar, and 11 *Ganoderma* species (*G. adspersum*, *G. applanatum*, *G. ellipsoideum*, *G. gibbosum*, *G. lucidum*, *G. multipileum*, *G. orbiforme*, *G. philippii*, *G. sichuanense*, *G. sinense*, *G. subresinosum*) are reported from Thailand. Furthermore, two taxa, viz. *G*. *myanmarense* from Myanmar and *G. hochiminhense* from Vietnam, are described here as new species. The tree topologies provided considerably high support in the terminal nodes but failed to recover deeper nodes with high statistical support. The details of 13 *Ganoderma* species clades are provided in the following:

Clade 1 was statistically unsupported and comprised four species, viz. *G*. *orbiforme*, *G*. *ecuadorense*, *G*. *sinense,* and *G*. *nasalaense*. Three sequences of the laccate *G*. *orbiforme* from China, Laos, and Thailand clustered together with significant support values (MLBS = 87%/MPBS = 91%/PP = 1.00), suggesting all of them to be the morphotype of the same taxon. However, *G*. *orbiforme* is closely related to *G*. *ecuadorense* but differed by strong statistical support (MLBS = 95%/MPBS = 91%/PP = 1.00). Two isolates of *G*. *sinense* from China and Thailand clustered together (MLBS = 100%/MPBS = 86%/PP = 0.99) and show genotypic closeness with the taxon *G*. *nasalaense* (MLBS = 100%/MPBS = 91%/PP = 1.00). Clade 2 consists of the laccate *G*. *angustisporum*, *G*. *ryvardenii*, *G*. *zonatum*, and *G. hochiminhense* (MFLU 19-2224 and MFLU 19-2225). Two sequences of the newly described species, *G. hochiminhense*, from Vietnam clustered together with strong support values (MLBS = 100%/MPBS = 98%/PP = 0.99) and comes sister to *G*. *ryvardenii* and *G*. *zonatum* although, this position is statistically unsupported.

Clade 3 was strongly supported (MLBS = 97%/MPBS = 99%/PP = 1.00) and contains the laccate *G. casuarinicola* from China (Dai 16336) and Thailand (HKAS 104639), two sequences of *G. thailandicum* (HKAS 104640 and HKAS 104641) from Thailand, *G. enigmaticum* (Dai 15970 and Dai 15971) from Africa, *G. aridicola* (Dai 12588) from South Africa and *G. calidophilum* (MFLU 19-2174) from China. Basal to Clade 3, two species viz. *G. williamsianum* and *G. mbrekobenum* formed Clade 4. The newly generated sequence of *G. williamsianum* from Myanmar (MFLU 19-2170) clustered with the same taxon sequence, previously deposited in the GenBank nucleotide database from Thailand (Dai 16809), with full support values (MLBS = 100%/MPBS = 100%/PP = 1.00). The molecular data confirmed the presence of *G. williamsianum* in Myanmar, and this holds the first distributional record of the taxon from Myanmar.

Clade 5 consists of the non-laccate *G*. *adspersum* from Thailand (MFLU 19-2178 and MFLU 19-2220) and Laos (MFLU 19-2177) (MLBS = 93%/MPBS = 75%/PP = 0.95); holotype sequence of *G*. *ellipsoideum* from China (GACP14080966 and GACP14080968) and Thailand (MFLU 19-2221) (MLBS = 97%/MPBS = 96%/PP = 0.95); *G*. *gibbosum* from Laos (MFLU 19-2190), Thailand (MFLU 19-2176), China (HKAS 97411), and Korea (SFC20150630-23) (MLBS = 100%/MPBS = 93%/PP = 0.95); *G*. *lobatum* (JV 1212/10J) from USA; *G*. *mutabile* (Yuan 2289) from China; and five sequences of *G*. *australe* from China (HKAS 97397, GACP14061914, GACP14081671), Thailand (MFLU 13-0534), and Laos (MFLU 19-2171) (MLBS = 100%/MPBS = 100%/PP = 1.00). However, this clade is statistically unsupported.

Clade 6 is formed by the cluster of Ecuadorean *Ganoderma* species viz. *G. chocoense* (QCAM 3123) and *G. podocarpense* (QCAM6422). This clade was unsupported by the maximum parsimony analysis but weakly supported by the maximum likelihood and Bayesian analyses (MLBS = 71%/PP = 0.95).

Clade 7 is statistically unsupported. This clade comprises of the isolate of the laccate *G. multipileum* from Thailand (MFLU 19-2216) and Taiwan, PRC (CWN 04670) (MLBS = 100%/MPBS = 76/PP = 0.95) and shows its genetic closeness with that of *G. steyaertianum*, *G. mizoramense* and *G. destructans*. Two newly amplified sequences of *G. philippii* from Thailand (MFLU 19-2222 and MFLU 19-2223) clustered with strong support values (MLBS = 100%/MPBS = 99/PP = 1.00) with the same species sequence, previously deposited from Malaysia (E7098 and E7425). The Chinese (Yuan 3490 and Dai 16434) and Thailand (HKAS 97486) strains of *G. tropicum* comes basal to this clade but, this position is not supported statistically. In Clade 8, two newly amplified sequences of *G. sichuanense* from China (HKAS 97398) and Thailand (MFLU 19-2164) clustered with strong support values (MLBS = 96%/MPBS = 87%/PP = 0.99) with the sequence of the same species *G. sichuanense* (CGMCC52175). Moreover, one of the Chinese sequences, *G. lingzhi* (Wu1006-38), clustered with all the three sequences of *G. sichuanense*, we suggesting wrong identification. earlier deposited in GenBank from China, *G. curtisii* stands sister to *G. sichuanense,* and this position is strongly supported (MLBS = 100%/MPBS =76%/PP = 0.99).

Clade 9 comprised five small subclades. The laccate *G. sessile* along with *G*. *neojaponicum* and *G*. *valesiacum* formed a subclade with moderate support (MLBS = 84%/MPBS = 81%/PP = 0.99). Sister to this subclade, there remains another subclade containing the Chinese *G. resinaceum* along with sequences of the same taxon from the Netherlands (CBS 194.76) and England (HMAS86599) but, this position is statistically unsupported. The newly generated sequence of *G. hoehnelianum* from Myanmar (MFLU 19-2168) formed the next subclade with that of the Chinese collection (Dai 11995) and showed its genetic closeness with the laccate *G. carocalcareum* (DMC 322 and DMC 513) and *G*. *austroafricanum* (CBS138724) with full support values (MLBS = 100%/MPBS = 100%/PP = 1.00). This subclade was followed by the cluster of two species viz. *G. weberianum* (CBS 219.36, GanoTK 17) and *G. sichuanense* (HMAS42798), but the position of these two taxa was statistically unsupported. Two Chinese sequences of *G. sanduense* (GACP18012501 and GACP18012502) form the next subclade. The basal subclade contained two newly generated sequences of *G. applanatum* from Thailand (MFLU 19-2175) and China (MFLU 19-2188) along with the previously deposited sequence of the same taxon with strong support values (MLBS = 100%/MPBS = 100%/PP = 1.00). The newly described taxon, *G. myanmarense* from Myanmar (MFLU 19-2167 and MFLU 19-2169), falls in Clade 10 where the new species shows its genetic similarity with that of the laccate *G. wiiroense* from Ghana (UMN-20-GHA) and *G. destructans* from South Africa (CMW43670). *G. flexipes* remains basal to this cluster (Wei5494 and MFLU 19-2198). The newly generated sequence of *G*. *flexipes* (MFLU 19-2198) shows its full identity with one of the previously deposited Chinese *G*. *flexipes* sequences (Wei5494) and clustered with full support values (MLBS = 100%/MPBS = 100%/PP = 1.00).

Clade 11 was enriched with the *G. lucidum* species complex where four sequences of *G. lucidum* from three countries viz. Thailand (MFLU 19-2162), China (MFLU 19-2161 and Cui 14404), and France clustered together (MLBS = 100%/MPBS = 100%/PP = 1.00) and revealed *G. leucocontextum* (Dai 15601 and HKAS 97401), *G. tsugae* (Dai 12751b, HKAS 97406), *G. oregonense* as sister taxa with strong support values (MLBS = 100%/MPBS = 99%/PP = 1.00).

Two sequences of the taxon *G. shandongense* (Dai 15785 and Dai 15791) formed Clade 12. This small clade was followed by another clade, Clade 13, which comprised of four sequences of *G. boninense* (Clade 13).

Clade 14 was the extreme basal clade where the sequence of *G. subresinosum* (MFLU 17-1912), collected from Thailand (MFLU 17-1912), clustered with full support (MLBS = 100%/MPBS = 100%/PP = 1.00) with one of the earlier sequences of the same taxon, deposited in the nucleotide sequence database.

### 3.2. Taxonomy

***Ganoderma*** P. Karst., Revue Mycologique Toulouse. 3(9): 17 (1881)

= *Dendrophagus* Murrill, Bull. Torrey bot. Club. 32(9): 473 (1905)

= *Elfvingia* P. Karst., Bidr. Känn. Finl. Nat. Folk. 48: 333 (1889)

= *Friesia Lázaro Ibiza*, Revista Real Acad. Ci. Madrid. 14: 587 (1916)

= *Ganoderma* subgen. Trachyderma Imazeki, Bull. Tokyo Sci. Mus. 1: 49 (1939)

= *Tomophagus* Murrill, Torreya. 5: 197 (1905)

= *Trachyderma* (Imazeki) Imazeki, Bull. Gov. Forest Exp. Stn Tokyo. 57: 97 (1952)

Type species: *Ganoderma lucidum* (Leyss: Fr.) Karst.

Notes: (≡) is homotypic, or nomenclatural, synonyms, (=) is heterotypic, or taxonomic, synonyms.

*Description*: Basidiomes annual, dimidiate, sessile or sub-stipitate to stipitate. Pileus subdimidiate to dimidiate, flabelliform, perennial, stipitate or sessile. Pileus surface non-laccate (dull) or weakly to strongly laccate, glossy, shiny, smooth, spathulate, shallow, furrows, sulcate, several layers thick, with thin- to thick-cuticle cells or cuticle of clavate end cells, thicker at the base than the margin, thin- to thick-crust overlaying the pileus, consistency hard, consistency hard, light weight when dried. Pileus color variable, light yellow to yellow, light brown, slightly brown to dark brown, sometimes homogeneous reddish gray to reddish-yellow. Context brown to dark brown, grayish orange to orange, sometimes grayish-yellow, mostly soft, sometimes spongy to firm-fibrous. Hymenophore di-trimitic, heterogeneous, non-septate or septate, usually yellow, slightly light orange, or light brown to brown, sometimes with melanoid bands. Tubes are hard, woody when dried. Tube layers single or stratified, pale to purplish-brown, almost hyaline with clamp connections, occasionally branched at apex, thin- to thick-walled. Stipe central or lateral, glossy with a distinct cuticle. Margin actively growing, entirely white when fresh, round, soft and smooth when young, slippery when touched from youth to maturity, and tough when broken. Pores 4–7 in number per mm, angular, entire, subcircular to circular, regular, cream or white when young, light yellow, light orange to brown when mature. Pore surface usually white to cream when fresh, turning yellowish-white to pale yellow on drying, some sections reddish gray to brown, and brownish gray when wet.

*Hyphal structure*: Hyphal system di-trimitic, including generative, skeletal, and binding hyphae; mostly generative hyphae with clamp connections, hyaline, brown, non-septate, or septate, often with long and tapering branches. Basidia broadly ellipsoid, tapering abruptly at the base. Cystidia absent. Basidiospores broadly to narrowly ellipsoid or oblong, sometimes globose to subglobose, with double walls, truncate apex, apical germ pore present, usually with light brown to brown endosporium, hyaline exosporium with thin inter-walled pillars, hyaline endosporium with thick outer walls, and some very thin exosporium.

*Ecology*: mostly on hardwoods, trunks, and stumps, occurring on several different living tree host species.

*Notes*: Justo et al. [103] treated Ganodermataceae as a synonym of Polyporaceae and included the genus *Ganoderma* under Polyporaceae. Later, Cui et al. [104] excluded *Ganoderma* from Polyporaceae due to the presence of double-walled basidiospores, unlike Polyporaceae. So, the distinctiveness of the genus *Ganoderma* lies in the presence of double-walled and truncate basidiospores. Species with a laccate, glossy surface are present in both Ganodermataceae and Polyporaceae as centrally and laterally stipitate species.

#### 3.2.1. Taxonomy of *Ganoderma* from China

***Ganoderma angustisporum*** J.H. Xing, B.K. Cui and Y.C. Dai, Mycokeys 34: 98 (2018)

Taxonomy and phylogenic analysis were described in Xing et al. [37]

*Notes: Ganoderma angustisporum* is characterized by annual, sessile, broadly basidiomes, strongly laccate on the upper surface of basidiomes, white pore surfaces, and almond-shaped, slightly truncate, narrow 9.0–11.3 × 4.0–5.2 µm basidiospores. It is a group of white-rot fungi that predominantly grow on living *Casuarina equisetifolia* in Fujian Province, China.

**Figure 4:**
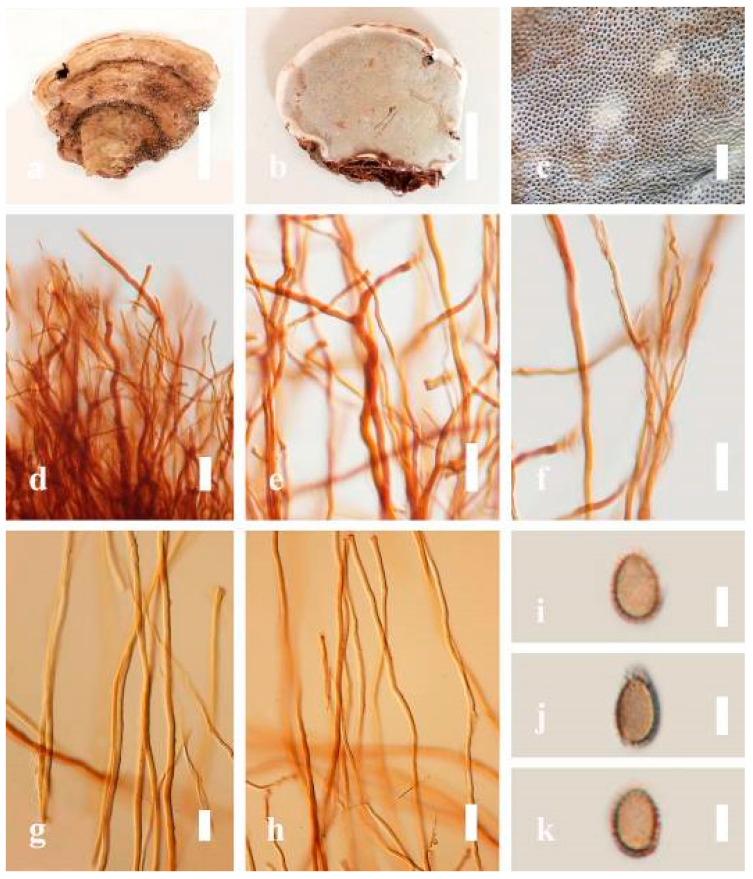
Morphology of *Ganoderma applanatum* (HKAS 107254, MFLU 19-2188): (**a**,**b**) mature basidiomes; (**c**) pore characteristics; (**d**–**f**) hyphae of trama in KOH; (**g**,**h**) generative hyphae of context in KOH; (**i**–**k**) basidiospores. Scale bars: (**a**,**b**) = 2 cm, (**c**) = 1000 µm, (**d**–**h**) = 20 µm, (**i**–**k**) = 5 µm.

***Ganoderma applanatum*** (Pers.) Pat., Hymenomyc. Eur. (Paris): 143 (1887) (Figure 4)

≡ *Boletus lipsiensis* Batsch, Elenchus fungorum. Continuatio prima.: 183, t. 25:130 (1786)

≡ *Scindalma lipsiense* (Batsch) Kuntze, Revisio generum plantarum. 3(2): 518 (1898)

≡ *Polyporus lipsiensis* (Batsch) E.H.L. Krause, Basidiomycetes Rostochienses.: 54 (1928)

≡ *Agaricus lipsiensis* (Batsch) E.H.L. Krause, Basidiomycetum Rostochiensium, Suppl. 4: 142 (1932)

= *Boletus applanatus* Pers., Observationes mycologicae. 2: 2 (1799)

= *Polyporus merismoides* Corda, Deutschlands Flora, Abt. III. Die Pilze Deutschlands. 3: 139 (1837)

= *Polyporus stevenii* Lév., Annls Sci. nat., Bot.: 91 (1844)

= *Polyporus leucophaeus* Mont., Sylloge generum specierumque plantarum cryptogamarum.: 157 (1856)

= Polyporus leucophaeum Mont. (1856)

= *Polyporus incrassatus* Berk., Journal of the Linnean Society. Botany. 16: 41 (1877)

= *Polyporus concentricus* Cooke, Grevillea. 9(49): 13 (1880)

= *Fomes gelsicola* Berl., Malpighia. 3: 373 (1889)

= *Fomes nigriporus* Lázaro Ibiza, Revista de la Real Academia de Ciencias Exactas Fisicas y Naturales Madri. 14: 662 (1916)

= *Ungularia subganodermica* Lázaro Ibiza, Revista de la Real Academia de Ciencias Exactas Fisicas y Naturales Madri. 14: 674 (1916)

= *Fomes longoporus* Lloyd, Mycological Writings. 6(62): 940 (1920)

Facesoffungi number: FoF 06249

*Description*: Basidiomes annual, perennial, sessile. Pileus 1.5–5.8 cm in length, 0.5–4.5 cm in width, and up to 1.5 cm thick at the base, sessile (without stipe), perennial, subdimidiate, sub-flabelliform to flabelliform, usually flat, convex, imbricate, umbonate or uneven, rarely ungulate, glabrous when present, broadly attached when mature, often with undefined concentric zones at the center that extend to the margin, thick at the base, slightly soft at the margin when mature. Pileus surface shiny, silky, smooth, and soft when young, hard when old, frequently furrowed and shallow sulcate, undulating, somewhat spathulate to uneven on the upper surface when mature, covered by irregularly ruptured thick crust, slightly non-laccate (dull) and faded from when mature to old, compact and hard when mature, woody to corky when old. Pileus color is usually homogenous with grayish-orange (6B3–6B5) at the center, slight brownish-orange (6C4), orange white (6A2), to pale orange (6A3), with yellowish-gray (4B2) at the margin when mature. Context up to 0.3–1 cm thick at the base, mostly light brown (7D5), brown (6E8) to dark brown (7F6–7F8) of cuticle cells, with walls varying in thickness to subsolid hyphae, some fibrous pithy context, usually separated by layers of context tissue at the base, and some occurred woody lines. Tube woody, hard, often dark brown (7F7–7F8) when dried, with sulcate at different levels. Stipe almost sessile and broadly attached when present, with a differentiated zone at the point of attachment. Margin up to 1 cm thick, white (5A1), yellowish-gray (4B2) when mature, turns light brown (6D4) to brown (6E8) when scratched or bruised, often slippery when wet, soft when young, thinner than the center. Pore 4–6 in number per mm, subcircular to circular, sometimes angular. Pore surface initially white (7A1), grayish-orange (7C3–7C4) when mature, turning to light brown (7D6) to brown (7D8) when scratched or bruised.

*Hyphal structure:* Hyphal system trimitic; generative hyphae 0.8–2.6 µm ($\bar{x}$ = 2.1, *n* = 30) in diam, almost hyaline, with clamp connections, abundant, thin-walled and occasionally thick-walled; skeletal hyphae 2.1–4.6 µm width (*n* = 30), usually thick-walled, hyaline, sometimes branched; binding hyphal 1.6–3.3 µm width (*n* = 30), thick-walled and occasionally thin-walled, branched, and intertwined with the skeletal hyphae. Pileipellis a hymeniderm, grayish brown (6E4), which is composed of apically acanthus-like branched cells. Basidiospores mostly ellipsoid with double walls, with a size range of (9.8–)10.4–*11.1*–11.9(–12.1) × (7.3–)8.0–*8.6*–9.2(–9.9) μm, ($\bar{x}$ = 11.3 × 8.7 μm, *n* = 50) μm, with Q = 1.79–1.86, L = 11.23 µm, W = 6.12 µm (including myxosporium), (6.2–)7.6–*8.6*–9.7(–10.4) × (5.0–)5.8–*7*.1–8.2(–8.9) μm ($\bar{x}$ = 8.6 × 7.1 μm, *n* = 50) μm, with Q = 1.19–1.24, L = 8.59 µm, W = 7.12 µm (excluding outer myxosporium), brownish-orange (7D4) to brown (7D7–7D8) in KOH, and reddish-brown (8E6) to dark brown (8F4) in Melzer’s reagent. Basidia 14–20 × 8–10 μm, with four sterigmata.

Ecology: Solitary on stump of *Machilus yunnanensis*.

*Specimens examined:* CHINA, Yunnan Province, Baoshan, 25°09′35″ N, 99°09′49″ E, 1973 m elev., 11 November 2017, T. Luangharn, HKAS 107254, MFLU 19-2188.

*Notes: Ganoderma lipsiense* has been treated by some researchers as the correct name for *G. applanatum* [15]. *G. applanatum* (=*G. lipsiense*) belongs in the subgenus *Elfvingia*, which is characterized by distinctive non-laccate species, a thin and acute margin of the pileus, and unbranched terminal endings of skeletal hyphae, with ellipsoid basidiospores [47,105,106,107]. *G. applanatum* causes white butt rot on angiosperm trees and is widely distributed in China [64]. Hence, our specimen of *G. applanatum*, collected from a temperate region of China, is described based on morphological characteristics and molecular phylogenetic data. Our results agree well with those of Ryvarden and Gilbertson [105].

**Figure 5:**
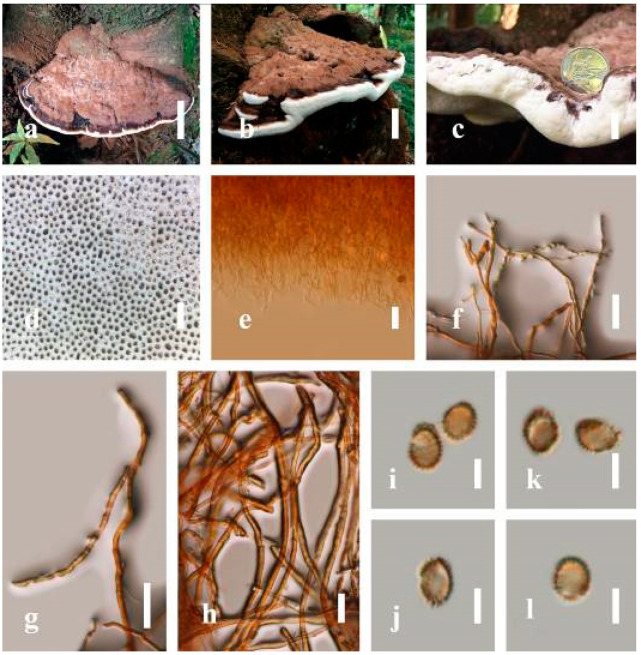
Morphology of *Ganoderma australe* (HKAS 97397): (**a**,**b**) mature basidiomes; (**c**) margin; (**d**) pore characteristics; (**e**) mycelia of tube layers; (**f**–**h**) context hyphae as seen in Melzer’s reagent; (**i**–**l**) basidiospores. Scale bars: (**a**) = 5 cm, (**b**) = 3 cm, (**c**) = 1 cm, (**d**) = 500 µm, (**e**) = 20 µm, (**f**) = 20 µm, (**g**,**h**) = 10 µm, (**i**–**l**) = 5 µm.

***Ganoderma australe*** (Fr.) Pat., Bull. Soc. mycol. Fr. 5(2–3): 65 (1889) (Figure 5)

≡ *Polyporus australis* Fr., Elenchus Fungorum. 1: 108 (1828)

≡ *Fomes australis* (Fr.) Cooke, Grevillea. 14(69): 18 (1885)

≡ *Placodes australis* (Fr.) Quél., Enchiridion Fungorum in Europa media et praesertim in Gallia Vigentium. 171 (1886)

≡ *Fomes applanatus* var. australeis (Fr.) Cleland and Cheel, Journal of Proceedings of the Royal Society of New South Wales. 51: 518 (1918)

≡ *Ganoderma applanatum* subsp. *australe* (Fr.) Bourdot and Galzin, Bulletin de la Société Mycologique de France. 41: 184 (1925)

≡ *Ganoderma applanatum* f. *australe* (Fr.) Bourdot and Galzin, Bulletin de la Société Mycologique de France. 41: 184 (1925)

≡ *Elfvingia australis* (Fr.) G. Cunn., Bulletin of the New Zealand Department of Industrial Research. 164: 256 (1965)

= *Polyporus tornatus* Pers., Botanique (Nagpur). 5: 173 (1827)

= *Polyporus scansilis* Berk., Journal of the Linnean Society. Botany. 16: 53 (1877)

= *Fomes annularis* Lloyd, Mycol. Writ. 4(40): 6 (1912)

= *Ganoderma tornatum* var. *tornatum* (Pers.) Bres., Hedwigia. 53(1–2): 55 (1912)

= Fomes konigsbergii Lloyd (1915)

= *Fomes polyzonus* Lloyd, Synopsis of the genus Fomes. (7): 269 (1915)

= *Fomes pseudoaustraleis* Lloyd, Synopsis of the genus Fomes. (7): 269 (1915)

= *Fomes undatus* Lázaro Ibiza, Revista de la Real Academia de Ciencias Exactas Fisicasy Naturales Madri. 14: 661 (1916)

Facesoffungi number: FoF 06242

*Description*: Basidiomes annual, perennial, subdimidiate, sessile. Pileus 14–28 cm in length, 12–32 cm in width, and 1.4–3.2 cm thick. Pileus flabelliform, spathulate, subdimidiate, umbonate, single, sulcate, large, obtuse from host, radial from the center extending the margin, broadly attached, often thick at the center, slightly soft at the margin, consistency hard, and tough to break when dried. Pileus surface convex, corky, furrowed, spathulate, mostly umbonate or uneven, non-laccate (dull) on maturity or in old, usually slippery where the new hyphae are in active development (margin), slightly concentrically sulcate at the center toward margin, smooth, covered with thick and hard crust, irregularly ruptured crust overlying the surface, woody, and corky when dried, with cracked crust when mature, and tough to break when dried. Pileus color often brown (6E7–6E8) at the base, reddish-orange (7B7–7B8), brownish-orange (7C6–7C8), almost covered with grayish-red (8C4–8C5) on the upper surface when old, slight reddish-brown (8F8, 9E7–9E8) close to the margin. Context up to 0.5–2 cm thick near stipe, fibrous, composed of coarse loose fibrils, brown (6D7–6D8) to dark brown (6F7), with reddish-brown (8D8–8D9) coarse loose fibrils, covered with thick crust. Tube 0.4–1.5 cm long, brown (7D8) to dark brown (6F8). Stipe sessile, broadly attached. Margin soft when young, slippery when fresh, blunt when mature, white (4A1). Pore 4–6 in number per mm, subcircular to circular, sometimes angular. Pore surface initially white (4A1), slight to pale yellow (4A3) when mature, turned brownish-orange (6C7–6C8) when scratched or bruised or discolored when touched.

*Hyphal structure:* Hyphal system trimitic, dense and hard, thick-walled, typically with narrow lumen, flexuous, and many branches, usually brownish-orange (6C5–6C7) in KOH; generative hyphae 2.2–3.8 µm broad (*n* = 30), thin-walled, hyaline, tapering at branch, with clamp connections; skeletal hyphae 2.9–4.2 µm broad (*n* = 30), sometimes branched, nearly solid, thick-walled; binding hyphae 2.6–4.0 µm broad (*n* = 30), thick-walled, branched, more or less solid; hymenial with sword-like apices in the context. Pileipellis a hymeniderm, usually brownish-orange (6C5) to brown (6E8), composed of apically acanthus-like branched cells. Basidiospores mostly ellipsoid to broadly ellipsoid, double walls, (6.2-)7.1–*9.4*–10.4(-11.8) × (5.2-)6.0–*7.4*–8.9(-9.7) μm ($\bar{x}$ = 9.4 × 7.4 μm, *n* = 50) μm, with Q = 1.24–1.30, L = 9.42 µm, W = 7.43 µm (including myxosporium), (5.3-)6.7–*7.8*–9.6(-10.5) × (4.5-)5.1–*5.7*–6.3(-7.2) μm ($\bar{x}$ = 7.8 × 5.7 μm, *n* = 50) μm, with Q = 1.31–1.38, L = 7.85 µm, W = 5.83 µm (excluding outer myxosporium), overlaid by hyaline, apically brown, bearing a fine, distinct, short, echinulae truncate, turgid vesicular appendix, inner wall orange (6B8) to brownish-orange (6C8) or light brown (7D5–7D6) to brown (7E7–7E8), outer wall mostly reddish-brown (8E7–8E8, 8F7) in 5% KOH.

*Habitat:* Solitary, growing on living *Neocinnamomum delavayi* (Lec.) H. Liou. tree or, decaying stump, and living *Fagus* spp.

*Specimens examined:* CHINA, Yunnan Province, Kunming Botanical Garden, 25°08′39″ N, 102°44′30′ E, 1956 m, 27 September 2016, T. Luangharn, HKAS 97397.

*Notes: Ganoderma australe* belongs under the subgenus *Elfvingia* [38]. This fungus was initially described from the Pacific Islands [108]. *G. australe* belongs to the *G. applanatum*-*australe* complex [2]. Ganoderma australe was established as a non-laccate (dull) pilei. The type specimen of this fungus is missing, while the neotype specimen is available in Europe [105]. *Ganoderma applanatum* and *G. australe* from Europe have been confused based on the macro-characteristic features [107]. The typification of *G. australe* remains unresolved and was exemplified by several authors [61,109,110,111], and its similar cultural characteristics also showed the phenotypic plasticity in morphological level and a higher level of nucleotide divergence in the ITS rDNA region that made *G. australe* a complex species [45].

*Ganoderma australe* is distinguished from *G. applanatum* by the larger dimensions of its basidiospores, different stipe features, thickness of the cuticle, and color of the context layer, all of which were considered in delimiting *G. applanatum* and *G. australe* [2,105,107]. *G. applanatum* is confined to northern temperate regions, while *G*. *australe* can be found in tropical and subtropical regions [112,113]. There are reports on the occurrence in Australia [14], China [30], New Zealand [114], southern India [51,95], Taiwan, PRC [115], and Thailand [30,59]. *Ganoderma australe* is a cosmopolitan species, which is known to cause white rot on woody material. It shows parasitic or pathogenic behavior on a wide range of both dead and living broadleaved deciduous trees [116,117,118]. *Ganoderma australe* is distributed worldwide, especially in tropical regions [14].

**Figure 6:**
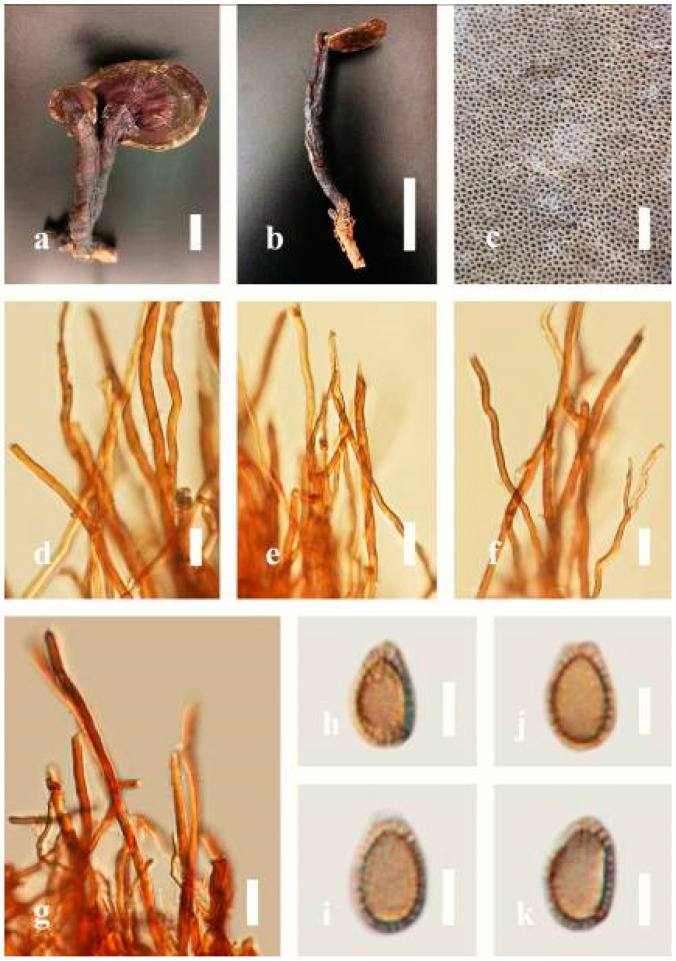
Morphology of *Ganoderma calidophilum* (MFLU 19-2174): (**a**,**b**) mature basidiomes; (**c**) pore characteristics; (**d**–**f**) context hyphae in Melzer’s reagent; (**g**) tube layer hyphae in Melzer’s reagent; (**h**–**k**) basidiospores. Scale bars: (**a**) = 2 cm, (**b**) = 5 cm, (**c**) = 1000 µm, (**d**–**g**) = 20 µm, (**h**–**k**) = 5 µm.

***Ganoderma calidophilum*** J.D. Zhao, L.W. Hsu and X.Q. Zhang, Acta Mycologica Sinica 19: 270 (1979) (Figure 6)

Facesoffungi number: FoF 06244

*Description*: Basidiomes annual, stipitate, subdimidiate. Pileus 3–7 cm in length, 2–4 cm in width, and 0.2–1 cm thick. Pileus subdimidiate to dimidiate, spathulate, stipitate, sulcate, umbonate, radial from the center extending to the margin, tough to break when dried, often thick at the center, slightly soft at the margin, and light in weight when dried. Pileus surface corky, convex, furrowed, glabrous, glossy, incised, shiny, spathulate, shallow, sulcate when fresh, umbonate or uneven, laccate and glossy when mature, weakly laccate when old and in regions of developing hyphae (margin), slightly concentrically sulcate, layers smooth at the center when young, irregularly ruptured crust overlying the context, and tough to break when dried. Pileus color usually homogenous with brownish-red (8C7–8C8), brownish-red (9C7–9C8), reddish-brown (9D6–9D7) center, extending brownish-orange (6C7–6C8) toward the stipe, brownish-red (9C8) from the center to light brownish-orange (6C8), and usually light brown (6D8) at the margin when old. Context up to 0.2–0.6 cm thick near stipe, dry, fibrous, composed of coarse loose fibrils, brownish-orange (6C5–6C8) upper layers when fresh, brown (6D7) at lower layers, dark brown (8F7) when dried, covered with thin crust, trimitic hyphal system. Tube 0.3–0.9 cm in length, brown (7D8). Stipe 5–14 cm long, cylindrical, almost stipitate with broadly, irregularly ruptured crust overlying, strongly laccate with brown (7D8) when mature, dark brown (8F8) when old, and woody or corky when dried. Margin soft when young, laccate when mature, weakly laccate to laccate when old, blunt when old, usually light brown (6D8) when mature to old. Pore 4–5 in number per mm, subcircular to circular, sometimes angular. Pore surface initially pale orange (5A3) to brownish-orange (6D8) when mature, discolored when touched, brown (6E8) when scratched or bruised.

*Hyphal structure:* Hyphal system trimitic hyphal, usually brownish-orange (6C5–6C7) in KOH; generative hyphae 1.2–3.2 µm broad (*n* = 30), thin-walled, hyaline, without clamp connections; skeletal hyphae 3.2–6.4 µm broad (*n* = 30), sometimes branched, nearly solid, thick-walled, without clamp connections; binding hyphae 2.4–5.2 µm broad (*n* = 30), usually thin to thick-walled, many branches, nearly solid, hymenial with sword-like apices in the context. *Basidiospores* mostly ellipsoid to broadly ellipsoid, with double wall, with a size range of (7.4-)8.5–*11.9*–12.6(-13.7) × (6.3-)7.2–*8.3*–9.1(-9.6) μm ($\bar{x}$ = 11.9 × 8.3 μm, *n* = 50) μm, with Q = 1.39–1.45, L = 11.92 µm, W = 8.35 µm (including myxosporium), (6.8-)7.6–*10.4*–11.3(-12.8) × (5.4-)6.3–*7.0*–7.6(-8.1) μm ($\bar{x}$ = 10.4 × 7.1 μm, *n* = 50) μm, with Q = 1.43–1.49, L = 10.39 µm, W = 7.12 µm (excluding outer myxosporium), overlaid by hyaline, apically and echinulae, truncate, turgid vesicular appendix, inner wall orange (6B8) to deep orange (6A8), reddish-orange (7A8, 7B7–7B8) or yellowish-red (8B8), outer wall usually reddish-brown (8D7–8D8; 8E8) in 5% KOH.

*Habitat*: Solitary, near the hardwood root of *Castanopsis* spp., living tree of *Machilus yunnanensis*.

*Specimens examined*: CHINA, Yunnan Province, Baoshan, 25°06′29″ N, 99°08′29″ E, 1973 m elev., 11 November 2017, T. Luangharn, MFLU 19-2174.

*Notes: Ganoderma calidophilum* is a species originally described from Hainan Province, China, by Zhao [119]. Several reports have confirmed that this fungus is mentioned in Hainan Province polypore diversity checklists [13,61,120,121]. This fungus is distinctive in these forms, featuring a laccate pileus with broadly ellipsoid basidiospores with double walls, and it is widely found across subtropical and tropical Asia [108,119,122]. Wang and Wu [112] suggested that *G. calidophilum* is a synonym of *G. flexipes*. However, the evaluated *G. calidophilum* and *G. flexipes* are different in terms of pileus color, pileus shape and size, context, and basidiospore size [112]. In this study, we present our *G. calidophilum* collection from Yunnan Province, China, based on taxonomic and phylogenetic analyses. Our strain is similar to the described strain of Wang and Wu [119], Zhao et al. [119], and Bi et al. [123].

**Figure 7:**
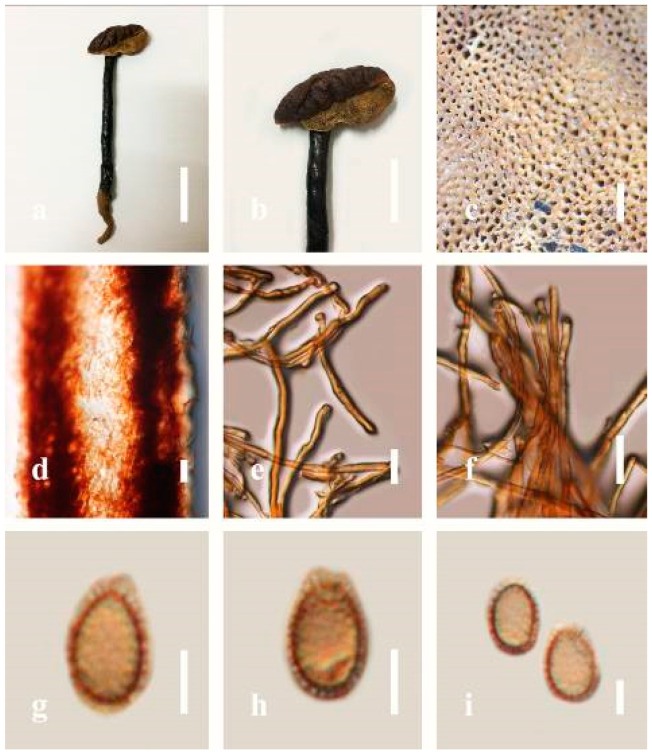
Morphology of *Ganoderma flexipes* (MFLU 19-2189): (**a**,**b**) mature basidiomes; (**c**) pore characteristics; (**d**) hyphae of tube layers in Melzer’s reagent; (**e**,**f**) context hyphae in Melzer’s reagent; (**g**–**i**) basidiospores. Scale bars: (**a**) = 3 cm, (**b**) = 2 cm, (**c**) = 500 µm, (**d**) = 30 µm, (**e**,**f**) = 20 µm, (**g**–**i**) = 5 µm.

***Ganoderma flexipes*** Pat., Bulletin de la Société Mycologique de France. 23(1): 75 (1907) (Figure 7)

≡ *Fomes flexipes* (Pat.) Sacc. and Traverso, Sylloge Fungorum. 19: 710 (1910)

≡ *Polyporus flexipes* (Pat.) Lloyd, Synopsis of the stipitate Polyporoids. (7): 104 (1912)

Facesoffungi number: FoF 06245

*Description:* Basidiomes annual or perennial, stipitate. Pileus 0.5–3.2 cm in length, 0.5–3 cm broad, up to 0.5 cm thick at the base. Pileus stipitate, sub-reniform to reniform, or subflabellate to flabellate, concentrically sulcate zones with tuberculate, glabrous when young to maturity, bumps when mature, often tough to break when dried. Pileus surface shiny, smooth, and soft when young, frequently furrowed and shallow sulcate on upper surface, undulating, somewhat spathulate to uneven when mature, covered by an irregularly ruptured thin crust, faded or weakly laccate when young, and laccate when mature., and woody when old. Pileus color usually homogenous with reddish-brown (8E8) to dark brown (9F7–9F8) at the center, slight to the margin from mature to old. Context up to 0.1–0.6 cm thick at the base, very dry, brown (7D7–7D8) to reddish-brown (8E7), containing fibrous pithy context and corky when old. Tube hard, often dark brown (7F8). Stipe almost 3–12 cm in length, 0.3–1.5 in width, sub-cylindrical to cylindrical, often dark brown (7F8), and strongly laccate from mature to old. Margin soft when young, laccate when mature, some wavy, often light brown (6D5–6D6) on the upper surface. Pore 4–5 in number per mm, subcircular to circular, sometimes angular. Pore surface initially grayish-orange (6B4–6B6), turns brown (7D7) to reddish-brown (8D5–8D7) when scratched or bruised, discolored when touched.

*Hyphal structure:* Hyphal system trimitic, bearing clamp connections, hyaline, thick-walled, tapering branch, some swollen differentiated zone at the point of attachment; generative hyphae (1.8-)2.2–*2.9*–3.4(-3.8) μm broad (*n* = 30), thin-walled, hyaline, unbranched, with clamp connections; skeletal hyphae (3.0-)3.8–*4.8*–5.4(-6.2) μm broad (*n* = 30), with walls varying in thickness, with subsolid, binding hyphae (2.2-)2.8–*3.8*–4.5(-5.1) μm broad (*n* = 30), usually thick-walled, appearing alongside Bovista hyphae, and many branches, usually light yellow (4A4–4A5) to yellowish-orange (4B8) of thin-walled and orange (6A7) to deep orange (6A8) of thick-walled in Melzer’s reagent. Pileipellis a hymeniderm, dark brown (6F8), composed of apically acanthus-like branched cells. Basidiospores mostly ellipsoid to broadly ellipsoid with double wall at maturity, (8.1-)8.8–*9.7*–10.6(-11.2) × (6.1-)6.6–*7.7*–9.7(-10.4) μm ($\bar{x}$ = 9.7 × 7.7 μm, *n* = 50) μm, with Q = 1.08–1.15, L = 9.68 µm, W = 7.72 µm (including myxosporium), (7.3-)7.8–*8.3*–8.7(-9.2) × (4.0-)4.6–*5.4*–5.8(-6.2) μm ($\bar{x}$ = 10.2 × 6.4 μm, *n* = 50) μm, with Q = 1.51–1.57, L = 8.34 µm, W = 5.39 µm (excluding outer myxosporium), overlaid by hyaline, dextrinoid, echinulae, inner wall echinulate brown (5D8, 7E6–7E8), and outer wall usually dark brown (7E8) to reddish-brown (8E6–8E8) in Melzer’s reagent.

*Habitat:* Solitary on the decaying hardwood of *Pinus* spp.

*Specimens examined:* CHINA, Yunnan Province, Baoshan, 25°06′29″ N, 99°08′29″ E, 1973 m elev., November 2017, T. Luangharn, MFLU 19-2189.

*Notes: Ganoderma flexipes* is originally described from Vietnam by Patouillard [124]. It has been recorded from China, India, Laos, Nepal, and Pakistan [4,13,30,110,122]. *G. flexipes* is characterized by its small reddish-brown pileus, long and thin stipe, usually reddish-brown to dark brown context, and ellipsoid or ovoid basidiospores [125]. Among the Chinese *Ganoderma* species, *G. flexipes* is one of the most similar species to *G. sichuanense* as they share a reddish-brown pileal surface, similar basidiospores, and cuticle cells [4]. Our *G. flexipes* from China is very similar to the description of Ryvarden [125] and Hapuarachchi et al. [30], and basidiospores are within the range of 9.7–10.2 × 6.4–7.7 μm.

**Figure 8:**
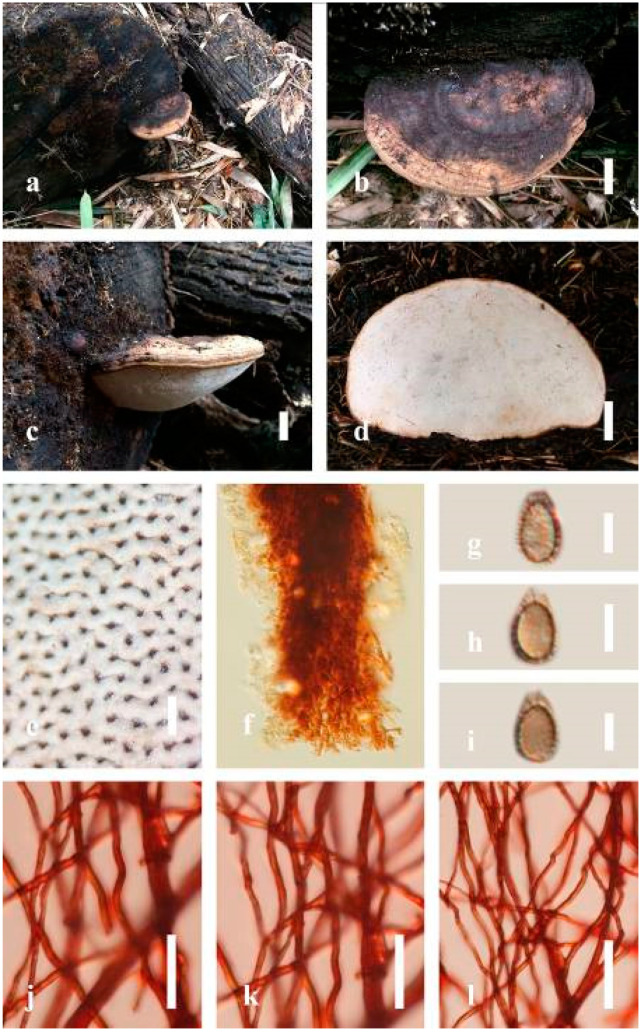
Morphology of *Ganoderma gibbosum* (HKAS 97411): (**a**,**b**) upper surface of mature basidiomes; (**c**) margin; (**d**) under surface of mature basidiomes; (**e**) pore characteristics; (**f**) hyphae of tube layers in Melzer’s; (**g**–**i**) basidiospores in Melzer’s; (**j**–**l**) context hyphae in Congo red. Scale bars: (**b**,**d**) = 3 cm, (**c**) = 1 cm, (**e**) = 500 µm, (**g**–**i**) = 5 µm, (**j**–**l**) = 20 µm.

***Ganoderma gibbosum*** (Blume and T. Nees) Pat., Ann. Jard. Bot. Buitenzorg, suppl. 1: 114 (1897) (Figure 8)

≡ *Polyporus gibbosus* (Blume and T. Nees)., Nov. Act. Academiae Caesareae Leopoldino Carolinae Germanicae Naturae Curiosorum. 13: 19, t. 4(1–4) (1826)

≡ *Fomes amboinensis* var. *gibbosus* (Blume and T. Nees) Cooke, Grevillea. 13(68): 118 (1885)

≡ *Fomes gibbosus* (Blume and T. Nees) Sacc. Syll. Fung. 6: 156 (1888)

≡ *Scindalma gibbosum* (Blume and T. Nees) Kuntze., Revisio generum plantarum 3(2): 518 (1898)

Facesoffungi number: FoF 06246

*Description:* Basidiomes annual or perennial, sessile. Pileus 8–21 cm in length, 6–13 cm in width, and 1–3.5 cm thick, convex, imbricate, umbonate, uneven, ungulate, subflabellate, subdimidiate, usually round when present, primordial, somewhat round and plump when young, somewhat imbricate, when seen from above flabelliform (fan-shaped), broadly attached, thick at the base, slightly soft at the margin when mature. Pileus surface non-laccate (dull), furrowed, incised, sulcate, smooth when young, usually silky, soft, and slippery surface when fresh, undulating on the upper surface, somewhat spathulate to uneven, with a crust (0.2–0.4 mm), woody from mature to older, and lined or cracked crust occurs when old. Pileus color usually homogenous with grayish-orange (6B3–6B6), brownish-orange (6C5–6C6), and brown (6D7–6D8) at the base extending to the margin of mature fruiting bodies. Context up to 0.5–1.8 cm thick, compact and hard, trimitic hyphal, with clamp connections, hyaline, with walls varying in thickness with simple septa, composed of narrow and sparingly branched; generative hyphae 1.3–3.2 µm broad (*n* = 30) with hyaline; skeletal hyphae 2.8–4.7 µm broad (*n* = 30), usually thick-walled; binding hyphal 2.1–4.2 µm width (n = 30) with walls varying in thickness. Hymenophore reddish-brown (8D7). Tube layers 0.4–1.2 cm in length. Stipe almost sessile and broadly attached when present. Margin blunt-edged, wavy, slippery from young, softer, and often white (8A1) when youth to maturity, and light brown (6D5) when old, the present yellow line between the edge of the margin and close to the underside of basidiomes. Pore 4–7 in number per mm, subcircular to circular. Pore surface white (11A1) when present, pale yellow (4A3) to grayish-yellow (4B3–4B4) when scratched or bruised, discolored when touched.

*Hyphal structure:* Hyphal system trimitic hyphal, with clamp connections, usually reddish-brown (8D7–8D8); generative hyphae (1.2-)1.5–2.4–3.0(-3.6) μm broad (*n* = 30), thin-walled and hyaline; skeletal hyphae (2.7-)3.2–3.6–4.2(-4.8) μm broad (*n* = 30), dextrinoid, abundant thick wall; binding hyphae (2.5-)3.0–3.5–4.0(-4.4) μm broad (*n* = 30), thick wall, branched, usually intertwined the generative and skeletal hyphae, mostly dark brown near the tube layers, appearing alongside Bovista-type ligative hyphae, hymenial, sword-like apices at the context. Pileipellis a hymeniderm, dark brown (6D8), composed of apically acanthus-like branched cells, dextrinoid. Basidiospores mostly ellipsoid to broadly ellipsoid or oblong with double walls, (5.8-)6.2–7.2–8.4(-9.2) × (5.4-)5.7–5.4–6.8(-7.7) μm ($\bar{x}$ = 7.3 × 5.6 μm, *n* = 50) μm, with Q = 1.48–1.52, L = 7.32 µm, W = 5.68 µm (including myxosporium), (4.8-)5.2–6.0–6.7(-7.2) × (4.6-)4.9–5.5–5.7(-6.2) μm ($\bar{x}$ = 6.2 × 5.6 μm, *n* = 50) μm, with Q = 1.08–1.14, L = 6.24 µm, W = 5.67 µm (including myxosporium), overlaid by hyaline, dextrinoid, echinulae, echinulate brown inner wall, light yellow (4A4–4A5) to grayish-yellow (4B5–4B6) in 5% KOH. Basidia not seen.

*Habitat:* Solitary on decaying hardwood of *Machilus yunnanensis*, living tree of *Albizia mollis* and *Pinus* spp.

*Specimens examined:* CHINA, Yunnan Province, Kunming Institute of Botany garden, 25°08′39″ N, 102°44′30″ E, 1956 m elev., 31 December 2016, T. Luangharn, HKAS 97411.

*Notes: Ganoderma gibbosum* belongs to the family Ganodermataceae, which was first described in Australia [126]. *G. gibbosum* has been recorded from China [40], India [95], Korea [92], Laos [30], and Thailand [30]. This species is distinctive in having non-laccate basidiomes and ellipsoids with double-walled basidiospores [40]. *Ganoderma gibbosum* has been reported to cause white rot and several other diseases in hard woods [7] and is widely distributed in both tropical and temperate areas [4]. It was considered to be a subspecies of *G. applanatum* [4], while *G. applanatum* was the earlier name of *G. australe* [127]. *Ganoderma australe* and *G. gibbosum* were renamed as *G. incrassatum* based on their monophyletic origin [128] since it had been well recognized that *G. applanatum* was synonymized with *G. applanatum*.

**Figure 9:**
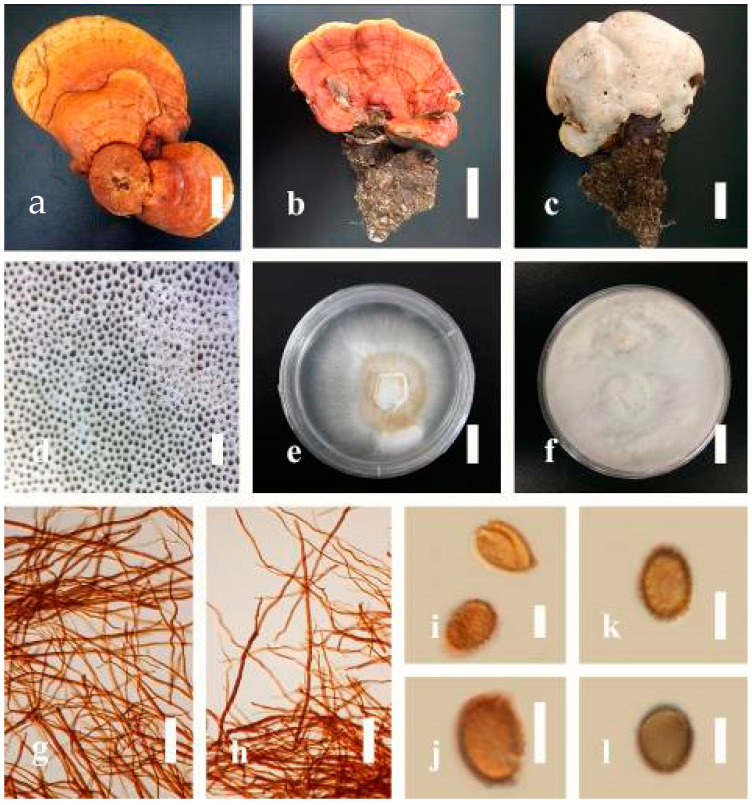
Morphology of *Ganoderma leucocontextum*: (**a**) mature basidiomes of the strain HKAS 97401; (**b**,**c**) mature basidiomes of the strain MFLU 19-2160; (**d**) pore characteristics; (**e**) culture after incubation at 25 °C for 14 days; (**f**) culture after incubation at 25 °C for 21 days; (**g**,**h**) context hyphae in KOH; (**i**–**l**) basidiospores. Scale bars: (**a**,**e**,**f**) = 2 cm, (**b**,**c**) = 4 cm, (**d**) = 500 µm, (**g**,**h**) = 30 µm, (**i**–**l**) = 5 µm.

***Ganoderma leucocontextum*** T.H. Li, W.Q. Deng, Sheng H. Wu, D.M. Wang and H.P. Hu, Mycotaxon 56: 82 (2015) (Figure 9)

Facesoffungi number: FoF 06247

*Description:* Basidiomes flabelliform, subdimidiate, stipitate. Pileus 6–14 cm in length, 4–12 cm in width, and 1–3.2 cm thick. Pileus flabelliform, spathulate, stipitate, subdimidiate to dimidiate, umbonate, somewhat semicircular, plump, concentrically sulcate zone, broad and thick at the base, mostly radial from the center extending to the margin, tough to break when dried, often thick at the center, slightly soft at the margin, light in weight when dried, and not woody. Pileus surface convex, furrowed, imbricate, incised, glossy, shiny, spathulate, shallow sulcate when fresh, umbonate or uneven, usually smooth layers at center when young to age, non-laccate to weakly laccate when present, strongly laccate and glossy when mature, weakly laccate where the new hyphae are in active development (margin), irregularly ruptured crust overlying the context, and tough to break when dried. Pileus color usually homogenous with orange (6A7) and deep orange (6A8) at the center toward stipe, extending deep orange (5A8) from the center, slight deep yellow (4A8) where the new hyphae are in active development when mature, usually red (11B7–11B8) at the center, and orange (6A7) to deep orange (6A8)–(6B8) extending to the upper margin surface from mature to old. Context up to 0.3–2.4 cm thick near stipe, white context when fresh, yellowish-white (1A2) when dried, soft and fibrous, trimitic hyphal, with clamp connections, hyaline, with walls varying in thickness with simple septa, and unbranched. Tubes 0.3–1.2 cm in length. Stipe 3–10 cm in length, 4–7 cm in width, sub-cylindrical to cylindrical, almost stipitate, broad at the base, some presented short stipitate, strongly laccate with dark brown (8F7–8F8) to grayish ruby (12E6–12E7) when mature, and grayish brown (8E4) when old. Margin wavy, softer, slippery when young, laccate when mature, strongly laccate when old, orange yellow (4A7) to deep yellow (4D8) where the new hyphae are in active development, deep orange (6A7–6A8) to brown (6D8) from mature to old. Pore 4–6 in number per mm, subcircular, some circular, or angular. Pore surface white (11A1) when present, yellowish-white (2A2) when mature, brownish-orange (6C7–6C8) when scratched or bruised, discolored when touched.

*Hyphal structure:* Hyphal system trimitic, usually golden brown (5D7), yellowish-brown (5D8) to reddish-brown (8D7–8D8) in KOH; generative hyphae 2.3–5.2 µm broad (*n* = 30), thin-walled, hyaline, with clamp connections; skeletal hyphae 2.6–5.5 µm broad (*n* = 30), thick-walled, unbranched or rearly branched; binding hyphae 1.8–4.2 µm width (*n* = 30), usually thin to thick-walled, branched, hymenial with sword-like apices in the context. Basidiospores mostly ellipsoid to broadly ellipsoid, double walls, (8.8-)9.3–*10.7*–11.4(-12.6) × (6.8-)7.4–*8.3*–8.7(-9.2) μm ($\bar{x}$ = 10.5 × 8.4 μm, *n* = 50) μm, with Q = 1.22–1.28, L = 10.52 µm, W = 8.41 µm (including myxosporium), (7.8-)8.1–*8.5*–8.8(-9.1) × (5.2-)5.7–*6.0*–6.5(-6.9) μm ($\bar{x}$ = 8.3 × 6.2 μm, *n* = 50) μm, with Q = 1.32–1.38, L = 8.34 µm, W = 6.18 µm (excluding outer myxosporium), overlaid by hyaline, apically echinulae, truncate, some turgid, vesicular appendix, inner walled echinulate, golden yellow (5B7), grayish-orange (5D6) to yellowish-brown (5D7), outer walled reddish-brown in 5% KOH. Cystidia absent. Cultures characteristics white mycelial after incubation at 30 °C for 10 days.

*Habitat:* Solitary, on the decaying hardwood of unknown tree.

*Specimens examined:* CHINA, Yunnan Province, Baoshan, 25°09′35″ N, 99°09′49″ E, 1973 m elev., 26 October 2016, J. Xu, HKAS 97401.

*Notes: Ganoderma leucocontextum* was introduced by Li et al. [62] from the Tibet Autonomous Region of China. This species can be easily recognized by its stipitate, white context, thick stipe, broadly ellipsoid basidiospores (9–12.5 × 7–9 μm), coarse echinulae, mostly regular cuticle hyphae, and its deciduous wood habitat [62]. The holotype is similar to *G. lucidum* from Europe [62]; however, the illustrated differences in macro-characteristics of the European *G. lucidum* are smaller basidiospores (7–12 × 6–8 μm), a deeper-colored context that is usually rust-colored, becoming dark purple to brown in older portions [105]. Additionally, *G. leucocontextum* also resembles the widely cultivated *G. lucidum* (*G. lingzhi*) in East Asia [62], but the Chinese *G. lucidum* has a deeper-colored context and is even darker near the tube layer, with shorter cutis elements (20–40 × 7–15 μm) and smaller spores (8–11.5 × 5.5–8.5 μm) (including myxosporium) than *G. leucocontextum leucocontextum* [5]. Our *G. leucocontextum* collection from Hainan Province agrees well with the descriptions provided by Li et al. [62].

**Figure 10:**
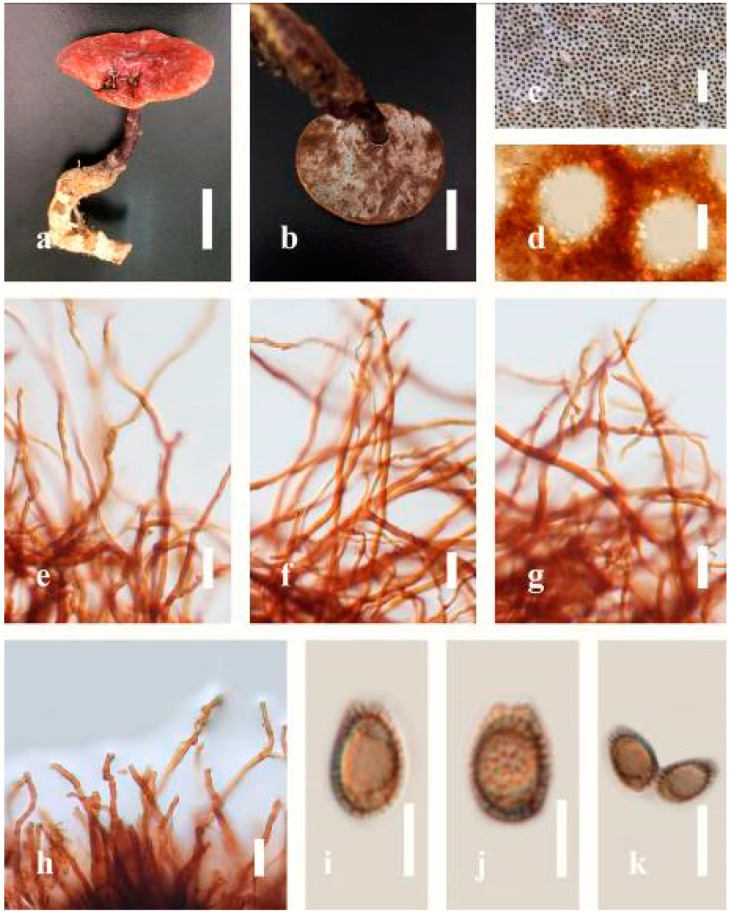
Morphology of *Ganoderma lucidum* (MFLU 19-2161): (**a**,**b**) mature basidiomes; (**c**) pore characteristics; (**d**) hyphae of pore characteristics in KOH; (**e**) hyphae of trama in KOH; (**f**,**g**) generative and skeletal hyphae of context in KOH; (**h**) hyphae of tube layers in KOH; (**i**–**k**) basidiospores. Scale bars: (**a**,**b**) = 2 cm, (**c**) = 1000 µm, (**d**) = 150 µm, (**e**–**g**) = 20 µm, (**h**) = 15 µm, (**i**–**k**) = 5 µm.

***Ganoderma lucidum*** (Curtis) P. Karst., Revue Mycologique Toulouse. 3(9): 17 (1881) (Figure 10)

≡ *Boletus rugosus* Jacq., Flora Austriaca. 2: 44, f. 169 (1774)

≡ *Boletus lucidus* Curtis, Fl. Londinensis. 4: 72, t. 224 (1781)

≡ *Polyporus lucidus* (Curtis) Fr., Systema Mycologicum. 1: 353 (1821)

≡ *Grifola lucida* (Curtis) Gray, A natural arrangement of British plants. 1: 644 (1821)

≡ *Fomes lucidus* (Curtis) Cooke, Grevillea. 13(68): 118 (1885)

≡ *Placodes lucidus* (Curtis) Quél., Enchiridion Fungorum in Europa media et praesertim in Gallia Vigentium.: 170 (1886)

≡ *Phaeoporus lucidus* (Curtis) J. Schröt., Kryptogamen-Flora von Schlesien. 3-1(4): 491 (1888)

= *Boletus flabelliformis* Leyss., Flora halensis.: 219 (1761)

= *Agaricus pseudoboletus* Jacq., Miscellanea austriaca ad botanicum, chemiam et historiam naturalem spectantia. 1: 26, t. 41 (1773)

= *Boletus obliquatus* Bull., Herbier de la France. 1: t. 7 (1781)

= *Boletus vernicosus* Bergeret, Phytonomatotechnie universelle. 1: 99 (1783)

= *Agaricus lignosus* Lam., Encyclopédie Méthodique, Botanique. 1-1: 51 (1783)

= *Boletus dimidiatus* Thunb., Fl. Japonica.: 348, f. 39 (1784)

= *Boletus castaneus* Weber, Suppl. Fl. hols.: 13 (1787)

= *Boletus laccatus* Timm, Flora megapolitanae Prodomus exhibeus plantas ductatus Megapolitano.: 269 (1788)

= *Boletus crustatus* J.J. Planer, Index Plantarum quas in Agro Erfurtensi sponte provenientes.: 280 (1788)

= *Agarico igniarium* trulla Paulet, Traité des champignons. 2: 95, pl. 10:1-2 (1793)

= *Boletus verniceus* Brot., Flora Lusitanica. 2: 468 (1804)

= *Ganoderma ostreatum* Lázaro Ibiza, Revta R. Acad. Cienc. exact. fis. nat. Madr.: 110(1916)

= *Ganoderma nitens* Lázaro Ibiza, Revta R. Acad. Cienc. exact. fis. nat. Madr.: 104 (1916)

Facesoffungi number: FoF 06250

*Description:* Basidiomes imbricate, reniform, stipitate. Pileus up to 2–5 cm in length, 2–4 cm in width, and 0.8–2.2 cm thick. Pileus stipitate, reniform, imbricate, irregular, some laterally, and flabelliform with a contracted, concentrically sulcate zone, irregularly ruptured crust overlying the context, radial or branched from the center extending to the margin, tough to break when dried, often thick at the center, slightly soft at margin, and leathery when aged, tough to break when dried. Pileus surface weakly laccate when present, strongly laccate and glossy when mature, weakly laccate where the new hyphae are in active development (margin), smooth layer at the center from young to age, usually furrowed, incised, undulate to sulcate, somewhat spathulate to uneven, some woody or corky when old. Pileus color usually yellowish-red (8B7–8B8) at the center, slight to reddish-orange (7B7–7B8), and orange (6A7–6A8) on upper pileus surface. Context up to 0.4–1.4 cm thick at the base, abundant thick-walled, subsolid hyphae, concentric lines of various shade, bearing clamp connections, light brown (6D6) to brown (6D8, 6E8), presenting dark brown (6F8) melanoid bands. Tube hard, often brown (7D7) to dark brown (7F7). Stipe up to 8–16 cm in length, up to 0.6–1.8 cm in width, central stipe, cylindrical, thick with uneven at the base (up to 1.8 cm), usually dark brown (7F7–7F8), laccate, and cracked when old. Margin often 0.4–1 cm, orange (6A7–6A8) on upper surface, and reddish-yellow (4A8) under surface, thin and soft than the center. Pore (75–)110–145(–165) μm, circular, some angular, 4–6 in number per mm. Pore surface white (11A1) to light brown (7D6), turning brown (7D7–7D8) to dark brown (6F6) when scratched or bruised.

*Hyphal structure:* Hyphal system trimitic, with clamp connections, hyaline, thin-walled with abundant thick-walled with simple septa, sparingly branched, swollen by melanoid bands, usually pale orange (5A3), light orange (5A5), to reddish-orange (8A7–8A8) in KOH; generative hyphae up to 1.7–3.2 μm broad (*n* = 30), almost hyaline, usually thin to thick-walled, with clamp connections, and sparingly branched and flexuous; skeletal hyphae 3.0–6.4 μm broad (*n* = 30), usually thick-walled with clamp, and abundantly branched and flexuous; binding hyphae 2.0–5.6 μm broad (*n* = 30), usually thick-walled with abundant branches, and occurring melanoid bands. Basidiospores ellipsoid to broadly ellipsoid, some globose with double walls, with a truncate apex, with double wall, mostly overlaid by hyaline myxosporium, eusporium bearing fine, short, and distinct, coarse, echinulae, hyaline, turgid, vesicular appendix, (7.7-)8.4–*9.4*–10.6(-11.5) × (5.2-)5.9–*6.3*–7.1(-8.4) μm, ($\bar{x}$ = 9.5 × 6.4 μm, *n* = 50) μm, with Q = 1.47–1.52, L = 9.52 µm, W = 6.34 µm (including myxosporium), (6.0-)6.9–*7.3*–8.1(-8.5) × (4.6-)4.9–*5.3*–5.8(-6.2) μm ($\bar{x}$ = 7.5 × 5.2 μm, *n* = 50) μm, with Q = 1.41–1.47, L = 7.52 µm, W = 5.24 µm (excluding outer myxosporium), brownish-orange (6C8), (6D8) to brown (6E5) of endosporium (inner wall) with brown (7E7–7E8) exosporium (outer wall) in Congo red, brownish-orange (6C8) in 5% KOH, and yellowish-brown in Melzer’s reagent.

*Habitat:* Solitary, on decaying hardwood of *Quercus* sp. in the native forest.

*Specimens examined:* CHINA, Yunnan Province, Honghe, 23°21′50″ N, 103°22′24″ E, 874 m elev., 15 August 2017, T. Luangharn, MFLU 19-2161.

*Notes: Ganoderma lucidum* (Curtis) P. Karst. was originally reported from temperate England [2]. Previously, it was characterized as *Boletus lucidus* Curtis and then *Polyporus lucidus* (Curtis) Fr. (1821) [1]. The species *P. lucidus* was characterized by having a laccate pileus and stipe. The molecular phylogenetic analyses indicated that the *G. lucidum* from Europe is not conspecific to the Chinese *G. lucidum*; thus, the European *G. lucidum* remained the true *G. lucidum,* and the Chinese *G. lucidum* was proposed as *G. lingzhi* [4], and most of the collections named *G. lucidum* in East Asia were not conspecific with the *G. lucidum* found in Europe [129]. *Ganoderma* *lucidum* is relatively common in Europe, while its geographic distribution in East Asia, East Africa, Europe, North America, Asia, and other parts of the world is largely unknown [38].

Several studies of *Ganoderma* have used the name *G. lucidum* for any laccate *Ganoderma* species, as *Ganoderma* are ly highly variable, often resulting in taxonomic and phylogenetic confusion, especially with regards to *G. lucidum* [38]. The taxonomy of *Ganoderma* has been a constant topic of debate due to the high levels of phenotypic plasticity in its species. Several characteristics of *Ganoderma* are similar to *G. lucidum*, such as *G. multipileum* [39], *G. oregonense* [42], *G. resinaceum* [41], *G. tsugae*, *G. lucidum*, *G. sichuanense*, and *G. sinense* [4,32,33,35,40,130] from China. Cao et al. [4] have clarified a different new species, Chinese *G. lucidum* as *G. lingzhi*, which has an East Asian distribution. The most striking characteristics that differentiate *G. lucidum* from *G. lingzhi* are the presence of melanoid bands in the context, a yellow pore surface, and thick dissepiments (80–120 μm) at maturity [4]. The molecular evidence reveals *G. lucidum* and *G. sinense* as two clear different species [130].

In China, the first-reported *G. lucidum* was illustrated based on collections from Guizhou Province [124]. Then, Teng [131] reported more collections from different regions of China, and many subsequent collections have been reported [4,13,47,121,132]. Recently, this fungus has been reported to be distributed worldwide based on gross similarity of features, e.g., in Europe [105], Asia [13,133], America [134,135], and Africa [112]. Our collection from Yunnan Province, China, also agrees well with the descriptions provided from Asia.

**Figure 11:**
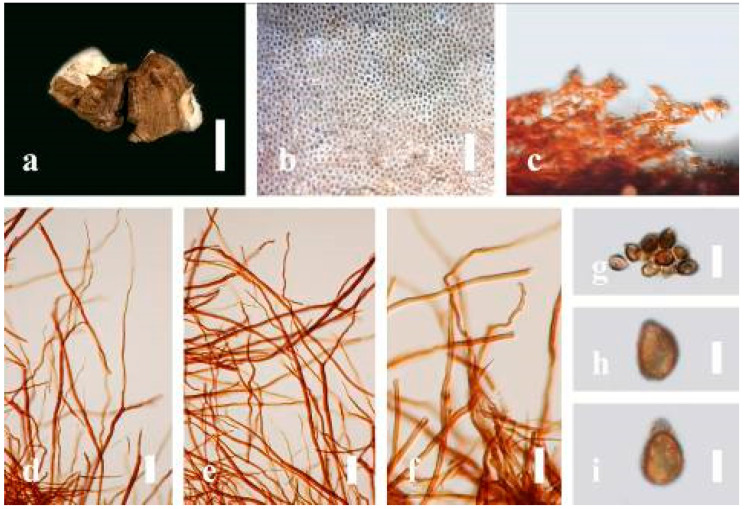
Morphology of *Ganoderma multiplicatum* (MFLU 19-2152): (**a**) mature basidiomes; (**b**) pore characteristics; (**c**) hyphae of tube layers in KOH; (**d**–**f**) context hyphae in KOH; (**g**–**i**) basidiospores. Scale bars: (**a**) = 2 cm, (**b**) = 1000 µm, (**d**–**f**) = 20 µm, (**g**–**i**) = 5 µm.

***Ganoderma multiplicatum*** (Mont.) Pat., Bulletin de la Société Mycologique de France 5: 74 (1889) (Figure 11)

≡ *Polyporus multiplicatus* Mont., Annales des Sciences Naturelles Botanique. 1: 128 (1854)

≡ *Fomes multiplicatus* (Mont.) Cooke, Grevillea. 14 (69): 18 (1885)

≡ *Scindalma multiplicatum* (Mont.) Kuntze, Revisio generum plantarum. 3 (2): 519 (1898)

Facesoffungi number: FoF 06251

*Description:* Basidiomes annual or perennial, stipitate with short base. Pileus 1.5–7.5 cm in length, 0.5–4 cm in width, and up to 1.5 cm thick at the base. Pileus dimidiate, flabelliform, reniform, usually flat, convex, imbricate, umbonate or uneven, rarely ungulate, glabrous when present, often with undefined concentric zones at the center that extend to the margin, and thick at the base, slightly soft at margin when mature. Pileus surface shiny, silky, smooth, and soft when young, non-laccate (dull) when mature, hard and woody when old, frequently furrowed and shallow sulcate, undulating, somewhat spathulate to uneven on upper surface when mature, covered by irregularly ruptured thick crust, slightly dull and faded when mature to old, compact and hard when mature, woody to corky from mature to old. Pileus color usually homogenous with grayish-orange (6B3) at the center slight to brownish-orange (6C4) and pale orange (6A3), usually yellowish-gray (4B2) at the margin when mature, and brown (6E8) when dried. Context up to 0.4–1 cm thick at the base, mostly brown (6E8) to dark brown (7F6–7F8) of cuticle cells, and dark brown (6F6) melanoid bands, thick-walled, some fibrous pithy context, usually separated by layers of context tissue at the base. Tube woody hard, often with dark brown (7F7–7F8) when dried, with sulcate at different levels. Stipe short stipitate, dark brown (7F7), and a differentiated zone at the point of attachment. Margin up to 1 cm thick, initially white (5A1), yellowish-gray (4B2) when mature, turns light brown (6D4) to brown (6E8) when scratched or bruised, often slippery when wet, softer when young, thinner than the center. Pore 4–7 in number per mm, subcircular to circular, some angular. Pore surface initially white (7A1) to yellowish-white (1A2), becoming pale orange (5A3) when mature, light brown (7D6) to brown (7D8) when handled, scratched, and bruised.

*Hyphal structure:* Hyphal system trimitic; generative hyphae 2.1–4.8 µm ($\bar{x}$ = 2.2, *n* = 30) in diam, clamp, almost hyaline, thin to thick-walled, composed of narrow and spare branches; skeletal hyphae 3.2–6.5 µm width (*n* = 30), usually thick-walled, hyaline, some branched and intertwined hyphae; binding hyphal 2.4–5.7 µm width (*n* = 30), thick-walled, many branches, and comprised Bovista-type ligative hyphae. Pileipellis a hymeniderm, brown (6E8), composed of apically acanthus-like branched cells, dextrinoid. Basidiospores mostly ellipsoid with double walls, (7.8-)8.7–*10.8*–12.2(-13.3) × (6.9-)7.4–*9.1*–10.0(-10.7) μm, ($\bar{x}$ = 10.7 × 9.1 μm, *n* = 50) μm, with Q = 1.15–1.22, L = 10.79 µm, W = 9.13 µm (including myxosporium), (5.4-)5.9–*6.6*–7.1(-7.7) × (4.9-)5.4–*5.8*–6.2(-6.7) μm ($\bar{x}$ = 6.6 × 5.8 μm, *n* = 50) μm, with Q = 1.11–1.17, L = 6.64 µm, W = 5.82 µm (excluding outer myxosporium), inner walled deep orange (6A8), brownish-orange (7C7–7C8) in KOH, outer walled dark brown (7E6), dark brown (7E8) to reddish-brown (8E8) in KOH. Cutis usually composed of clavate cells.

*Habitat:* Solitary on stump of *Quercus* spp.

*Specimens examined:* CHINA, Yunnan Province, Jinning District, 24°41′17″ N, 102°13′15″ E, 1912 m elev., 8 October 2017, T. Luangharn, MFLU 19-2152.

*Notes: Ganoderma multiplicatum* was originally collected from French Guyana [2]. This species has a distinctive form with its reddish-black pileus, a not fully homogenous context, tuberculate hyphal ends in cuticle cells, with small subglobose to broadly ellipsoid basidiospores (7–8 × 5–6 μm) [7,136,137,138]. *Ganoderma multiplicatum* has been considered most similar to *G. chalceum* [113], also considered a synonym of *G. subamboinense* Henn. [136], but Correia de Lima et al. [139] illustrated that *G. chalceum* and *G. subamboinense* are in different clades, suggesting they are not synonymous. Our *G. multiplicatum* specimen was collected from Yunnan Province, China. It is similar to the original description, showing ellipsoid basidiospores, while sub-globous basidiospores could not be observed. This species has been reported from Africa [140], Asia [61], China [13,61,119,122], India [141], Myanmar [30], Taiwan, PRC [122], and neotropical regions of Brazil, Colombia, and Venezuela [138].

**Figure 12:**
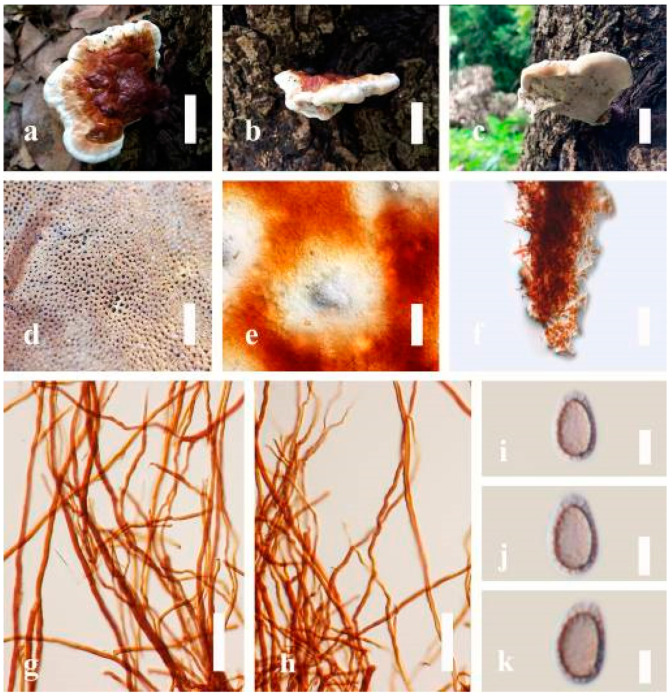
Morphology of *Ganoderma resinaceum* (MFLU 19-2153): (**a**–**c**) mature basidiomes; (**d**) pore characteristics; (**e**) hyphae of pore in KOH; (**f**) hyphae of tube layers in KOH; (**g**,**h**) context hyphae in KOH; (**i**) basidiospores in Melzer’s reagent; (**j**,**k**) basidiospores in KOH. Scale bars: (**a**–**c**) = 4 cm, (**d**) = 1000 µm, (**e**) = 150 µm, (**f**) = 30 µm, (**g**,**h**) = 50 µm, (**i**–**k**) = 5 µm.

***Ganoderma resinaceum*** Boud., Bulletin de la Société Mycologique de France. 5; 72 (1889) (Figure 12)

≡ *Fomes resinaceus* (Boud.) Sacc., Sylloge Fungorum. 9: 179 (1891)

≡ *Scindalma resinaceum* (Boud.) Kuntze, Revisio generum plantarum. 3(2); 519 (1898)

≡ *Friesia resinacea* (Boud.) Lázaro Ibiza, Revta R. Acad. Cienc. exact. fis. nat. Madr.: 591 (1916)

≡ *Ganoderma lucidum* subsp. *resinaceum* (Boud.) Bourdot and Galzin, Bulletin de la Sociètè Mycologique de France. 41; 177 (1925)

≡ *Ganoderma lucidum* var. *resinaceum* (Boud.) Maire, Fungi Catalaunici: Contributions á lètude de la Flore Mycologique de la Catalogne: 38 (1933)

= *Ganoderma chaffangeonii* Pat., Bulletin de la Société Mycologique de France. 5: 74 (1889)

= *Polyporus polychromus* Copel., Annales Mycologici. 2 (6): 507 (1904)

= *Ganoderma praelongum* Murrill, North American Flora. 9 (2): 121 (1908)

= *Ganoderma argillaceum* Murrill, North American Flora. 9 (2): 122 (1908)

= *Ganoderma pulverulentum* Murrill, North American Flora. 9 (2): 121 (1908)

= *Ganoderma subperforatum* G.F. Atk., Botanical Gazette Crawfordsville. 46 (5): 337 (1908)

= *Ganoderma areolatum* Murrill, Bulletin of the New York Botanical Garden. 8: 149 (1912)

= *Mensularia vernicosa* Lázaro Ibiza, Revista de la Real Academia de Ciencias Exactas Fisicas y Naturales Madri. 14: 740 (1916)

= *Ganoderma subtuberculosum* Murrill, Lloydia. 7 (4): 326 (1945)

Facesoffungi number: FoF 06252

*Description:* Basidiomes annual, perennial, short stipitate. Pileus 1.5–12.5 cm in length, 1–7 cm in width, and up to 2 cm thick at the base. Pileus dimidiate, flabelliform, reniform, convex, imbricate, umbonate or uneven, some ungulate, concentric zones at the center that extend to the margin, broadly attached, thick at the base, slightly soft at the margin when mature. Pileus surface glossy, shiny, silky, smooth, and soft when young, laccate when mature, furrowed and shallow sulcate, undulating, somewhat spathulate to uneven on upper surface when mature, covered by irregularly ruptured thin crust, slightly dull and faded when mature to old, compact and hard when mature, woody to corky when mature to old. Pileus color reddish-brown (10E7–10E8) at the center, slight to yellowish-red (8B7–8B8), reddish-orange (7A7–7A8), and light orange (5A5–5A6) closed to the margin, and white (4A1) at the margin. Context up to 0.4–1 cm thick at the base, mostly grayish-yellow (4C6) to dark brown (7F6–7F8) cuticle cells, and dark brown (6F6) melanoid bands, thick-walled, some fibrous pithy context, usually separated by layers of context tissue at the base. Tube woody hard, often dark brown (7F7–7F8) when dried, concolorous with pore surface, and sulcate at different levels. Stipe short stipitate, usually reddish-brown (10E7–10E8), and a differentiated zone at the point of attachment. Margin up to 1.5 cm thick, initially white (5A1), yellowish-gray (4B2) when mature, turning light brown (6D4) to brown (6E8) when scratched or bruised, often slippery when wet, softer when young, thinner than the center. Pore 4–7 in number per mm, angular to circular. Pore surface initially white (7A1) to yellowish-white (1A2), becoming light orange (5A5) when mature, light brown (7D6) to brown (7D8) when handled, scratched, or bruised.

*Hyphal structure:* Hyphal system trimitic; generative hyphae 2.1–4.7 µm ($\bar{x}$ = 3.6, *n* = 30) in diam, clamp, almost hyaline, thin-walled, composed of sparse branches; skeletal hyphae 3.2–6.2 µm width (*n* = 30), usually thick-walled, hyaline, some branched and intertwined hyphae; binding hyphal 2.8–5.1 µm width (*n* = 30), thick-walled and occasionally thick-walled, without septate hyphae, many branches, and composed of Bovista-type ligative hyphae. Basidiospores mostly ellipsoid with double walls, (7.6-)8.4–*9.4*–10.5(-11.3) × (6.5-)7.1–*8.4*–9.0(-9.8) μm, ($\bar{x}$ = 9.3 × 8.2 μm, *n* = 50) μm, with Q = 1.10–1.16, L = 9.31 µm, W = 8.24 µm (including myxosporium), (6.5-)7.1–*8.2*–9.1(-9.8) × (4.8-)5.3–*5.7*–6.8(-7.3) μm ($\bar{x}$ = 8.1 × 5.6 μm, *n* = 50) μm, with Q = 1.42–1.48, L = 8.13 µm, W = 5.62 µm (excluding outer myxosporium), inner walled orange (5A6) to deep orange (5A7–5A8, 6A8) in KOH and grayish brown (5C5–5C6) in Melzer’s reagent, outer walled dark brown (7E6–7E8) to reddish-brown (8E8) in KOH and light brown (6D5–6D6) to brown (6D7–6D8) in Melzer’s reagent.

*Habitat:* Solitary, on living tree of *Albizia mollis* (Wall.) Boiv.

*Specimens examined:* CHINA, Yunnan Province, Kunming Institute of Botany, 25°08′39″ N, 102°44′30″ E, 1962 m, 12 July 2017, T. Luangharn, MFLU 19-2153.

*Notes: Ganoderma resinaceum* was introduced by Boudier in 1889 from France [41]. This species has also been described by Steyaert [110] and Ryvarden and Gilbertson [105]. *Ganoderma* *resinaceum* is distinctively characterized by variable pileus coloration, a fibrous spongy homogeneous context, larger basidiospores, and an amyloid pileipellis [7]. This species is considered to have characteristics similar to *G. pfeifferi* in its upper crust resinous layers. However, this species has a dark brown to umber context and wider spores. In addition, *G. resinaceum* also shares similarities with *G. lucidum*, while *G. lucidum* possesses a varying light context without a dark zone above the tubes and no resinous layer on the crust [105,140]. *Ganoderma resinaceum* was evaluated to the species complex base on molecular evidence [38], but in the phylogenetic analysis, it cannot be distinguished from *G. lucidum* [142]. However, several researchers suggested that *G. resinaceum* differs from *G. lucidum* [4,76,143].

***Ganoderma sanduense*** Hapuar., T.C. Wen and K.D. Hyde, Mycosphere 10, 274 (2019)

Taxonomy and phylogenetic analyses are shown in Hapuarachchi et al. [30].

Notes: *Ganoderma sanduense* is characterized by its ferruginous laccate pileus, orbicular, strongly laccate, several layers thick, basidiospores 12.1–13.8 × 9.2–10.5 μm, relatively large broadly ellipsoid to ellipsoid basidiospores, with a light brown eusporium bearing fine, hyaline, short, and distinct echinulae. This fungus is solitary on rotten wood in dry dipterocarp forests and in upper-mixed deciduous forests from Guizhou Province, China.

**Figure 13:**
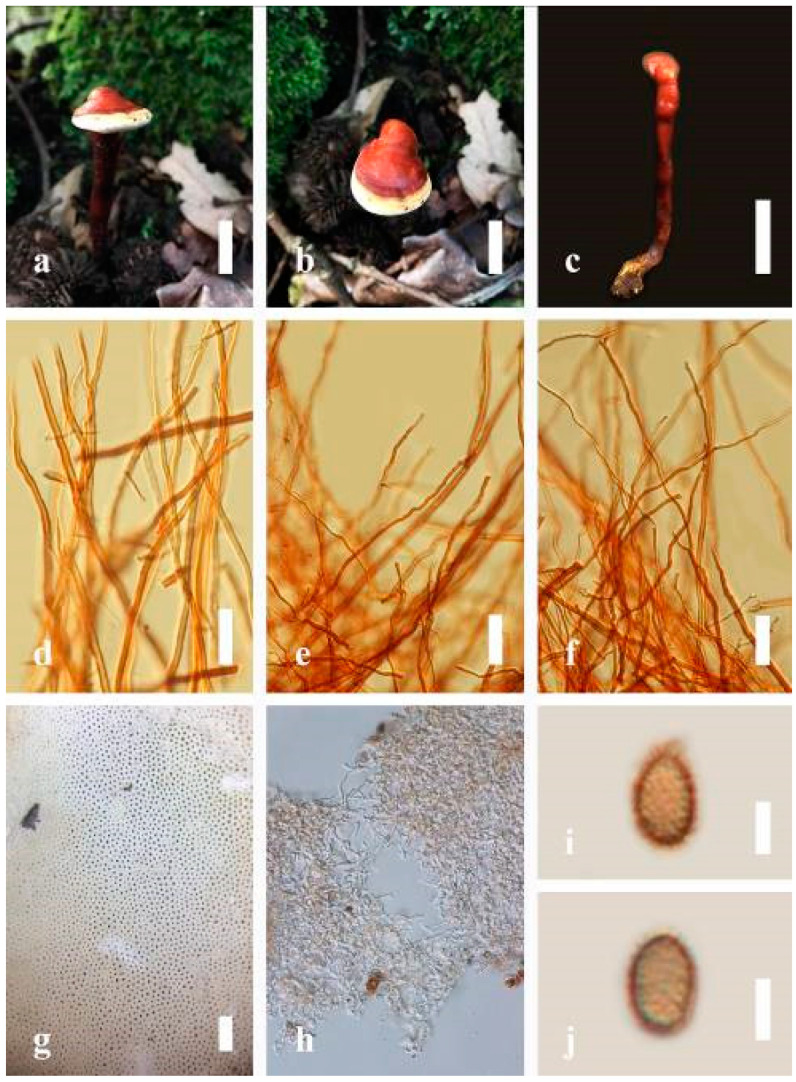
Morphology of *Ganoderma sichuanense* (HKAS 97398): (**a**–**c**) mature basidiomes; (**d**) hyphae of trama in KOH; (**e**,**f**) context hyphae in KOH; (**g**) pore characteristics; (**h**) hyphae of pore in KOH; (**i**,**j**) basidiospores. Scale bars: (**a**,**b**) = 1 cm, (**c**) = 3 cm, (**d**) = 20 µm, (**e**,**f**) = 30 cm, (**g**) = 1000 µm, (**i**,**j**) = 5 µm.

***Ganoderma sichuanense*** J.D. Zhao and X.Q. Zhang, Acta mycol. sin.: 159 (1983) (Figure 13)

Facesoffungi number: FoF 06248

*Description:* Basidiomes annual or perennial, stipitate. Pileus 0.5–3.2 cm in length, 0.5–3 cm in width, up to 1 cm thick at the base. Pileus reniform to circular, or subflabellate when seen from above, concentrically sulcate zones with turberculate, glabrous when youth to maturity, bumps when mature, often tough to break when dried, often with undefined concentric zones at the center that extend to the margin, thick at the center, slightly soft at the margin. Pileus surface shiny, silky, smooth, and soft when young, hard and woody old, frequently furrowed and shallow sulcate on upper surface, undulating, somewhat spathulate to uneven when mature, covered by irregularly ruptured thin crust, and strongly laccate from mature to old. Pileus color usually homogenous with yellowish-red (8A7–8A8) at the center, slight reddish-orange (7A7–7A8), and reddish-brown (8E8) at the deep-sulcate margin from mature to old. Context up to 0.2–0.8 cm thick at the base, some thin-walled, with abundant thick-walled to subsolid hyphae, containing fibrous pithy context, bearing clamp connections, with dark brown (7F7) melanoid bands occurring. Tube hard and woody, thin-walled, frequently branched, with clamped connection, and often dark brown (7F7–7F8) when dried. Stipe up to 3–812 cm in length, up to 0.3–1 cm in width, centrally stipitate, almost sub-cylindrical to cylindrical, concolorous with the pileus, often reddish-brown (8E7–8E8), and strongly laccate from mature to old. Margin soft when young, strongly laccate when mature, some wavy, slippery when wet, smooth, softer, thinner than the base, and soft than the center, often deep orange (5A8) to golden yellow (5B7–5B8) from mature to old. Pore 4–6 in number per mm, subcircular to circular, sometimes angular. Pore surface initially white (11A1), pale yellow (3A3) to yellow (3A7) when mature, turns light brown (7D5), brown (7D7–7D8) to dark brown (7F6–7F8) when scratched or bruised, becoming discolored when touched.

*Hyphal structure:* Hyphal system trimitic, with clamp connections, hyaline, thin to thick-walled, tapering at branch, sometimes swollen at the attachment point, composed of some narrow hyphae; generative hyphae (1.3–)1.8–*2.3*–2.6(–2.8) μm broad (*n* = 30), hyaline, and thin-walled; skeletal hyphae (2.1–)2.5–*3.9*–4.8(–5.2) μm broad (*n* = 30) abundant with walls varying in thickness, unbranched, sometimes subsolid; binding hyphae (1.7–)2.1–*2.8*–3.6(–4.3) μm broad (*n* = 30), usually with walls varying in thickness, narrow to subsolid, usually presenting as orange white (6A2), pale orange (6A3) to light orange (5A5) of thin-walled, and pale red (6A3) of thick-walled, with subsolid in KOH. Pileipellis a hymeniderm, light brown (6D6), clavate-like cells, dextrinoid. Basidiospores mostly ellipsoid, some oblong with double walls, (8.0-)8.6–9.6–10.5(-11.0) × (6.2-)6.7–8.4–9.6(-10.1) μm ($\bar{x}$ = 9.5 × 8.3 μm, *n* = 50) μm, Q = 1.11–1.17, L = 9.49 µm, W = 8.31 µm (including myxosporium), (7.0-)7.4–7.9–8.5(-9.0) × (4.2-)4.5–5.6–5.9(-6.4) μm ($\bar{x}$ = 7.8 × 5.6 μm, *n* = 50) μm, with Q = 1.36–1.41, L = 7.80 µm, W = 5.62 µm (excluding outer myxosporium), overlaid by hyaline, dextrinoid, echinulae, inner wall echinulate with grayish-orange (6B5–6B6) to brownish-orange (7D4–7D5), and outer walled usually dark brown (7E8–7E8) to reddish-brown (8E6–8E8) in KOH.

*Habitat:* Solitary on the living tree of *Graucoides schotky*.

*Specimens examined:* CHINA, Yunnan Province, Xishan Forest Park, 24°57′53″ N, 102°53′10″ E, 2013 m elev., 29 October 2016, T. Luangharn, HKAS 97398.

*Notes: Ganoderma sichuanense* was originally described from the Sichuan Province, China, in 1983 [5]. However, *G. sichuanense* was published in 1983 [40] but has not been widely used. *Ganoderma sichuanense* was verified as “*G. lucidum*” (Lingzhi) based on both morphological and molecular data. This fungus was distinguished from other *Ganoderma* species. *Ganoderma sichuanense* was characterized by its distinctive substipitate to stipitate, flabellate to reniform, radially rugose pileus, laccate with a verrucose or tuberculose upper surface, pore surface yellowish when young, becoming brown or black when bruised, and small spores [13]. Originally the basidiospores were described as (7.4–9.5 × 5–7) µm [40], then updated to (7.8–10.4 × 5.2–6.4) µm [13,61], and (9–11.5 × 6.5–8) µm [5]. The study basidiospores were 7.8–9.5 × 5.6–8.3 μm, which is in the range of the original report, which is not distinct from those of basidiospores found in other reports. Cao et al. [4] stated that *G. sichuanense* differs from *G. lingzhi* as its sessile basidiocarps and smaller basidiospores (7.4–9.2 × 5–6.6) µm, with distinctive yellow context, thick dissepiments, absence of concentric growth zones in the context, basidiospore size, yellow pore surface, and presence of melanoid bands upon maturity [4,37]. *Ganoderma sichuanense* was yellowish-brown, with a dark brown eusporium bearing thick echinulae, overlaid by a hyaline myxosporium. However, among the Chinese *Ganoderma* species, *G. flexipes*, *G. multipileum*, *G. sichuanense*, *G. tropicum,* and *G. tsugae* are the most similar species to *G. lingzhi* because they share a reddish-brown pileal surface, similar basidiospores, and cuticle cells [4].

**Figure 14:**
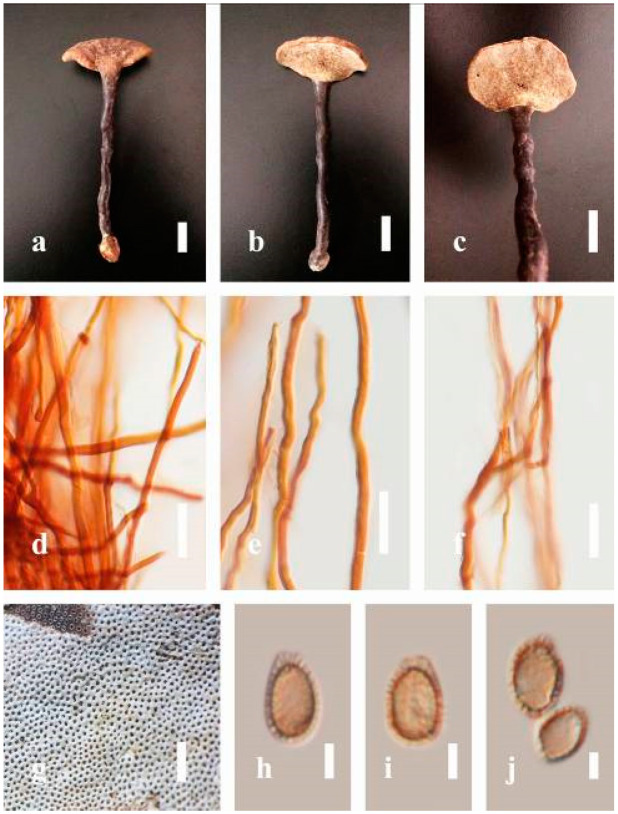
Morphology of *Ganoderma sinense* (MFLU 19-2173): (**a**–**c**) mature basidiomes; (**d**–**f**) context hyphae in KOH; (**g**) pore characteristics; (**h**–**j**) basidiospores in KOH. Scale bars: (**a**–**c**) = 2 cm, (**d**–**f**) = 20 µm, (**g**) = 1000 µm, (**h**–**j**) = 5 µm.

***Ganoderma sinense*** J.D. Zhao, L.W. Hsu and X.Q. Zhang, Acta Mycologica Sinica. 19: 272 (1979) (Figure 14)

= *Ganoderma formosanum* T.T. Chang and T. Chen. Transactions of the British Mycological Society. 82(4): 731 (1984)

Facesoffungi number: FoF 06253

*Description:* Basidiomes annual, stipitate, subdimidiate. Pileus 2–6 cm in length, 2–4 cm in width, and 0.3–1 cm thick. Pileus stipitate, subdimidiate to dimidiate, flabelliform, spathulate, umbonate, radial from the center extending to the margin, tough to break when dried, often thick at the center, slightly soft at the margin, light in weight when dried, and without woody. Pileus surface laccate, convex, some radial furrowed to furrowed, imbricate, incised, glossy, shiny, spathulate, shallow sulcate when fresh, umbonate or uneven, strongly laccate and glossy when mature, and weakly laccate where the new hyphae are in active development (margin), usually smooth layers at the center when young to age, irregularly ruptured crust overlying the context, and leathery when age when break. Pileus color usually homogenous with brownish-red (8C7–8C8) to reddish-brown (8D7–8D8) at the center toward stipe, extending brownish-red (9C8) from the center, slight to the margin when mature, usually reddish-brown (8E5–8E8) upper margin surface when old. Context up to 0.3–1 cm thick near stipe, dry, upper layer brownish-orange (6C8) when fresh, grayish-orange (5B5) at lower layers, with dark brown (8F7) when dried, soft and fibrous, covered with thin crust, some present woody, trimitic hyphal, hyaline, thin to thick-walled with simple septa, with branched. Tube 0.3–0.6 cm in length, brown (7D8). Stipe 4–16 cm in length, sub-cylindrical to cylindrical, almost stipitate with broadly and thick at the base, irregularly ruptured crust overlying, usually strongly laccate with brown (7D8) to dark brown (8F8) when mature, and dark brown (8F8) when old. Margin soft, some wavy, laccate when mature, weakly laccate when old, brownish-orange (6D8) when mature to old. Pore 4–6 in number per mm, subcircular to circular. Pore surface white (11A1) to yellowish-white (2A2) when mature, discolored when touched, brownish (6E7) to dark brown (6F7) when scratched or bruised.

*Hyphal structure:* Hyphal system trimitic, with clamp connections, usually light orange (5A5), orange (5A7), golden yellow (5B7–5B8), sometimes brownish-red (8C7) in KOH; generative hyphae 1.3–2.4 µm broad (*n* = 30), hyaline, thin-walled, with clamp connections; skeletal hyphae 3.1–5.2 µm broad (*n* = 30), usually hyaline, thick-walled, unbranched, and solid; binding hyphae 2.9–5.2 µm width (*n* = 30), thin to thick-walled, with branched, hymenial with sword-like apices in the context. Basidiospores mostly ellipsoid to broadly ellipsoid, with double walls, with size range of (9.5-)10.2–11.4–12.3(-13.1) × (7.0-)7.6–8.4–9.3(-10.2) μm ($\bar{x}$ = 11.2 × 8.5 μm, *n* = 50) μm, with Q = 1.28–1.36, L = 11.24 µm, W = 8.50 µm (including myxosporium), (8.3-)9.4–10.3–11.5(-12.3) × (6.1-)6.7–7.1–7.5(-8.0) μm ($\bar{x}$ = 10.2 × 7.2 μm, *n* = 50) μm, with Q = 1.41–1.47, L = 10.32 µm, W = 7.13 µm (excluding outer myxosporium), overlaid by hyaline, apically, short echinulae, truncate, some turgid vesicular appendix, inner wall echinulate, orange (5A7), deep orange (5A8, 5B8), orange (6B8), with brownish-orange (6B8), outer wall usually brownish-red (8C7–8C8) in 5% KOH.

*Habitat:* Solitary on decaying and living tree of *Albizia mollis* (Wall.) Boiv., living tree *Quercus* sp.

Specimens examined: CHINA, Yunnan Province, Baoshan, 25°09′35″ N, 99°09′49″ E, 1973 m elev., 11 November 2017, T. Luangharn, MLFU 19-2173.

*Notes: Ganoderma sinense* was described from China, characterized by a uniformly brown to dark brown context and slightly longitudinally crested basidiospores [122]. This species was considered as a species with high phenotypic [13,36]. Our *G. sinense* collection was obtained from Yunnan Province, China, and agrees well with the description of the holotype as described by Wang and Wu [122]. Several reports have also illustrated *G. sinense* from China [13,47,123]. *Ganoderma sinense* is considered to have characteristics similar to *G. lucidum*, while *G. sinense* illustrates differences in macro- characteristics in its thin pileus, long stipes, and rarely branched skeletal hyphae with Bovista-type binding hyphae [106], and these two different species are distinguished in reports [130]. *Ganoderma sinense* is also reported to have similar characteristics with *G. formosanum*; hence, *G. formosanum* was treated as synonymous, and consequently, the earliest used valid name was *G. sinense* [122].

**Figure 15:**
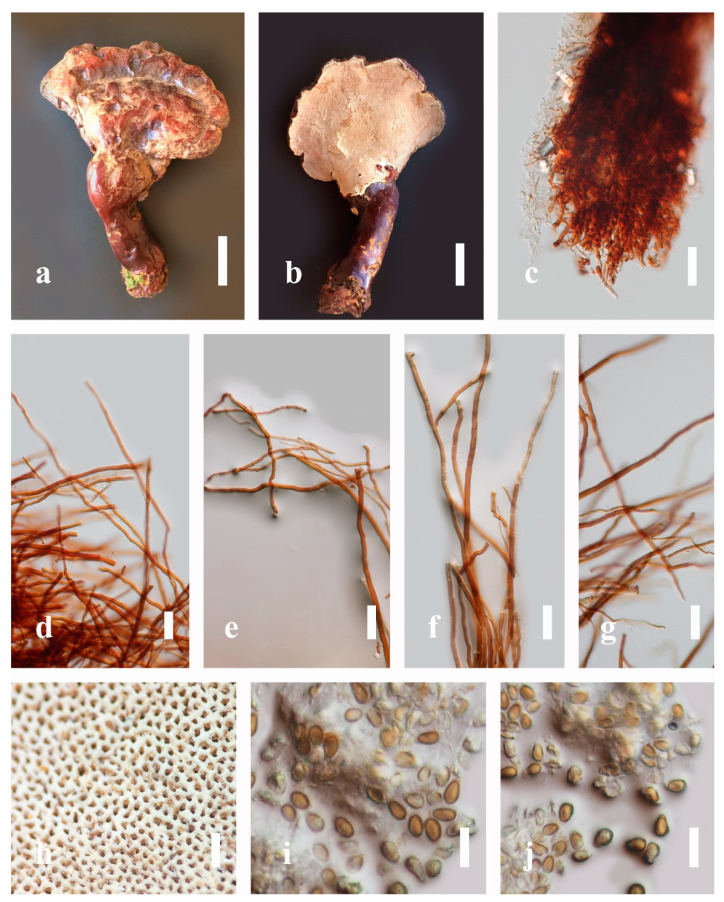
Morphology of *Ganoderma tsugae* (HKAS 97406): (**a**,**b**) mature basidiomes; (**c**) hyphae of tube layers; (**d**–**g**) context hyphae in KOH; (**h**) pore characteristics; (**i**,**j**) basidiospores. Scale bars: (**a**,**b**) = 4 cm, (**c**) = 30 cm, (**d**–**g**) = 30 µm, (**h**) = 500 µm, (**i**,**j**) = 5 µm.

***Ganoderma tsugae*** Murrill, Bulletin of the Torrey Botanical Club. 29; 601 (1902) (Figure 15)

≡ *Fomes tsugae* (Murrill) Sacc. and D. Sacc., Sylloge Fungorum. 17: 123 (1905)

≡ *Polyporus tsugae* (Murrill) Overh.: 714 (1915)

= *Polyporus metallicus* Lloyd, Mycological Writings. 6 65): 1099 (1920)

Facesoffungi number: FoF 06254

*Description:* Basidiomes annual, subdimidiate, stipitate. Pileus 2–16 cm in length, 2–9 cm in width, and 0.5–3 cm thick at the base. Pileus stipitate, subdimidiate to dimidiate, flabelliform, spathulate, umbonate, concentrically sulcate zone, radial from the center extending to the margin, tough to break when dried, often thick at the center, slightly soft at the margin, light in weight when dried, with woody or corky when dried. Pileus surface laccate, convex, radial furrowed, imbricate, incised, glossy, shiny, spathulate, shallow sulcate, umbonate or uneven, strongly laccate and glossy when mature, and weakly laccate where the new hyphae are in active development (margin), usually smooth layers at the center when young to age, irregularly rugose, irregularly ruptured, thin crust overlying the context, tough to break when dried. Pileus color usually homogenous with brownish-red (8C7–8C8) to reddish-brown (8D7–8D8) at the center toward the stipe and margin surface when mature to old. Context up to 0.4–2.2 cm thick near the stipe, brownish-orange (7C7–7C8) to brown (7D8) on the upper layers, brownish-red (8C6) when dried, soft and fibrous, covered with thin crust, some present woody, dimitic hyphal, hyaline, thin-walled with simple septa, branched. Tube 0.3–1.6 cm in length, with dark brown (7F5). Stipe 4–10 cm in length, 2 cm thick, sub-cylindrical to cylindrical, almost stipitate and broad and thick at the base, irregularly ruptured crust overlying, usually strongly laccate with brown (7D8) to dark brown (8F8) when mature, usually dark brown (8F8) when old. Margin soft, some wavy, laccate when mature, and strong laccate when old, brownish-red (8C7–8C8) to reddish-brown (8D7–8D8) from mature to old. Pore 4–6 in number per mm, circular or angular. Pore surface yellowish-white (4A2) when present to yellowish-white (2A2) when mature, discolored when touched, brownish (6E7) when scratched or bruised.

*Hyphal structure:* Hyphal system trimitic, with clamp connections; generative hyphae 3.1–4.8 µm broad (*n* = 30), hyaline, thin-walled, with clamp connections; skeletal hyphae 3.1–6.8 µm broad (*n* = 30), usually hyaline, thick-walled, non-septate, unbranched, and solid; binding hyphae 3.9–5.0 µm width (*n* = 30), with walls varying in thickness, with many branches, some hymenial with sword-like apices in the context. Basidiospores mostly ellipsoid to broadly ellipsoid, with double walls, with size range of (9.7-)10.6–12.7–14.3(-15.8) × (7.3-)8.4–10.7–11.5(-12.4) μm ($\bar{x}$ = 12.7 × 10.5 μm, *n* = 50) μm, with Q = 1.18–1.24, L = 12.68 µm, W = 10.48 µm (including myxosporium), (8.3-)9.4–10.8–12.6(-13.1) × (6.1-)6.9–7.6–8.3(-9.2) μm ($\bar{x}$ = 10.7 × 7.6 μm, *n* = 50) μm, with Q = 1.36–1.45, L = 10.68 µm, W = 7.59 µm (excluding outer myxosporium), overlaid by hyaline, apically and short echinulae, truncate and turgid vesicular appendix, inner walled echinulate, brownish-orange (6C7–6C8), outer walled usually dark-brownish (6F7–6F8) in 5% KOH.

*Habitat:* Solitary, on decaying *Quercus* spp. tree.

*Specimens examined:* CHINA, Yunnan Province, Jinning District, 24°41′17″ N, 102°13′15″ E, 1973 m elev., 11 November 2017, JC. Xu, HKAS 97406.

*Notes: Ganoderma tsugae* has been treated as a synonym of *G. lucidum* [144,145,146]. This fungus is characterized by a laccate and concentric yellowish-red pileus, stipitate, fan-shaped, sulcated with a yellow margin, ovoid, verrucose, and truncated basidiospores. *G. tsugae* is widely distributed across the USA [35,109]. The phylogenic analysis supported *G. tsugae* as an independent species distinct from *G. lucidum*, as it grows exclusively on conifers, especially on *Tsuga* and *Abies* species, while *G. lucidum* inhabits mostly angiospermous trees [76]. According to Loyd et al. [35], *G. tsugae* is similar to *G. oregonense* as they share a distinctly white context tissue, rough basidiospores, and are predominately associated with conifers decay.

#### 3.2.2. Taxonomy of *Ganoderma* from Laos

**Figure 16:**
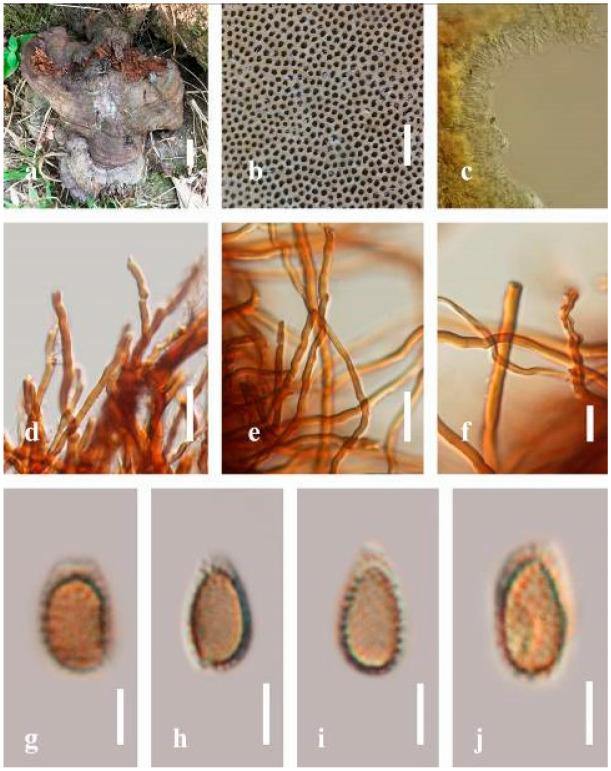
Morphology of *Ganoderma adspersum* (MFLU 19-2177): (**a**) mature basidiomes; (**b**) pore characteristics; (**c**) hyphae of pore; (**d**) hyphae of tube layers in KOH; (**e**,**f**) context hyphae in KOH; (**g**–**j**) basidiospores. Scale bars: (**a**) = 3 cm, (**b**) = 1000 µm, (**d**,**e**) 20 µm, (**f**) = 10 µm, (**g**–**j**) = 5 µm.

***Ganoderma adspersum*** (Schulzer) Donk Proc. K. Ned. Akad. Wet., Ser. C, Biol. Med. Sci. 72(3): 273 (1969) (Figure 16)

≡ *Polyporus adspersus* Schulzer, Flora.: 11 (1878)

= *Polyporus linhartii* Kalchbr., Fungi Hong. 252 (1884)

= *Ganoderma europaeum* Steyaert, Bulletin du Jardin Botanique de l'État à Bruxelles. 31: 70 (1961)

Facesoffungi number: FoF 06241

*Description:* Basidiomes annual, subdimidiate, sessile. Pileus 3–22 cm in length, 2–14 cm broad, and 1–4 cm thick at the base. Pileus sessile, perennial, subdimidiate to dimidiate, flabelliform, spathulate, umbonate, concentrically sulcate zone, somewhat round and plump when young, somewhat imbricate with flabelliform (fan-shaped) when seen from above, broadly attached, radial from the center extending to the margin, tough to break when dried, thick at the base, slightly soft at the margin when mature, light in weight with woody or corky when dried. Pileus surface non-laccate (dull), convex, radially furrowed, incised, spathulate, shallow sulcate, usually silky, soft, and smooth when young, and slippery surface when fresh, thick crust overlaying the context, a differentiated zone at the point of attachment, and tough to break when dried. Pileus color usually homogenous with reddish-orange (7A8) to brown (7D7–7D8) at the center when mature, golden yellow (5B7), brownish-orange (5C5–5C6, 6D5) when old toward the stipe and margin surface. Context up to 0.5–2.5 cm thick near stipe, brown (7D8) to brownish-red (8F8) when mature or dried, soft and fibrous, covered with hard and thick crust, woody when old, trimitic hyphal system present, hyaline, thin to thick-walled, branched. Tube 0.5–1.5 cm in length, usually homogenous with orange (5A7) to dark orange (5A8), reddish-orange (7A7–7A8), and grayish-red (8C7). Stipe 1–5 cm in length, 6 cm thick at the base, almost sessile or some short, stipitate, broad and thick at the base, usually non-laccate, and brown (7D8) to dark brown (8F8) when mature. Margin 0.5–4 cm thick, round, soft, brown (7D8) when mature to old, and usually concolourous with the pileus. Pore 4–6 in number per mm, subcircular to circular. Pore surface yellowish-white (2A2) when mature, discolored when touched, brown (7D8) when scratched or bruised.

*Hyphal structure:* Hyphal system di-trimitic, with clamp connections, orange (6A7) to deep orange (6A8), brownish-yellow (6C8) to brownish-orange (7C8); generative hyphae 1.4–2.8 µm broad (*n* = 30), hyaline, thin-walled, with clamp connections; skeletal hyphae 2.1–4.4 µm broad (*n* = 30), usually hyaline, thick-walled, and solid; binding hyphae 1.5–3.6 µm broad (*n* = 30), with walls varying in thickness, with many branches, some hymenial with sword-like apices in the context. Pileipellis a hymeniderm, brown (6E8) to dark brown (7F6), which is composed of apically clavate-like branched cells. Basidiospores mostly ellipsoid to broadly ellipsoid, sometimes ovoid, with double walls, with a size range of (6.9-)7.5–9.1–9.8(-10.6) × (4.7-)5.4–6.4–7.0(-7.7) μm ($\bar{x}$ = 9.1 × 6.4 μm, *n* = 50) μm, with Q = 1.38–1.45, L = 9.09 µm, W = 6.41 µm (including myxosporium), (5.6-)6.3–7.6–8.4(-9.2) × (4.2-)4.7–5.6–6.1(-6.6) μm ($\bar{x}$ = 7.6 × 5.7 μm, *n* = 50) μm, with Q = 1.35–1.40, L = 7.6 µm, W = 5.52 µm (excluding outer myxosporium), overlaid by hyaline, apically, and short, echinulae, a truncate and turgid vesicular appendix, light yellow (4A4–4A5), grayish-yellow (4B3–4B4) to brownish-orange (5C5–5C6), (5B8) of inner wall, outer wall usually yellowish-brown (5D8, 5E7–5E8) to brown (6D7–6D8) in 5% KOH.

*Habitat:* Solitary, near the roots of a living *Mangifera indica* tree.

*Specimens examined:* LAOS, Luang Namtha Province, 20°35′47″ N, 101°04′07″ E, 935 m elev., 20 June 2018, T. Luangharn, MFLU 19-2177.

*Notes: Ganoderma adspersum* was first reported by Donk [147], who described it as *Polyporus adspersus* Schulzer. *Ganoderma adspersum* is characterized by a distinctive non-laccate, sessile, and applanate pileus. *Ganoderma adspersum* is often confused with *G. applanatum*, *G. australe,* and *Polyporus* [148]. Ryvarden [149] and Ryvarden and Gilbertson [105] considered the correct name of *G. adspersum* as a synonym of *G. australe*, with *G. adspersum* can be differentiated from *G. applanatum* by its thicker at the base, and larger basidiospores, while *G. applanatum* tends to emerge sharply at right angles [105,110], with molecular analysis also supporting the differentiation [45,76,150,151,152,153]. Our collections agree well with the description provided by Ryvarden and Gilbertson [105].

**Figure 17:**
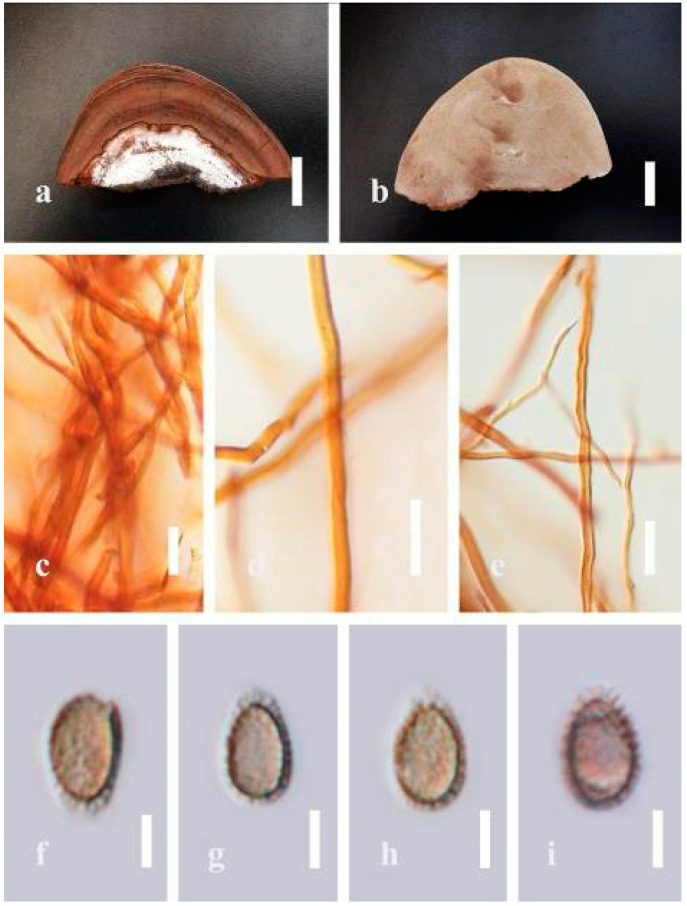
Morphology of *Ganoderma australe* (MFLU 19-2171): (**a**,**b**) mature basidiomes; (**c**–**e**) context hyphae in KOH; (**f**–**i**) basidiospores. Scale bars: (**a**,**b**) = 2 cm, (**c**–**e**) = 20 µm, (**f**–**i**) = 5 µm.

***Ganoderma australe*** (Fr.) Pat., Bull. Soc. mycol. Fr. 5(2, 3): 65 (1889) (Figure 17)

Facesoffungi number: FoF 06242

*Description:* Basidiomes annual, perennial, sessile. Pileus 6–11 cm in length, 2–6.5 cm broad, and 0.8–2 cm thick. Pileus single, flabelliform, subdimidiate, spathulate, umbonate, sulcate, obtuse from the host, broadly attached, consistency hard and tough when mature, tough to break when dried, often thick at the center, slightly soft at the margin, and usually woody and corky when dried. Pileus surface corky, convex, furrowed, spathulate, mostly umbonate or uneven, usually non-laccate (dull) when mature to old, smooth layers when present, deep sulcate at the center, thick and hard crust, irregularly ruptured crust overlying the surface, presented dark brown (7F8) cracked crust when old, and tough to break when dried. Pileus color often homogeneous with pale red (7A5), reddish-orange (7A6–7A7), brown (7D8), to orange red (8B7–8B8) on the upper surface of the base closed to the margin when mature to old. Context up to 0.5–1.2 cm thick near stipe, fibrous, composed of coarse loose fibrils, brown (6D7–6D8), dark brown (6F7) to reddish-brown (8D8, 8D9), covered with thick crust, trimitic hyphal, thick-walled, dense with simple septa, typically with narrow lumen, flexuous, and many branches. Tube 0.4–1 cm in length, brown (7D8) to dark brown (6F8). Stipe sessile with broad attached. Margin white (4A1) when present to mature, soft and slippery when growing fresh, shallow sulcate at the margin, covered and blunt when old. Pore 4–6 in number per mm, subcircular to circular, sometimes angular. Pore surface initially white (4A1), slightly yellowish-white (3A2) when mature, brownish-red (8C4–8C5) when scratched, bruised, or discolored when touched.

*Hyphal structure:* Hyphal system trimitic, with clamp connections, usually brownish-orange (6C5–6C7) in KOH; generative hyphae 2.0–3.4 µm broad (*n* = 30), thin-walled, hyaline, tapering branches, with clamp connections; skeletal hyphae 3.1–4.5 µm broad (*n* = 30), usually thick-walled, sometimes branches, nearly solid; binding hyphae 2.5–3.9 µm width (*n* = 30), usually thick-walled, many branches, nearly solid, and hymenial with sword-like apices in the context. Pileipellis a hymeniderm, brown (7D8), composed of apically acanthus-like branched cells, dextrinoid. Basidiospores mostly ellipsoid to broadly ellipsoid, with double walls, with a size range of (6.5-)7.6–10.1–11.4(-12.5) × (5.9-)6.7–8.5–9.2(-10.3) μm ($\bar{x}$ = 7.2 × 5.9 μm, *n* = 50) μm, with Q = 1.19–1.26, L = 7.24 µm, W = 5.92 µm (including myxosporium), (5.1-)6.2–8.3–9.7(-10.9) × (4.4-)5.6–6.8–7.7(-8.8) μm ($\bar{x}$ = 8.2 × 6.8 μm, *n* = 50) μm, with Q = 1.17–1.26, L = 8.23 µm, W = 6.79 µm (excluding outer myxosporium), overlaid by hyaline, brown apically, bearing fine, distinct, short, echinulae, truncate, turgid vesicular appendix, inner wall light brown (6D4–6D5) to brown (7E7–7E8), and outer wall usually reddish-brown (8E5–8E6, 8F7) in 5% KOH.

*Habitat:* Solitary, on the decaying hardwood of *Canarium* spp. tree species.

*Specimens examined:* LAOS, Luang Namtha Province, 20°35′47″ N, 101°04′07″ E, 935 m elev., 20 June 2018, T. Luangharn, MFLU 19-2171.

**Figure 18:**
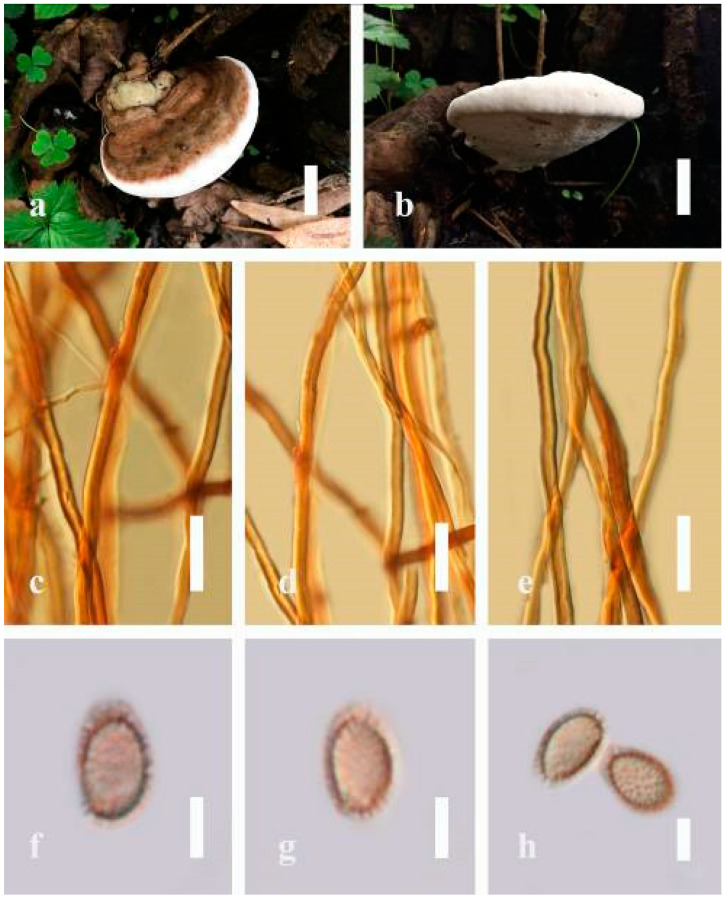
Morphology of *Ganoderma gibbosum* (MFLU 19-2190): (**a**,**b**) mature basidiomes; (**c**–**e**) context hyphae as seen in Melzer’s reagent; (**f**–**h**) basidiospores. Scale bars: (**a**,**b**) = 2 cm, (**c**–**e**) = 20 µm, (**f**–**h**) = 5 µm.

***Ganoderma gibbosum*** (Blume and T. Nees) Pat., Ann. Jard. Bot. Buitenzorg, suppl. 1: 114 (1897) (Figure 18)

Facesoffungi number: FoF 06243

*Description*: Basidiomes annual or perennial, sessile, subflabellate, or subdimidiate. Pileus 2–16 cm in length, 2–9 cm broad, and 0.5–2.3 cm thick. Pileus conks, convex, imbricate, umbonate, uneven, ungulate, usually round when occurring, primordial, somewhat round and plump when young, flabelliform (fan-shaped) when seen from above, broadly attached when mature, thick at the base when mature. Pileus surface non-laccate, smooth when young, silky, soft, and slippery surface when fresh, furrowed on the surface with sulcate to undulating, somewhat spathulate to uneven, incised, compact, hard, and woody when older, covered with a tough crust (0.1–0.2 mm), usually dull and faded when mature to old, and some occurred the lined or cracked crust when older. Pileus color brownish-orange (5C5), reddish white (7A2) at the base, and homogenous with grayish-orange (6B3), brownish-orange (7C5), and light brown (6D4) toward the center of maturity fruiting bodies, white (6A1) at the margin, and usually the color changes to dark brown (8F8) upon touch, becoming grayish-red (8C4–8F6), reddish-brown (8E6) to dull red (10C3) when old. Context up to 0.3–1.3 cm thick, trimitic hyphal with clamp connections, hyaline, with walls varying in thickness, simple septate, composed of narrow, and sparse branches; generative hyphae 1.0–3.4 µm broad (n = 30), with walls varying in thickness, and hyaline; skeletal hyphae 4.0–6.4 µm broad (n = 30) with thick walls; binding hyphal 2.0–6.5 µm broad (n = 30). Hymenophore up to 3 mm in length, with reddish-brown (8D7). Tube layers 0.2–0.8 cm in length, up to 80–163 µm in width, and non-presented when young. Stipe almost sessile, broadly attached when present. Margin wavy, blunt, slippery when wet, thinner at the base and soft than the center, often white (8A1) from youth to maturity, and light brown (6D5) when old. Pore 4–7 in number per mm, when fresh, angular, subcircular to circular. Pore surface white (11A1) to orange white (6A2) when fresh, scratched or bruised, and discolored when touched.

*Hyphal structure:* Hyphal system trimitic, with clamp connections, usually reddish-brown (8D7–8D8); generative hyphae 1.0–3.8 μm broad (*n* = 30), with walls varying in thickness, hyaline, and unbranched; skeletal hyphae 4.2–6.4 μm broad (*n* = 30), light brown (7D6) to brown (7D8) in Melzer’s reagent with dextrinoid, usually thick-walled; binding hyphae 1.8–6.4 μm broad (*n* = 30), brown (6F8) to reddish-brown (8D8) in Melzer’s reagent, thick-walled, many branches, the generative and skeletal hyphae usually intertwined, mostly dark brown (6F7) near the tube layers; Bovista-type ligative hyphae, hymenial with sword-like apices in the context. Pileipellis a hymeniderm, brown (6D8) to light brown (6D6), composed of apically acanthus-like branched cells, dextrinoid. Basidiospores mostly ellipsoid to oblong ellipsoid or broadly ellipsoid, with double walls, with a size range of (4.4-)6.8–8.2–9.5(-10.2) × (3.6-)4.2–5.2–5.8(-6.5) μm ($\bar{x}$ = 8.3 × 5.4 μm, *n* = 50) μm, with Q = 1.49–1.56, L = 8.34 µm, W = 5.44 µm (including myxosporium), (3.6-)5.3–6.0–7.2(-8.3) × (2.8-)3.9–4.5–5.4(-6.2) μm ($\bar{x}$ = 6.2 × 4.5 μm, *n* = 50) μm, with Q = 1.34–1.40, L = 6.24 µm, W = 4.51 µm (excluding outer myxosporium), overlaid by hyaline, dextrinoid, echinulae, inner wall echinulate brown, light brown (6D6–6D8) to brown (6E8) in 5% KOH, and reddish-brown (8F6) to dark brown (8F8) in Melzer’s reagent. Basidia not seen.

*Habitat:* Occasionally on decaying wood of *Pinus* spp.

*Specimens examined:* LAOS, Luang Prabang, 19°51′51″ N, 102°11′39″ E, 589 m elev., 12 July 2018, T. Luangharn, MFLU 19-2190.

***Ganoderma nasalaense*** Hapuar., Pheng. and K.D. Hyde, Mycosphere 10(1): 272 (2019)

Taxonomy and phylogeny analysis are shown in Hapuarachchi et al. [30].

*Notes: Ganoderma nasalaense* is characterized by its duplex context, rigid basidiomes, purplish-black laccate crust, dark brown to gray on the upper pileus surface, brown tube layer, purplish-brown pore surface, trimitic hyphal system, and relatively large, broadly ellipsoid to ellipsoid light brown basidiospores (12.1–13.8 × 9.2–10.5) μm, truncate, with fine and short echinulae (10–12 × 6.5–7.5) µm, and a cuticle composed of strongly amyloid, clavate cells, usually with several irregular lobes or protuberances (30–80 × 3–10.5) µm. This fungus is solitary on decaying hardwood tree trunks in Huaphanh Province, Laos.

#### 3.2.3. Taxonomy of Ganoderma from Myanmar

**Figure 19:**
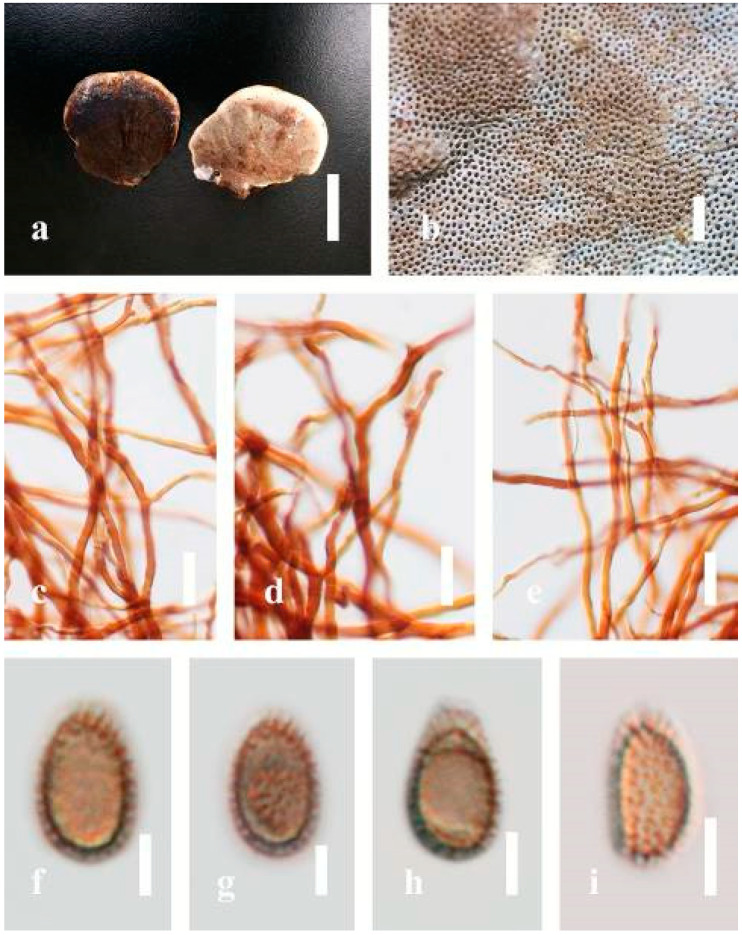
Morphology characteristics of *Ganoderma hoehnelianum* (MFLU 19-2168): (**a**) young basidiomes; (**b**) pore characteristics; (**c**–**e**) context hyphae in Melzer’s reagent; (**f**–**i**) basidiospores. Scale bars: (**a**) = 2 cm, (**b**) = 1000 µm, (**c**–**e**) = 20 µm, (**f**–**i**) = 5 µm.

***Ganoderma hoehnelianum*** Bres., Annales Mycologici. 10 (5): 502 (1912) (Figure 19)

Facesoffungi number: FoF 06260

*Description:* Basidiomes annual or perennial, sessile. Pileus is 0.5–4 cm in length, 0.5–3 cm broad, and up to 0.5 cm thick at the base. Pileus is applanate, umbonate, sub-reniform to reniform, or subflabellate to glabrous from youth to maturity, small in size, obtuse from host, often with undefined concentric zones at the center that extend to the margin, thick at the center, slightly thin and soft at the margin, and tough to break when dried. Pileus surface shiny, smooth, and soft when young, frequently furrowed and shallowly sulcate on the upper surface, which is undulating and somewhat spathulate to uneven in maturity, covered by a thin crust, faded or weakly laccate when young, laccate when mature, and woody when old. Pileus color homogenous, reddish-brown (8E4–8E8) to dark brown orange (9F5–9F8) at the center when mature. Context up to 0.1–0.3 cm thick at the base, brown (7D7–7D8), reddish-brown (8E7–8E8), and dark brown (7F7), abundantly thick-walled, with clamp connections, subsolid hyphae, containing a fibrous pithy context. Tube layers hard and corky, branched, with clamped connections, often brown (7D7) to dark brown (7F8). Stipe almost sessile, broadly attached when present. Margin obtuse from the center, soft and smooth when young, laccate when mature, slightly wavy and slippery when wet, often yellowish-white (3A2) on the upper surface, and pale yellow (4A3) under the margin. Pores 4–6 in number per mm. Pore surface usually white (11A1) to light orange (5A4) when young, grayish-orange (6B4–6B6) when mature, turning light brown (7D5–7D6), brown (7D7), and reddish-brown (8D5–8D7) when scratched or bruised, becoming discolored when touched.

*Hyphal structure:* Hyphal system is dimitic, bearing clamp connections, hyaline, with walls varying in thickness with simple septa and some swollen differentiated zones at the point of attachment, composed of several narrow hyphae, and sparingly branched; generative hyphae (1.8-)2.4–*3.2*–3.7(-4.2) μm broad (*n* = 30) are thin-walled and hyaline; skeletal hyphae (3.3-)3.9–*5.2*–5.8(-6.2) μm broad (*n* = 30) have walls of varying thickness, sometimes subsolid; binding hyphae (2.3-)2.9–*4.2*–4.9(-5.6) μm broad (*n* = 30) are usually thick-walled with many branches, and appear alongside Bovista hyphae, which are usually present from orange (5A6–5A7) to deep orange (6A8) and thin-walled, as well as also reddish-brown (8D7–8D8, 9D8) to brownish-red (9C8) in Melzer’s reagent. Basidiospores are mostly ellipsoid and featuring several ovoid with double walls, (6.7-)7.5–9.8–11.8(-13.2) × (5.7-)6.4–7.8–9.6(-10.8) μm ($\bar{x}$ = 9.7 × 7.8 μm, *n* = 50) μm, with Q = 1.22–1.28, L = 9.73 µm, W = 7.78 µm (including myxosporium), (5.8-)6.4–8.5–9.3(-10.2) × (4.8-)5.3–6.1–6.8(-7.4) μm ($\bar{x}$ = 8.4 × 6.4 μm, *n* = 50) μm, with Q = 1.29–1.35, L = 8.43 µm, W = 6.41 µm (excluding outer myxosporium), overlaid by a hyaline, dextrinoid, and echinulate whose inner wall presents as brownish-orange (5C5–5C6) to brownish-yellow (5C7–5C8) and dark brown (6F8) to reddish-brown (8E7–8E8) in Melzer’s reagent.

*Specimens examined:* MYANMAR, Chin State, Tedim Township, 13 July 2019, P. E. Mortimer, MFLU 19-2168.

*Notes: Ganoderma hoehnelianum* was introduced by Bresadola in 1912 from Indonesia. Of distinctive note are its context color, basidiospore characteristics, and cuticular cells [154]. Wang and Wu [154] reported that the original Chinese *G. hoehnelianum* was an earlier name for *G. shangsiense*, and this fungus was also recorded as *G. shangsiense* in China’s Hainan Province [30,91,120,121]. Our *G. hoehnelianum* is first recorded from Myanmar, and its description is consistent with the descriptions provided by Wang and Wu [154].

**Figure 20:**
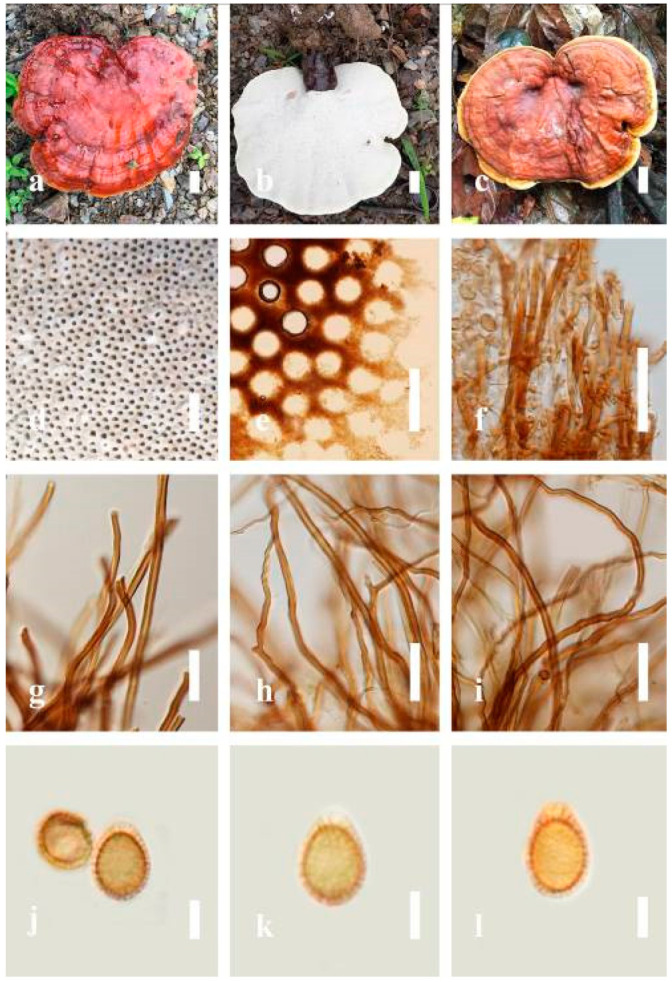
Characteristics of *Ganoderma myanmarense*: (**a**) the upper surface of mature basidiomes of the strain MFLU 19-2167; (**b**) the lower surface of mature basidiomes of strain MFLU 19-2167; (**c**) the upper surface of mature basidiomes of the strain MFLU 19-2169; (**d**,**e**) pore characteristics; (**f**) hyphae of tube layers in KOH; (**g**) context hyphae in KOH; (**h**) context hyphae with clamp connections in KOH; (**i**) hyphae of trama in KOH; (**j**–**l**) basidiospores. Scale bars: (**a**,**c**) = 2 cm, (**d**) = 500 µm, (**e**) = 150 µm, (**f**–**i**) = 20 µm, (**j**–**l**) = 5 µm.

***Ganoderma myanmarense*** Karunarathna, Mortimer, & Luangharn, sp. nov. (Figure 20)

FacesofFungi number: FoF 06262

Index Fungorum number: IF 556794

*Diagnosis: Ganoderma myanmarense* is characterized by its shell-like pileus with strongly laccate appearance colored orange, golden yellow at center, extending reddish-yellow and yellow at margin, usually homogenous reddish-brown when mature, a white to light yellow that indicates active development on the margin of the pileal surface, a white pore surface when fresh, an orange, deep orange to reddish-orange context, and absence of melanoid bands.

*Holotype:* MYANMAR, Chin State, Tedim Township, on dead wood of *Casternopsis* sp., 13 July 2019, LT2019 (MFLU 19-2167).

*Etymology:* The species epithet “*myanmarense*” refers to the country Myanmar, where the holotype specimen was collected.

*Description:* Basidiomes annual or perennial, stipitate. Pileus up to 5–16 cm in length, 4–15 cm in width, and 1–2.5 cm thick at the base. Pileus shell-like (involute from the margin toward the center), sub-reniform to reniform or subflabellate to circular when viewed from above, often with undefined zones at the center that extend to the margin, with a thick center that is slightly soft at the margin, and tough to break when dried. Pileus surface shiny, silky, smooth, and soft when fresh, hard when old, furrowed, sulcate to undulating, somewhat spathulate to uneven, incised, faded or weakly laccate when young, strongly laccate on maturity or when old. Pileus color homogenous, orange (5A6–5A7, 6B7–6B8), golden yellow (5B6), and yellowish-red (8A7–8A8) at the center, slightly reddish-orange (7A7–7A8) and reddish-brown (8E8) where deeply sulcate, reddish-yellow (4A7) and yellow (2A6) at the margin. Context up to 0.6–1.5 cm thick at the base, with some areas thin-walled, thick-walled, along with subsolid hyphae, bearing clamp connections, and absence of melanoid bands. Tube layers woody and hard, usually thin-walled, frequently branched, with clamped connections, and often dark brown (7F7) when dried. Stipe up to 3–8 cm in length, up to 1.3–4 cm in width, centrally stipitate, nearly sub-cylindrical to cylindrical, concolorous with the pileus, often dark brown (9F7–9F8) to violet-brown (11F7–11F8), strongly laccate from maturity to old. Margin obtuse from the center, strongly laccate at the edge, occasionally wavy, slippery when wet, smoother, softer, and thinner, at the base, often yellow (2A6), and deep orange (5A8) to golden yellow (5B7–5B8) on maturity to old. Pores 4–6 in number in number per mm, (60–)80–125(–165) μm, angular, subcircular to circular. Pore surface usually white (11A1), turnins light brown (7D5), brown (7D7–7D8) to dark brown (7F6–7F8) when scratched or bruised, becomes discolored when touched.

*Hyphal structure:* Hyphal system trimitic, with walls of varying thickness, clamp connections present, simple septate hyphae, hyaline, narrow and sparingly branched; generative hyphae (1.2-)1.8–2.2–2.4(-2.7) μm broad (*n* = 30), hyaline, thin-walled; skeletal hyphae (1.8-)2.5–3.8–4.5(-5.0) μm broad (*n* = 30), abundant and thick-walled, sometimes subsolid; binding hyphae (1.5-)1.9–2.7–3.3(-4.3) μm broad (*n* = 30), usually thick-walled. Basidiospores (8.2-)9.3–11.6–12.5(-13.6) × (5.3-)6.0–7.1–7.8(-8.6) μm, (Qm = 1.7, Q = 1.2–2.4, including myxosporium) (*n* = 50), ($\bar{x}$ = 11.5 × 7.1 μm, *n* = 50) μm, with Q = 1.57–1.65, L = 11.52 µm, W = 7.13 µm (including myxosporium), (7.5-)8.3–9.1–10.4(-11.9) × (4.9-)5.4–6.2–6.8(-7.5) μm ($\bar{x}$ = 9.0 × 6.3 μm, *n* = 50) μm, with Q = 1.40–1.48, L = 9.04 µm, W = 6.27 µm (excluding outer myxosporium), mostly ellipsoid to broadly ellipsoid, or globose, double walls, overlaid by hyaline; exosporium (outer wall) smooth, hyaline, endosporium (inner wall) coarse and echinulate, with turgid vesicular appendix, and pale yellow inner wall that can also present in KOH, and yellowish-brown (5E6–5E7) to brown (6E6–6E7) in Melzer’s reagent, outer wall pale orange (6A3–6A4) to orange (6A6–6A7) in KOH, reddish-orange (7A8), grayish-red (7B6–7B8), and dark brown (7F7–7F8) in Melzer’s reagent.

*Habitat:* Solitary near the hardwood root of unknown tree species.

*Additional specimens:* MYANMAR, Chin State, Tedim Township, 13 July 2019, P. E. Mortimer, MFLU 19-2167 (holotype) and MFLU 19-2169 (paratype).

**Figure 21:**
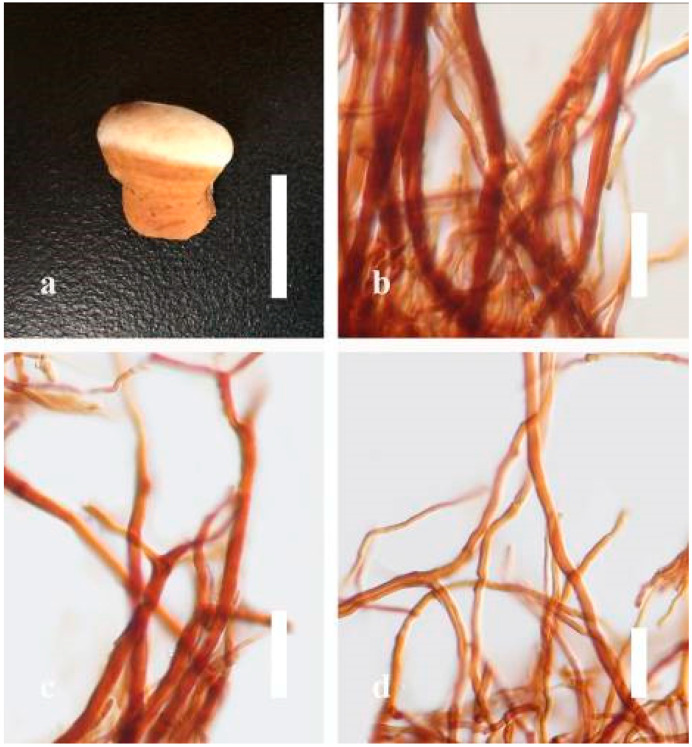
Morphology characteristics of *Ganoderma williamsianum* (MFLU 19-2170): (**a**) young basidiomes; (**b**–**d**) context hyphae in Melzer’s reagent. Scale bars: (**a**) = 2 cm, (**b**–**d**) = 20 µm.

***Ganoderma williamsianum*** Murrill, Bulletin of the Torrey Botanical Club 34: 478 (1907) (Figure 21)

≡ *Elfvingia williamsiana* (Murrill) Imazeki, Bulletin of the Government Forest Experimental Station Meguro. 57: 106 (1952)

Facesoffungi number: FoF 06261

*Description:* Basidiomes annual or perennial, stipitate. Pileus 0.5–1.5 cm in length, 0.5–1 cm in width, and up to 0.5 cm thick at the base. Pileus sub-reniform to reniform, or subflabellate to circular when young, often with concentric zones at the center that extend to the margin, thick at the center, slightly soft at the margin, tough to break when dried. Pileus surface shiny, silky, and soft when young, generally furrowed, smooth, sulcate to undulating, somewhat spathulate to uneven when mature, hard and woody when old, incised on the surface, faded or weakly laccate when young, and usually laccate when mature. Pileus color usually homogenous, orange (5A7–5A8) to deep orange (5B7–5B8), slightly white (5A1), and yellowish-white (3A2, 4A2) at the margin when young. Context is up to 0.3–1.5 cm thick at the base, which is usually thick-walled, with abundant walls varying in thickness, subsolid hyphae containing a fibrous pithy context with clamp connections, and brown (7D8) melanoid bands when mature. The tube layers hard, frequently branched with clamp connections, and often dark brown (7F8). Stipe 1.5 cm in length, 1 cm in width when young, lateral to nearly dorsal, entrally stipitate, almost sub-cylindrical to cylindrical, concolorous with the pileus, thick when young, and often brownish-orange (7C6) to dark brown (9F7–9F8). Margin obtuse from the center, blunted when young, occasionally wavy, slippery when wet, smooth and soft when young (1.5 cm), and often white (3A1) to yellowish-white (3A2) when young. Pore surface white (11A1) to yellowish-white (3A2), turns light brown (7D5) to brown (7D7–7D8) when scratched or bruised, becomes discolored when touched.

*Hyphal structure:* Hyphal system trimitic, bearing clamp connections, hyaline, with walls of varying thickness, simple septate, swollen differentiated zones at the point of attachment, composed of several narrow hyphae, and sparingly branched; generative hyphae (1.6-)1.9–2.3–2.5(-2.6) μm broad (*n* = 30), thin-walled and hyaline; skeletal hyphae (3.1-)3.4–3.9–4.3(-4.8) μm broad (*n* = 30), with walls of varying thickness, with some subsolid; binding hyphae (1.6-)1.9–2.6–2.9(-3.4) μm broad (*n* = 30), usually thick-walled, appearing alongside Bovista hyphae with many branches, whose thick walls usually present as light orange (5A5), orange (5A6–5A7, 6B7–6B8) to deep orange (5A8, 6A8), and reddish-brown (8D7–8D8) to brownish-red (9C8) in Melzer’s reagent. Basidiospores not observed.

*Specimens examined:* MYANMAR, Chin State, Tedim Township, 13 July 2019, P. E. Mortimer, MFLU 19-2170.

*Notes: Ganoderma williamsianum* belongs to the group of laccate *Ganoderma*. This fungus was originally reported in the Philippines and is easily recognized, with its small, dense, ungulate of pileus with pale, yellow pores, large spores, and a short skeletal [2,113]. Among the *Ganoderma* species, there are some similarities between *G. williamsianum* and *G. brownii*, such as both having yellow pores [109]; however, *G. brownii* can be differentiated from *G. williamsianum* by its dull pileus with a hard crust [155], skeletal hyphae with occasional branching, and smaller basidiospores [109,110,137,155]. *Ganodermawilliamsianum* is the earliest valid name for *G. meijiangense* [154], containing similarities to *G. meijiangense*, such as both having sessile, annual crust basidiomes, and white margin, but *G. williamsianum* can be differentiated from *G. meijiangense* by its distinguishing dark brown context, without any layer of black crust and a distinct cuticular composition [61]. Our *G. williamsianum* collection from Myanmar marks a new record, as it shares traits similar to Moncalvo and Ryvarden [2], with sessile, annual crust-like basidiomes, and white margin, trimitic hyphal system bearing clamp connections, hyaline, and walls of varying thickness.

#### 3.2.4. Taxonomy of Ganoderma from Thailand

**Figure 22:**
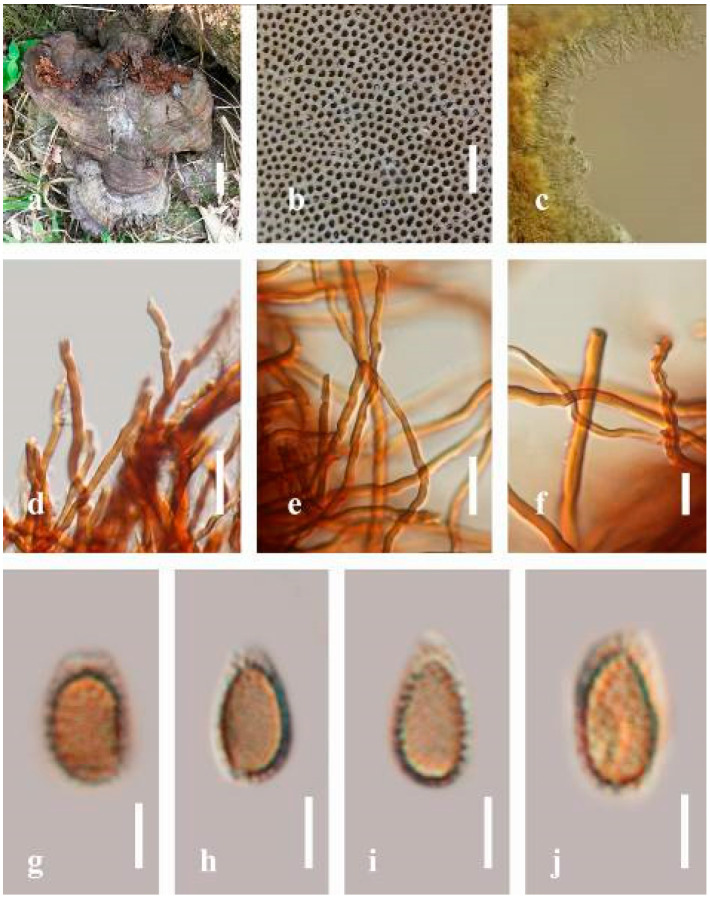
Morphology of *Ganoderma adspersum* (MFLU 19-2178): (**a**) mature basidiomes; (**b**) pores characteristics; (**c**) hyphae of tube layers in KOH; (**d**–**f**) hyphae of thama in KOH; (**g**–**j**) basidiospores. Scale bars: (**a**) = 2 cm, (**b**) = 1000 µm, (**c**–**f**) = 20 µm, (**g**–**j**) = 5 µm.

***Ganoderma adspersum*** (Schulzer) Donk Proc. K. Ned. Akad. Wet., Ser. C, Biol. Med. Sci. (Figure 22)

Facesoffungi number: FoF 06241

*Description:* Basidiomes annual to perennial, applanate, subdimidiate. Pileus 2–14 cm in length, 2–7 cm in width, and 0.5–1.8 cm thick at base. Pileus subdimidiate to dimidiate, flabelliform, spathulate, umbonate, concentrically sulcate zone, sessile or short stipitate, distinctly contracted base, somewhat round and plump when young, somewhat imbricate when viewed from above, flabelliform (fan-shaped), usually broadly attached with radial from center extending to the margin, tough when break, thick at base, slightly soft at the margin when mature, light weight when dried, and woody and corky texture when dried. Pileus surface non-laccate (dull), convex, radial furrowed, incised, spathulate, shallow sulcate, usually silky, soft, smooth when young, and slippery surface when fresh, thick crust overlying the context, differentiated zone at the point of attachment, several layers thick, and leathery when broken. Pileus color usually homogenous with brown (7D7–7D8, 7E7–7E8, 8D8) at center toward stipe to margin surface when mature. Context up to 0.3–1.3 cm thick near the stipe, brown (7D8) to brownish-red (8F8) when mature and dried, soft and fibrous, covered with hard and thick crust, woody when old, trimitic hyphal, hyaline, with walls varying in thickness, with branches. Tube 0.2–1 cm in length, usually homogenous with orange (5A7) to dark orange (5A8), reddish-orange (7A7–7A8), and grayish-red (8C7). Stipe 1–3.8 cm in length, 3.5 cm thick at base, almost sessile, some shortly stipitate, broadly thick at base, usually non-laccate, brown (7D8) to dark brown (8F8) when mature. Margin round, soft, occurring brown (7D8) from mature to old, presented numerous undulations, and usually concolorous with the pileus. Pores 4–6 in number in number per mm, subcircular to circular. Pores surface yellowish-white (2A2) when mature, turns brown (7D8) when scratched or bruised.

*Hyphal structure:* Hyphal system di-trimitic, with clamp connections; generative hyphae 1.2–2.8 µm broad (*n* = 30), hyaline, thin-walled, with clamp connections; skeletal hyphae 2.0–4.4 µm broad (*n* = 30), usually hyaline, thick-walled, solid; binding hyphae 1.6–3.7 µm width (*n* = 30), with walls varying in thickness, many branches, without clamp connections, some hymenial with sword-like apices in the context. Basidiospores mostly ellipsoid, sometimes ovoid with double walls, (7.0-)7.7–9.0–9.9(-10.7) × (4.8-)5.3–6.5–7.1(-7.8) μm ($\bar{x}$ = 8.9 × 6.7 μm, *n* = 50) μm, with Q = 1.30–1.37, L = 8.91 µm, W = 6.69 µm (including myxosporium), (5.8-)6.4–*7.7*–8.6(-9.4) × (4.1-)4.5–5.6–6.0(-6.5) μm ($\bar{x}$ = 7.7 × 5.5 μm, *n* = 50) μm, with Q = 1.35–1.42, L = 7.69 µm, W = 5.52 µm (excluding outer myxosporium), overlaid by hyaline, apically and shortly echinulate, truncate and turgid vesicular appendix, inner wall brownish-yellow (5C7–5C8) and light brown (6D7–6D8), outer wall usually brownish-orange (7C8), brown (7D7–7D8) to dark brown (7E7) in 5% KOH.

*Habitat:* Solitary on decaying *Pterocarpus* sp.

*Specimens examined:* THAILAND, Kanchanaburi Province, 10 November 2018, T. Luangharn, MFLU 19-2178.

**Figure 23:**
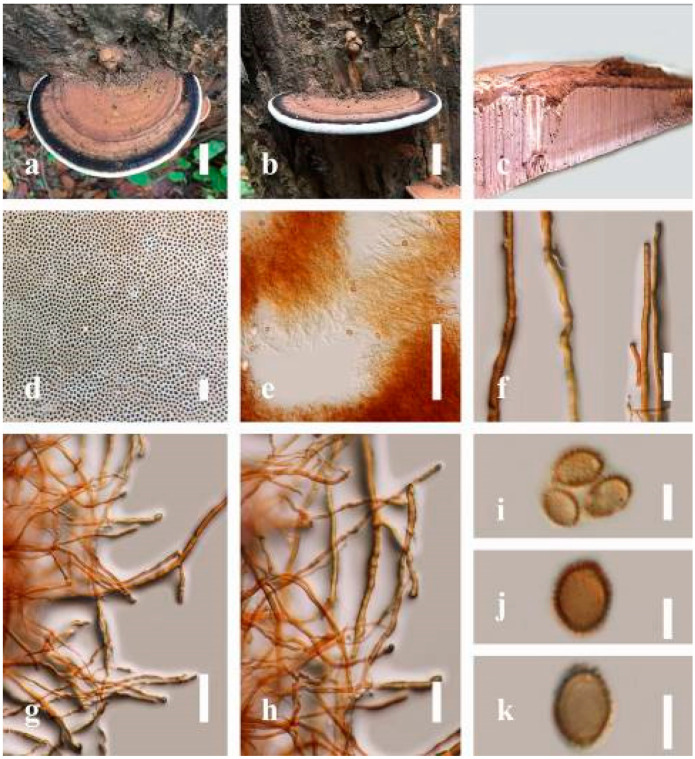
Morphology of *Ganoderma applantum* (MFLU 19-2175): (**a**,**b**) mature basidiomes; (**c**) section of the basidiomes; (**d**) pore characteristics; (**e**) hyphae of pore layers; (**f**) generative hyphae of context in Melzer’s reagent; (**g**,**h**) thick-walled with sparing branched of generative and skeletal hyphae of context in Melzer’s reagent; (**i**–**k**) basidiospores in Melzer’s reagent. Scale bars: (**a**,**b**) = 2 cm, (**d**) = 50 µm, (**e**) = 150 µm, (**f**) = 10 µm, (**g**) = 30 µm, (**h**) = 20 µm, (**i**–**k**) = 5 µm.

***Ganoderma applanatum*** (Batsch) G.F. Atk., Annales Mycologici 6: 189 (1908) (Figure 23)

Facesoffungi number: FoF 06249

*Description:* Basidiomes annual or perennial, subdimidiate to dimidiate, sessile. Pileus 2–12 cm in length, 2.5–6 cm in width, and 0.8–3.1 cm thick at the base. Pileus sessile, perennial, subdimidiate to dimidiate, subflabelliform to flabelliform, convex, imbricate, umbonate or uneven, rarely ungulate, with broadly attached when mature, thicker at base, slightly soft at margin when mature. Pileus surface furrowed, tuberculate to undulate, uneven, and incised when old, non-laccate (dull), compact and hard when mature, woody to corky texture when mature to old, covered with a thin crust (0.1–0.25 mm) overlies the pileus, and some cracked crust when old. Pileus color differentiated zone with peach red (7A4) to reddish-orange (7A6, 7B6) at base, toward to brownish-orange (7C6–7C8) with radius light brown (7D5) zone at center, and extend to dark brown (6F8) closed to active mycelium (margin) of maturity fruiting bodies. Context up to 0.5–2.3 cm thick, mostly brown (6E8) to dark brown (7F6–7F8) of cuticle cells, upper layers light orange (5A5), lower reddish-brown (8D8) with fibrous, some fibrous pithy context, usually separated layers of context tissue at base, and some occurred woody line. Tubes 0.8–2 cm in length, up to 70–160 µm in width, with sulcate at different levels. Stipe almost sessile (without stipe) with broadly attached when present, with differentiated zone at the point of attachment. Margin up to 1 cm thick, round, soft, often white (5A1), turning to light brown (6D4) and brown (6E8) when scratched or bruised, slippery when wet, soft when young, and thin than the center. Pore angular, subcircular, 4–6 in number per mm. Pore surface white (7A1) when fresh, quickly turning to light brown (7D6) to brown (7D8) when handled, scratched, and bruised.

*Hyphal structure:* Hyphal system trimitic; generative hyphae 0.5–2.8 µm ($\bar{x}$ = 2.4, *n* = 30) in diam, clamp connections, almost hyaline, abundant thin-walled and occasionally thick-walled, composed of narrow and sparingly branched; skeletal hyphae 2.7–4.9 µm broad (*n* = 30), usually thick-walled, hyaline, sometimes branched; binding hyphae 1.2–3.6 µm broad (*n* = 30), thick-walled, some branched, and intertwined the skeletal hyphae. Pileipellis a hymeniderm, brown (6E4), which composed of apically acanthus-like branched cells. Basidiospores mostly ellipsoid to broadly ellipsoid, sometimes subcircular with double walls, overlaid by hyaline, exosporium (outer wall) hyaline, endosporium (inner wall) coarse echinulate, with turgid vesicular appendix, truncate at the distal end, (10.0-)10.5–11.3–11.9(-12.4) × (7.2-)8.0–8.8–9.4(-10.2) μm, ($\bar{x}$ = 11.2 × 8.6 μm, *n* = 50) μm, with Q = 1.28–1.33, L = 11.23 µm, W = 8.62 µm (including myxosporium), (6.3-)7.4–8.5–9.3(-10.1) × (4.9-)6.3–7.4–8.7(-9.3) μm ($\bar{x}$ = 8.4 × 7.5 μm, *n* = 50) μm, with Q = 1.09–1.13, L = 8.42 µm, W = 7.58 µm (excluding outer myxosporium), brown (7D7–7D8) in KOH, and reddish-brown (8E6) to dark brown (8F4) in Melzer’s reagent. Basidia 14–20 × 8–10 μm, with four sterigmata.

*Habitat:* Solitary on rotten wood, dead trunks, and decaying stumps of *Artocarpus* spp., and *Dipterocarpus* spp.

*Specimens examined:* THAILAND, Kanchanaburi Province, 10 November 2018, T. Luangharn, MFLU 19-2175.

***Ganoderma australe*** (Fr.) Pat., Bull. Soc. mycol. Fr. 5(2, 3): 65 (1889)

Facesoffungi number: FoF 02906

Characteristics follow Luangharn et al. [59].

*Description:* Basidiomes applanate, spathulate. Pileus 2–7 cm in length, 1–15 cm in width, and 0.5–3 cm thick near the base. Pileus circular, applanate, spathulate, sometimes flabelliform clusters when young, dimidiate, semicircular at maturity, smooth when present. Pileus surface convex, furrow, glabrous, glossy, laccate, and consistently hard when fresh, and tough and light in weight when dry. Pileus color distinct concentric zones with light brown (6D4) to brown (6E8) at the center, slightly pale orange (5A3) to white (5A1) at the margin when young, becoming reddish-brown (8E6) to dark brown (9F8) at the center, dull red (8C4), and pale red (7A3) to white (8A1) at the margin at old age on the upper surface, brown to dark brown when dried, separated by a layer of context, usually brown to gray in winter or may fade as weathering destroys pigments on the pileus surface. Tube layers 0.2–1.2 cm in length, 50–180 µm in width, thick-walled. Stipe 3–5 cm in length, 1.5–3.5 cm in width, 1.3–3.3 cm thick, applanate, with umbo that slightly extended at the base. Margin up to 0.5–3 cm thick, thinner and lighter than the base, soft, round, pale yellow (4A3) to grayish brown (5D3) and reddish gray (8B2), changing to grayish-orange (6E3) when touched, thick toward the margin and downward toward the poreless marginal part of hymenophore. Pore angular, 4–6 in number in number per mm. Pore surface white (2A1) to pale yellow (2A3) in growing specimen, immediately discolored when bruised, cream to grayish brown (5D3) when fresh, with brownish gray (5C2) to brown (4D7) when dried.

*Hyphal structure*: Hyphal system di-trimitic, contextual generative hyphae, binding, and skeletal hyphae, generative hyphae, 1–5 µm broad, with clamp connections, hyaline, thin-walled; binding hyphae 2–6 µm broad, thin to thick-walled, branched, with clamp connections; skeletal hyphae, hyaline, pale brown to brown, thick-walled, 2–7 µm broad. Basidiospores (6.4)6.9–9.3–10.4(11.1) × (5.8)6.4–7.9–8.8(9.7) μm, ($\bar{x}$ = 9.4 × 7.7 μm, *n* = 50), with Q = 1.20–1.21, L = 9.63 µm, W = 7.96 µm (including myxosporium), (5.4)6.3–7.0–8.1(8.9) × (3.4)3.8–5.9–6.5(7.3) μm, ($\bar{x}$ = 7.1 × 5.8 μm, *n* = 50) μm, with Q = 1.19–1.25, L = 7.29 µm, W = 5.93 µm (excluding outer myxosporium), reddish-brown, mostly broadly ellipsoid at maturity, some distinct tapering at the distal end, truncate, double wall, thick-walled inner endosporium. Basidia not observed. Cultures characteristics turned white after incubation at 30 °C for 10 days. Odor distinctive when dried.

*Habitat:* Solitary on hardwood *Shorea robtusa*, or rotten wood, dead trunks, decaying hardwood, decaying stumps, and occasionally occurring on standing trees or trunks of many broad-leaf trees.

*Specimens examined:* THAILAND, Chiang Mai Province, August 2012; T. Luangharn, MFLU 13-0534, MFLUCC 12-0527.

***Ganoderma casuarinicola*** J.H. Xing, B.K. Cui and Y.C. Dai., MycoKeys 34: 93–108 (2018)

Facesoffungi number: FoF 06130

Taxonomy and phylogeny analysis are shown in Luangharn et al. [31]

Notes: *Ganoderma casuarinicola* was collected on a *Pinus kesiya* stump in a pine forest. This fungus is distinctive by its strongly laccate, shallow sulcate, reddish-brown pileus surface, lateral stipe, white pore surface, and brown context. Thai *G. casuarinicola* shows its annual, applanate to dimidiate, 3–16 cm long and 1.5–3 cm wide pileus, larger than Guangdong collection. Our *G. casuarinicola* collections show longer tubes of 6–14 mm, while the tubes of the Guangdong collection are 9 mm long; however, our collections reveal a thinner margin (0.8–1.2 cm thick) than the Guangdong collection (2 cm thick). However, the type of *G. casuarinicola* from the Guangdong collection does not have the melanoid band [37], while our collection features a dark brown melanoid band. Micro- characteristics are dense light brown to brown context layers; walls of varying thickness in generative hyphae; thin-walled binding hyphae; and a thick-walled skeletal. Our *G. casuarinicola* collection has mostly distinctive yellowish-brown basidiospores, with a smaller size range of (8.7)10.8–13.5(14.4) × (6.6)7.6–8.9(9.8) μm than the type of *G. casuarinicola* (8.3-)9.0–10.2(-11.5) × (4.5-)5.0–6.0(-7.0) µm (including myxosporium).

**Figure 24:**
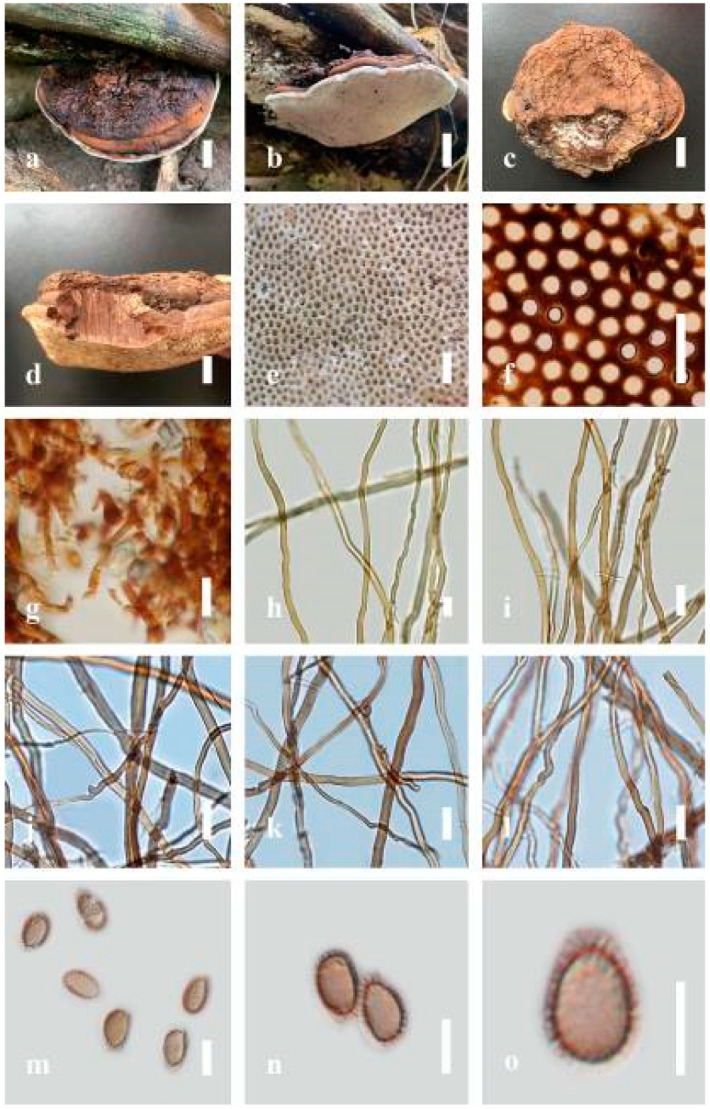
Morphology characteristics of *Ganoderma ellipsoideum* (MFLU 19-2221): (**a**) the upper surface of mature basidiomes when fresh; (**b**) the lower surface of mature basidiomes when fresh. (**c**) the upper surface of mature basidiomes when dried; (**d**) margin; (**e**,**f**) pore characteristics; (**g**) hyphae of tube layers; (**h**,**i**) hyphae from trama in KOH; (**j**–**l**) hyphae from trama in KOH; (**m**–**o**) basidiospores in KOH reagent. Scale bars: (**a**–**d**) = 2 cm, (**e**,**f**) = 500 µm, (**g**) = 200 µm, (**g**–**l**) = 20 µm, (**m**,**n**) = 5 µm.

***Ganoderma ellipsoideum*** Hapuar., T.C. Wen and K.D. Hyde, Mycosphere. 9(5): 951 (2018) (Figure 24)

Facesoffungi number: FoF 06255

*Description:* Basidiomes annual, sessile. Pileus 3–9 cm in length, 2.2–5 cm in width, and 1.5–3.5 cm thick at the base. Pileus annual, convex, imbricate, sessile, umbonate, uneven, ungulate, subflabellate or subdimidiate, somewhat imbricate, when seen from above flabelliform (fan-shaped), usually round, when present primordial, somewhat round and plump when present, broadly attached, thick at base, slightly soft at margin when mature. Pileus surface non-laccate (dull), furrowed, incised, sulcate, smooth when young, undulating on the upper surface, somewhat spathulate to uneven, covered by a thin and hard crust (0.1–0.4 mm), and woody when older. Pileus color usually homogenous with reddish-brown (9E7–9E8) to dark brown (9F7–9F8) at the base at the center, extending white (4A1) to brown (7E7) on the upper margin surface of mature fruiting bodies. Context up to 0.5–2.5 cm thick, compact and hard, trimitic hyphal, with clamp connections, hyaline, thin-walled with simple septa, sparingly branched; generative hyphae 1.2–3.7 µm broad (*n* = 30), hyaline, simple septate, with clamp connections; skeletal hyphae 1.8–4.2 µm broad (*n* = 30), usually thick-walled, unbranched; binding hyphal 2.0–4.8 µm width (*n* = 30) with sparingly branched, thick-walled, without clamp connection. Hymenophore usually brown (7D8) to reddish-brown (8E8). Tube layers 0.5–2.2 cm in length. Margin blunt-edged, wavy, slippery when young, and often white (8A1), where the new hyphae are in active development when young to mature. Pore 4–6 in number per mm, angular, subcircular to circular. Pore surface white (11A1) to pale yellow (2A3), turns brown (7E7) to dark brown (7F7–7F8) when scratched or bruised.

*Hyphal structure:* Hyphal system trimitic, with clamp connections, with brown (7E7); generative hyphae (1.2-)1.6–2.4–3.0(-3.8) μm broad (*n* = 30), branched, thin-walled, hyaline, with grayish-yellow (4B5) in KOH; skeletal hyphae (1.7-)3.1–3.8–4.3(-4.8) μm broad (*n* = 30), dextrinoid, abundant thick-walled, with unbranched; binding hyphae (2.1-)3.1–3.7–4.2(-4.6) μm broad (*n* = 30), thick-walled, frequently branched, usually intertwined the generative and skeletal hyphae, mostly brown (7E7) to dark brown (7F5) near the tube layers; Bovista-type ligative hyphae, hymenial with sword-like apices in the context. Basidiospores mostly ellipsoid with double walls, (4.8-)5.3–6.6–7.2(-7.7) × (3.1-)3.5–*4.3*–5.0(-5.4) μm ($\bar{x}$ = 6.8 × 4.5 μm, *n* = 50) μm, with Q = 1.34–1.43, L = 6.28 µm, W = 4.52 µm (including myxosporium), (3.6-)4.1–5.5–6.0(-6.4) × (1.7-)2.1–2.8–3.3(-3.7) μm ($\bar{x}$ = 5.49 × 2.83 μm, *n* = 50) μm, with Q = 1.83–1.92, L = 5.50 µm, W = 2.94 µm (including myxosporium), overlaid by hyaline, dextrinoid, echinulae, inner wall echinulate brownish-yellow (5C7–5C8) to brown (7D7–7D8) in 5% KOH. Basidia not seen.

*Habitat:* Solitary on rotten wood of *Acacia* sp.

*Specimens examined:* THAILAND, Chiang Mai Province, Mae Taeng, Mushroom Research Centre, 19°07′200″ N, 98°41′44″ E, 652 elev., 14 June 2019, P. E. Mortimer, MFLU 19-2221.

**Figure 25:**
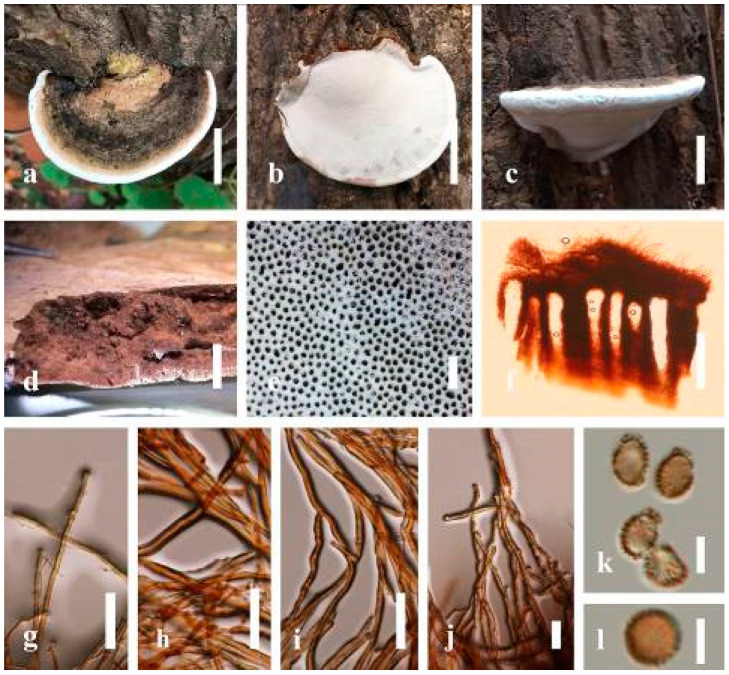
*Ganoderma gibbosum* (MFLU 19-2176): (**a**) the upper surface of mature basidiomes; (**b**) the lower surface of mature basidiomes; (**c**) margin; (**d**) context morphology; (**e**) pore characteristics; (**f**) tube layers; (**g**) generative hyphae of context in Melzer’s reagent; (**h**,**i**) skeletal and binding hyphae of context in Melzer’s reagent; (**j**) hyphae and clamp connections of context in Melzer’s reagent; (**k**,**l**) basidiospores in Melzer’s reagent. Scale bars: (**a**–**c**) = 2 cm, (**d**) = 1 cm, (**e**) = 500 µm, (**f**) = 200 µm, (**g**–**i**,**k**,**l**,) = 5 µm, (**j**) = 20 µm.

***Ganoderma gibbosum*** (Blume and T. Nees) Pat., Ann. Jard. Bot. Buitenzorg, suppl. 1: 114 (1897) (Figure 25)

Facesoffungi number: FoF 06246

*Description:* Basidiomes annual or perennial, sessile. Pileus 2–12 cm in length, 2–8 cm in width, and 0.5–2.8 cm thick. Pileus sessile (without stipe), subflabellate or subdimidiate, convex, imbricate, umbonate, uneven, round when occurring, primordial, round and plump when youth, with broadly attached when mature, and thicker at base slightly soft at margin when mature. Pileus surface non-laccate (dull), smooth, soft, slightly dull and faded when mature to old, usually silky when young, furrowed, tuberculate to undulate, uneven, and incised when mature, compact and hard when mature, woody when mature to old, covered with a thick crust, and some cracked crust when old. Pileus color light orange (6A4–6A5) to grayish orange (6B3–6B5) at base toward the center of mature fruiting bodies become grayish-green (30E2–30E7) on the upper surface when mature to old. Context up to 0.4–2.3 cm thick, tri-dimitic hyphal, with clamps connections, brownish-orange (7C7–7C8) to reddish-brown (8D7–8D8), mostly dark brown (7F7) near the tube layers, Bovista-type ligative hyphae, hymenial with sword-like apices in the context. Tube 0.1–0.6 cm long, up to 80–160 µm in width, and sulcate at different levels. Stipe almost sessile with broadly attached when present. Margin wavy, blunt-edged, slippery when wet, soft when young, thinner than the base and softer than the center, and often white (8A1) when young to mature. Pore angular, subcircular, 4–6 in number per mm in fresh. Pore surface initial white (7A1), turns light brown (7D6) to brown (7D8) when scratched or bruised, with a slippery surface when fresh.

*Hyphal structure:* Hyphal system trimitric hyaline, with walls varying in thickness with simple septa, composed of narrow and sparingly branched; generative hyphae 1.3–4.6 µm broad (*n* = 30), thin-walled and hyaline hyphae; skeletal hyphae 4.0–7.3 µm broad (*n* = 30), thick-walled and hyaline hyphae; binding hyphae 2.8–6.3 µm broad (*n* = 30), usually with walls varying in thickness. Pileipellis a hymeniderm, brownish-orange (6C8) to brown (6D8), composed of apically acanthus-like branched cells, dextrinoid. Basidiospores (4.2-)6.5–8.1–10.3(-11.2) × (3.9-)4.8–4.6–5.7(-6.4) μm ($\bar{x}$ = 8.2 × 4.7 μm, *n* = 50) μm, with Q = 1.70–1.76, L = 8.38 µm, W = 4.85 µm (including myxosporium), (4.5–)6.1–7.2–8.1(-9.2) × (3.9-)4.5–5.3–5.2(-6.9) μm ($\bar{x}$ = 7.4 × 5.4 μm, *n* = 50), with Q = 1.31–1.38, L = 7.53 µm, W = 5.60 µm (excluding outer myxosporium), ellipsoid or some globose with double walls, overlaid by hyaline, dextrinoid, distinct echinulate, inner wall echinulate brown, light brown (6D4) to brown (6E8) in 5% KOH. Basidia not seen.

*Habitat:* Solitary on standing trees of *Dendrocalamus strictus*.

*Specimens examined:* THAILAND, Chiang Rai Province, 19°48′20″ N, 100°04′19″ E, 680 m elev., October 2017, T. Luangharn, MFLU 19-2176.

**Figure 26:**
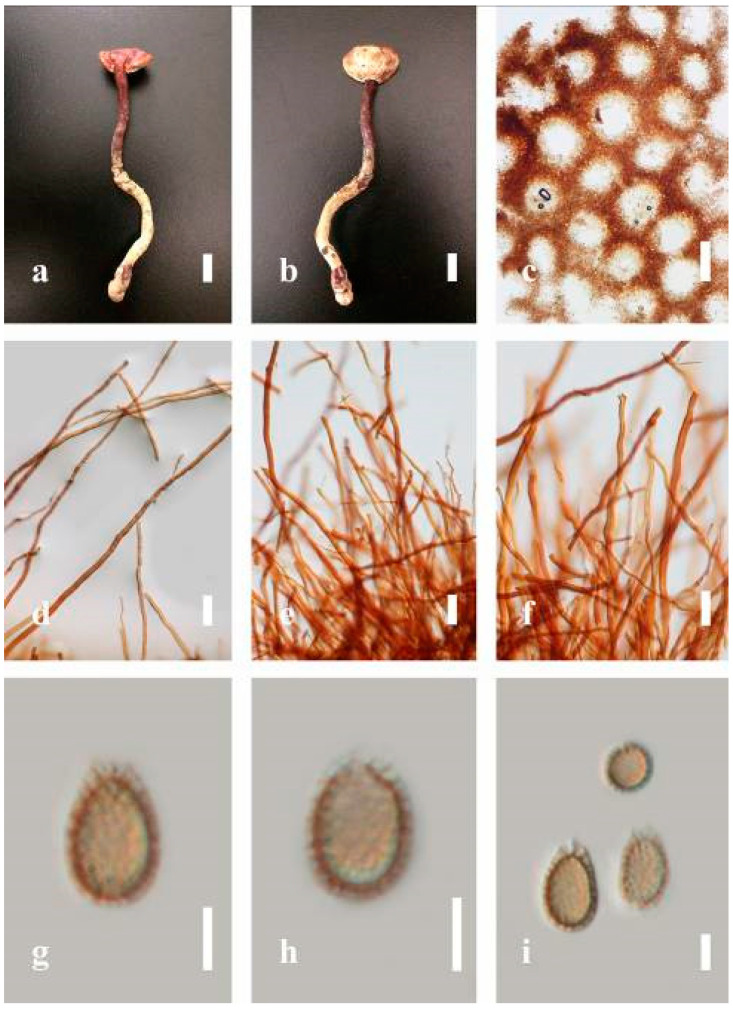
Morphology of *Ganoderma lucidum* (MFLU 19-2162): (**a**,**b**) mature basidiomes; (**c**) pore characteristics; (**d**) generative hyphae of context in KOH; (**e**,**f**) generative. skeletal and binding hyphae of context in KOH; (**g**–**i**) basidiospores. Scale bars: (**g**,**i**) = 2 cm, (**c**) = 200 µm, (**d**–**f**) = 20 µm, (**g**–**i**) = 5 µm.

***Ganoderma lucidum*** (Curtis) P. Karst., Revue Mycologique Toulouse. 3(9): 17 (1881) (Figure 26)

Facesoffungi number: FoF 06250

*Description:* Basidiomes annual or perennial, sub-reniform to reniform, stipitate. Pileus up to 2–6 cm in length, 1.5–3.0 cm in width, and 0.8–2.0 cm thick at the base. Pileus stipitate, sub-reniform to reniform, undefined imbricate, irregular, some laterally, and flabelliform with a contracted, concentrically sulcate zone, irregularly ruptured crust overlying the context, radial from center extending to the margin, tough when broken, often thick at center slightly soft at margin, and leathery when age when broken. Pileus surface smooth layer at center from young to old, usually furrowed, incised, undulate to sulcate, somewhat spathulate to uneven, some woody or corky texture when old, weakly laccate when present, strongly laccate and glossy when mature, and usually weakly laccate where the new hyphae are in active development (margin). Pileus color usually brownish-red (8C7–8C8) at the center, slight to reddish-orange (7B7–7B8), and orange (6A7–6A8) on the upper pileus surface. Context up to 0.4–1.4 cm thick at base, abundant thick-walled, subsolid hyphae, concentric lines of various shades, bearing clamp connections, light brown (6D6) to brown (6D8, 6E8), with dark brown (7F7) melanoid bands. Tube usually hard, brown (7D7) to dark brown (7F7). Stipe up to 8–16 cm in length, up to 0.8–1.5 cm in width, eccentric stipe, cylindrical to slightly flattened, laccate, and reddish-brown (8D7–8D8, 8E7–8E8) from mature to old. Margin often 0.5–1.3 cm, orange (6A7–6A8) upper surface, and reddish-yellow (4A8) under surface, thinner than the base, and softer than the center. Pore 4–6 in number per mm, (70–)110–145(–160) μm, subcircular to circular, sometimes angular. Pore surface white (11A1) when present, yellowish-white (2A3) to light brown (7D6) from young to mature, turning brown (7D7–7D8) to dark brown (6F6) when scratched or bruised.

*Hyphal structure*: Hyphal system trimitic, with clamp connections, hyaline, walls varying in thickness with simple septa, sparingly branched, swollen by melanoid bands, usually brownish-orange (6C7–6C8) to brown (6D7–6D8) in KOH; generative hyphae up to (1.8–)2.0–2.3–2.7(–3.0) μm broad (*n* = 30), usually thin-walled, some thick-walled, with clamp connections, branched, and almost hyaline; skeletal hyphae (3.2–)4.3–5.4–6.1(–6.8) μm broad (*n* = 30), usually thick-walled with clamp connections, with unbranched; binding hyphae (2.4-)2.9–4.4–5.0(-5.9) μm broad (*n* = 30), walls usually varying in thickness with abundant branched and present the melanoid bands. *Basidiospores* ellipsoid, some subglobose to globose with double walls, with a truncate apex, size range of (8.2-)8.8–9.8–10.5 (-11.4) × (5.8-)6.2–6.8–7.5(-8.2) μm, ($\bar{x}$ = 10.0 × 6.9 μm, *n* = 50) μm, with Q = 1.51–1.57, L = 10.65 µm, W = 6.92 µm, (including myxosporium), (6.3-)7.1–7.6–8.2(-8.4) × (4.8-)5.4–5.7–6.1(-6.5) μm, ($\bar{x}$ = 7.5 × 5.8 μm, *n* = 50) μm, with Q = 1.26–1.31, L = 7.57 µm, W = 5.89 µm (excluding outer myxosporium) (*n* = 50), brownish-orange (6C8, 6D8) to brown (6E5) of endosporium (inner wall) with brown (7E7–7E8) exosporium (outer wall) in Congo red, brownish-orange (6C8) in 5% KOH, and yellowish-brown (5D8) in Melzer’s reagent, mostly overlaid by hyaline, coarse echinulate, hyaline turgid vesicular appendix.

*Habitat:* Solitary on decaying hardwood of *Dendrocalamus strictus* in dry evergreen forest.

*Specimens examined:* THAILAND, Chiang Rai Province, 19°48′20″ N, 100°04′19″ E, 680 m elev., 15 October 2017, T. Luangharn, MFLU 19-2162.

**Figure 27:**
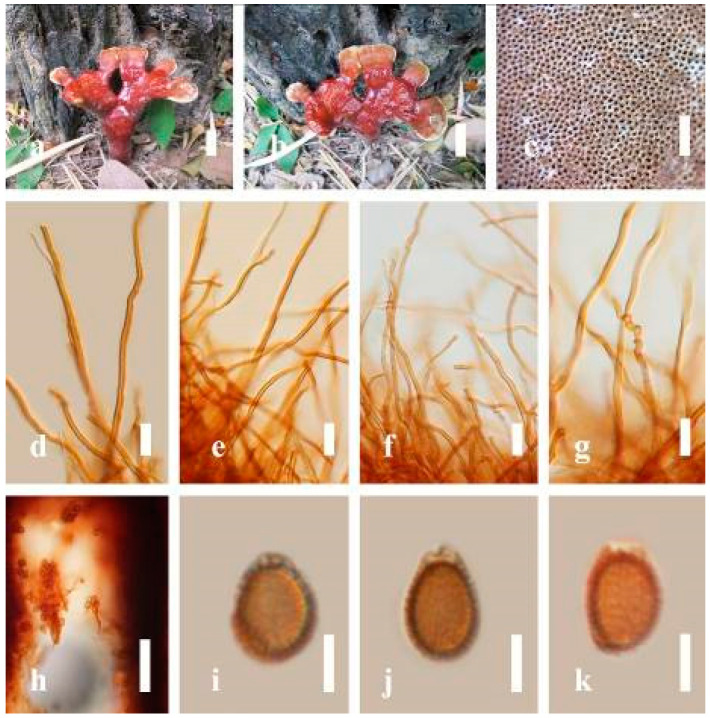
Morphology characteristics of *Ganoderma multipileum* (MFLU 19-2166): (**a**,**b**) mature basidiomes; (**c**) pore characteristics; (**d**) generative hyphae of context in KOH; (**e**–**g**) generative. skeletal and binding hyphae of context in KOH; (**h**) basidiospores with tube layers; (**i**–**k**) basidiospores in KOH. Scale bars: (**a**,**b**) = 3 cm, (**c**) = 1000 µm, (**d**–**g**) = 20 µm, (**h**) = 50 µm, (**i**–**k**) = 5 µm.

***Ganoderma multipileum*** Ding Hou. (1950) (Figure 27)

Facesoffungi number: FoF 06256

*Description:* Basidiomes annual, stipitate. Pileus up to 6–11 cm in length, 4–9 cm in width, and 1–3 cm thick at base. Pileus sub-reniform to reniform, subflabellate to flabellate, concentrically sulcate zone, fleshed at center slightly to margin, radial or branched from center extend to the margin when seen from above, tough when break, often thick at center slightly soft at margin, and leathery when age when break. Pileus surface weakly laccate to strong laccate at center when mature to age, and faded or week laccate at active mycelial (margin), smooth, irregularly ruptured crust overlying the context, some woody or corky texture when old, usually furrowed, incised, sulcate to undulating, and somewhat spathulate to uneven on the surface. Pileus color usually homogenous brownish-red (8C7–8C8) at the base, slight yellowish-red (8A7–8A8, 8B7–8B8) at center, and light brown (7D5) to brown (7D7–7D8) on the upper margin surface. Context up to 0.3–1.2 cm thick at the base, with walls varying in thickness, subsolid hyphae, bearing clamp connections, usually light brown (6D4–6D6) to brown (6D8, 6E8) of hyphae, and dark brown (7F7) melanoid bands in KOH. Tube hard, usually thin-thick-walled, often with brown (7D7) to dark brown (7F7). Stipe up to 4–9 cm in length, up to 1–2.5 cm in width, almost eccentric, sub-cylindrical to cylindrical, plump, strong laccate, often homogeneous with red (9A6) to brownish-red (9C6), and dark red (10C7) when mature to old. Margin often white (4A1) to pale yellow (4A4) where the new hyphae are in active development, light brown (7D5) to brown (7D7) when bruised, strong laccate, wavy, slippery when wet, softer, thin than the base, and soft than the center. Pore angular, 4–6 in number per mm, (99-)120–154(-170) μm in diam, subcircular to circular. Pore surface white (11A1) when present, pale yellow (4A3) to orange white (5A2) when young to mature, light brown (6D4) with age, turning to light brown (7D6), brown (7D7–7D8) when dried or scratched and bruised.

*Hyphal structure*: Hyphal system trimitic, with clamp connections, hyaline, walls varying in thickness with simple septa, sparingly branched, usually light orange (5A4–5A5) to orange (5A6–5A7) in KOH; generative hyphae up to (1.6-)2.2–*3.5*–4.6(-5.2) μm broad (*n* = 30), usually thick-walled, unbranched, flexuous, and almost hyaline; skeletal hyphae (3.8-)4.2–5.1–5.9(-6.7) μm broad (*n* = 30), usually thick-walled, unbranched, sometimes subsolid; binding hyphae (1.4-)2.2–3.6–4.4(-5) μm broad (*n* = 30), usually walls varying in thickness, with flexuous, abundant branched, with Bovista-type binding hyphae, and occurred the melanoid bands. Basidiospores mostly ellipsoid, some ovoide, truncate at maturity, with double walls, (7.6-)8.8–11.7–12.4(-13.1) × (4.9-)5.3–6.1–6.9(-7.4) μm, ($\bar{x}$ = 11.8 × 6.3 μm, *n* = 50) μm, with Q = 1.84–1.89, L = 11.84 µm, W = 6.32 µm (including myxosporium), (5.9-)6.9–8.2–9.3(-10.6) × (4.3-)4.9–5.6–6.0(-6.5) μm, ($\bar{x}$ = 8.3 × 5.8 μm, *n* = 50) μm, with Q = 1.40–1.46, L = 8.36 µm, W = 5.85 µm (excluding outer myxosporium), mostly overlaid by hyaline, brownish-orange (6C8), (6D8) of exosporium (outer wall), endosporium (inner wall) coarse echinulae, with hyaline turgid vesicular appendix, with orange (6A7), (6B7) in KOH.

*Habitat:* Solitary on decaying stump of *Pinus merkusii*.

*Specimens examined:* THAILAND, Prachuap Khiri Khan Province, 12°08′52″ N, 99°45′41″ E, 491 m elev., 26 June 2018, T. Luangharn, MFLU 19-2166.

*Notes: Ganoderma multipileum* was originally reported from Taiwan, PRC [44], which was presented over half a century ago from Taiwan, PRC [39]. This fungus was the earliest valid name with *G. lucidum* from tropical Asia [44]; however, Wang et al. [33] verified that *G. multipileum* is the correct name for this tropical fungus. This fungus is a distinctive form with its laccate to strong laccate pileus, stipitate, rarely sessile, irregularly ruptured crust overlying the context, yellow-brown to dark brown context, cream pore surface, flattened or sub-cylindrical, lateral, horizontally lateral, with ovoid to ellipsoid basidiospores (7.6–13.5 × 5.5–7.5 μm) (with myxosporium), 6.5–10.5 × 4.5–6.5 μm (without myxosporium)), mostly truncate, brown, with a dark brown [33]. Some researchers have shown in their phylogenies that *G. tropicum* is similar to *G. multipileum* [4,5], which is considered most resemble *G. tropicum* in morphology and habitat even though they are distinct species [33] and *G. flexipes* [4,76,129]. Our *G. multiplicatum* was collected from Thailand, are similar to the original description by Wang et al. [33] by its showed laccate to strong laccate pileus, radial or branched from center extends to the margin, with ellipsoid basidiospores.

***Ganoderma orbiforme*** (Fr.) Ryvarden, Mycologia. 92(1): 187 (2000) Figure 28)

≡ *Polyporus orbiformis* Fr., Epicrisis Systematis Mycologici.: 463 (1838)

≡ *Fomes orbiformis* (Fr.) Cooke, Grevillea. 14 (69): 18 (1885)

≡ *Scindalma orbiforme* (Fr.) Kuntze, Revisio generum plantarum. 3(2): 519 (1898)

≡ *Ganoderma lucidum* var. *orbiformis* (Fr.) Rick, Iheringia. 7: 201 (1960)

≡ *Ganoderma orbiformum* (Fr.) Ryvarden (2000)

= *Ganoderma mastoporum* (Lév.) Pat., Bulletin de la Société Mycologique de France. 5: 71 (1889)

= *Ganoderma fornicatum* (Fr.) Pat., Bulletin de la Société Mycologique de France. 5: 71 (1889)

= *Ganoderma boninense* Pat., Bulletin de la Société Mycologique de France 5: 72 (1889)

= *Ganoderma subtornatum* Murrill, Bulletin of the Torrey Botanical Club. 34: 477 (1907)

= *Ganoderma cupreum* (Cooke) Bres., Annales Mycologici. 9: 268 (1911)

= *Ganoderma densizonatum* J.D. Zhao and X.Q. Zhang, Acta mycol. sin.: 86 (1986)

= *Ganoderma limushanense* J.D. Zhao and X.Q. Zhang, Acta mycol. sin.: 219 (1986)

Facesoffungi number: FoF 06257

*Description:* Basidiomes annual or perennial, sessile. Pileus 4–11 cm in length, 3–6 cm in width, and 1–2.4 cm thick at base. Pileus sessile, flabelliform or spathulate, convex, imbricate, umbonate, uneven, ungulate, sub-reniform, sub-orbicular, subdimidiate, obtuse from host, broadly attached, somewhat imbricate, thicker at base, slightly soft at margin when mature. Pileus color usually homogenous with grayish-orange (6B3–6B6), brownish-orange (6C5–6C6), and brown (6D7–6D8) at base extending to the margin of maturity to old. Pileus surface partly non-laccate (dull) to weakly laccate, faded texture when old, furrowed, incised, sulcate, undulating, and somewhat spathulate to uneven on the upper surface, silky and soft when fresh, woody when mature to older, and covered with compact and hard crust (0.1–0.25 mm). Context up to 0.4–1.0 cm thick, trimitic hyphae with clamp connections, hyaline, walls varying in thickness with simple septa, sparingly branched. Hymenophore brown (6D7–6D8) to reddish-brown (8D7). Tube layers 0.3–1.2 cm in length, light brown (6D4–6D5) to brown (7D7). Stipe almost sessile with broadly attached when present, with brownish-orange (6C8). Margin soft, wavy, blunt-edged, slippery when young, with grayish-orange (6B5–6B6) to brownish-orange (6C8) when old. Pore 4–6 in number per mm, circular or subcircular, or angular. Pore surface white (11A1), when present, turns light brown (7D5) to brown (7D7–7D8) when scratched or bruised, becoming discolored when touched.

*Hyphal structure*: Hyphal system trimitic, with clamp connections, usually orange (6A6–6A8) to brownish-orange (6C7–6C8) in KOH; generative hyphae 1.4–5.0 µm broad (*n* = 30), hyaline, thin-walled with clamp connections; skeletal hyphae 2.6–5.6 µm broad (*n* = 30), usually thick-walled, unbranched; binding hyphae 1.2–4.8 µm width (*n* = 30), usually walls varying in thickness, many branches, hymenial with sword-like apices in the context. Basidiospores mostly ellipsoid to oblong ellipsoid or broadly ellipsoid, with double walls, (7.1-)7.9–9.4–11.2(-11.8) × (5.2-)5.97–6.7–7.1(-7.7) μm ($\bar{x}$ = 9.6 × 6.8 μm, *n* = 50) μm, with Q = 1.38–1.44, L = 9.63 µm, W = 6.82 µm (including myxosporium), (6.4-)5.2–6.0–6.7(-10.7) × (3.9-)4.5–5.3–6.1(-6.6) μm ($\bar{x}$ = 6.2 × 5.1 μm, *n* = 50) μm, with Q = 1.20–1.27, L = 6.28 µm, W = 5.10 µm (excluding outer myxosporium), overlaid by hyaline, echinulate, inner wall echinulate brown, some turgid vesicular appendix, light yellow (4A5) to reddish-yellow (4B7–4B7) in 5% KOH. Basidia not seen.

*Habitat*: solitary, on the living tree of *Albizia mollis* in deciduous forest, and living tree of *Indochinese* spp.

*Specimens examined:* THAILAND, Chiang Rai Province, 19°48′20″ N, 100°04′19″ E, 680 m elev., 21 October 2017, T. Luangharn, MFLU 17-1933.

Notes: Ganoderma orbiforme (Fr.) Ryvarden was first described as Polyporus orbiformis, with the original specimen from the tropical region of Guinea in Africa [136]. The fungus was characterized by its distinctive weakly laccate surface or some dull surface areas, brown context, brown pore surface, and brown tube layer, and ellipsoid or ovoid basidiospore. G*anoderma* cupreum, G. densizonatum, G. fornicatum, G. limushanense, *G*. *multiplicatum*, and G. subtornatum are similar to *G*. *orbiforme*; however, taxonomy and molecular analysis treated those taxa as the earliest valid names for *G*. *orbiforme* [7,156]. This fungus has been recorded from China, Laos, Myanmar, and Thailand [30], with our collection is also the collection of *G*. *orbiforme* from Thailand. Our collection from Thailand agrees well with the description by Ryvarden [136] and Wang et al. [156] reported that this fungus posed rigid basidiomes, purplish-black laccate crust, brown pore surface, and tube layer, with ellipsoid or ovoid basidiospores, fine and short echinulate, clavate cells usually with several irregular lobes.

**Figure 29:**
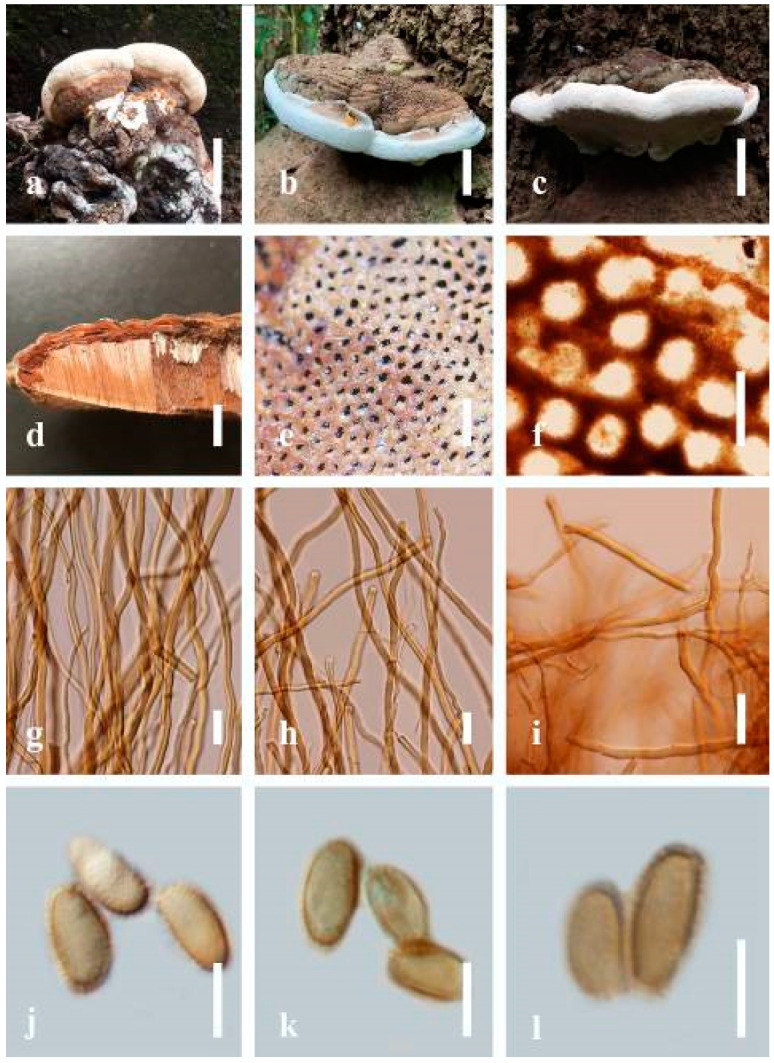
Morphology of *Ganoderma philippii* (MFLU 19-2222): (**a**) mature basidiomes of the collection MFLU 19-2222; (**b**,**c**) mature basidiomes of the collection MFLU 19-2223; (**d**) morphology of the tube layers; (**e**,**f**) pore characteristics; (**g**,**h**) context hyphae; (**i**) hyphae from tube layers; (**j**–**l**) basidiospores in KOH. Scale bars: (**a**–**d**) = 2 cm, (**e**) = 500 µm, (**f**) = 150 µm, (**g**–**i**) = 20 µm, (**j**–**l**) = 5 µm.fi.

***Ganoderma philippii*** Bres. and Henn. ex Sacc. Bres, Iconographia mycological. 21: 1014, t. 1014 (1932) (Figure 29)

≡ *Fomes philippii* Bres. and Henn. ex Sacc., Sylloge Fungorum. 9: 180 (1891)

≡ *Scindalma philippii* (Bres. and Henn. ex Sacc.) Kuntze, Revisio generum plantarum. 3(2): 519 (1898)

= *Fomes pseudoferreus* Wakef., Bulletin of Miscellaneous Informations of the Royal Botanical Gardens Kew. 1918: 208 (1918)

Facesoffungi number: FoF 06258

*Description:* Basidiomes annual, sessile. Pileus 2–9 cm in length, 2–5 cm in width, and 0.4–3.8 cm thick at the base. Pileus annual, convex, sessile, umbonate, ungulate, usually round when present, primordial, plump when present, broadly attached and thick at base, slightly soft at margin. Pileus surface non-laccate to weakly laccate, furrowed, incised, sulcate, smooth when young, undulating on the upper surface, spathulate to uneven, covered with a crust (0.1–0.3 mm), cracked crust when old, and woody when old. Pileus color usually homogenous, brown (7E7–7E8) at base at the center, extending to brownish-orange (6C8), and white (4A1) when present, slightly yellowish-white (2A2) on the upper margin surface of mature fruiting bodies. Context consists of trimitic hyphae, up to 0.3–1.8 cm thick, sparingly branched, walls varying in thickness, compact and hard, with clamp connections, hyaline; generative hyphae 1.8–3.3 µm in width (*n* = 30), brownish-yellow (5C7–5C8), thin-walled, simple septa, hyaline, with clamp connections; skeletal hyphae 2.8–4.9 µm broad (*n* = 30), brownish-yellow (5C7–5C8), usually thick-walled, unbranched; binding hyphae 2.0–4.2 µm width (*n* = 30), brownish-yellow (5C7–5C8) to brownish-orange (6C7–6C8), sparingly branched, walls varying in thickness, and without clamp connections. Hymenophore heterogeneous, brown (7C7–7C8, 7D8), and melanoid band present when mature. Tube layers 0.3–3.8 cm in length. Margin blunt-edged, wavy, and often white (8A1) where the new hyphae are in active development to yellowish-white (2A2) from young to mature. Pore 4–7 in number per mm, subcircular to circular. Pore surface white (11A1) when present, turning brown (7E7) to dark brown (7F7–7F8) when scratched or bruised.

*Hyphal structure*: Hyphal system trimitic, with clamp connections, usually brown (7E7); generative hyphae (1.7-)2.1–2.5–2.9(-3.3) μm broad (*n* = 30), branched, thin-walled, hyaline, unbranched, grayish-yellow (4B5) in KOH, with clamp connections; skeletal hyphae (2.9-)3.4–*4.0*–4.4(-5.0) μm broad (*n* = 30), dextrinoid, abundant thick-walled, with unbranched; binding hyphae (2.1-)2.8–3.3–3.8(-4.3) μm broad (*n* = 30), walls varying in thickness, frequently branched, generative and skeletal hyphae usually intertwined, mostly brown (7E7) to dark brown (7F5) near the tube layer; Bovista-type ligative hyphae, hymenial with sword-like apices in the context. Basidiospores mostly oblong, double walls, (5.8-)6.4–7.3–7.8(-8.2) × (4.1-)4.6–6.1–6.7(-7.1) μm ($\bar{x}$ = 7.4 × 6.0 μm, *n* = 50) μm, with Q = 1.01–1.12, L = 7.32 µm, W = 6.98 µm (including myxosporium), (5.2-)5.7–6.5–7.1(-7.5) × (3.7-)4.3–5.5–6.1(-6.5) μm ($\bar{x}$ = 6.62 × 5.58 μm, *n* = 50) μm, with Q = 1.14–1.24, L = 6.56 µm, W = 5.48 µm (including myxosporium), overlaid by hyaline, dextrinoid, echinulate, inner wall echinulate, grayish-orange (5B4–5B5) in 5% KOH, and yellowish-brown (5E8) to light brown (6D8). Basidia not seen.

*Habitat:* Solitary on rotten wood of unknown tree species.

*Specimens examined:* THAILAND, Chiang Mai Province, Mae Taeng, Mushroom Research Centre, 19°07′200′ N, 98°41′44″ E, 770 m elev., 14 June 2019, P. E. Mortimer, MFLU 19-2222 and MFLU 19-2223.

*Notes: Ganoderma philippii* was introduced as *Fomes philippii* by Bresadola and Hennings in 1891 [126] and later transferred to *Ganoderma* [157]. *Ganoderma philippii* causes red root-rot disease, one of the most economically important diseases across a wide range of commercial perennial woody crops of tropical *Acacia* species [158]. *Ganoderma* *philippii* is distributed across Asia, from the Philippines in the north to southern Papua New Guinea [111]. This species is characterized by its sessile, non-laccate pileus surface, with melanoid bands that form a layer distinct from the context, with a di-trimitic hyphal system with clamped connections overlaid by hyaline, echinulate, inner wall echinulate, sometimes turgid vesicular appendix basidiospores [26]. In this study, our new record of *G. philippii* collected from Thailand is described based on characteristics and phylogenetic analyses. Our collection agrees well with the description by Wang et al. [33].

**Figure 30:**
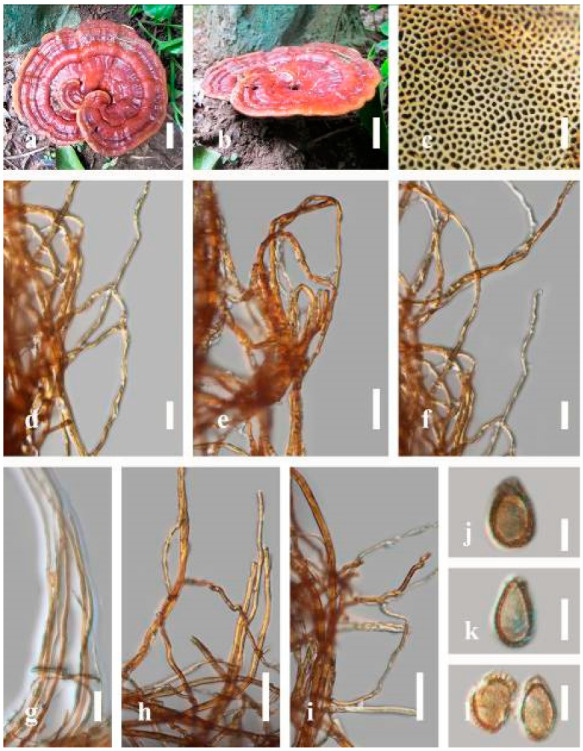
Morphology of *Ganoderma sichuanense* (MFLU 19-2164): (**a**,**b**) mature basidiomes; (**c**) pore characteristics; (**d**–**f**) walls varying in thickness sparing branched of generative and skeletal hyphae of context in upper part in Melzer’s reagent; (**g**) generative hyphae of context in lower part in Melzer’s reagent; (**h**,**i**) thick-walled sparing branched of generative and skeletal hyphae of context in lower part in Melzer’s reagent; (**j**–**l**) basidiospores in Melzer’s reagent. Scale bars: (**a**,**b**) = 2 cm, (**c**) = 500 µm, (**d**) = 50 µm, (**e**,**f**,**h**,**i**) = 20 µm, (**g**) = 30 µm, (**j**–**l**) = 5 µm.

***Ganoderma sichuanense*** S.H. Wu, Y. Cao, and Y.C. Dai. Fungal Diversity. 56(1): 54 (2012) (Figure 30)

Facesoffungi number: FoF 06248

*Description:* Basidiomes annual or perennial, stipitate. Pileus 15 cm in length, up to 10 cm in width, and 2 cm thick. Pileus shell-like (involute from margin into the center), subflabellate or reniform to circular when seen from above, often with undefined concentric zones at center and extend to the margin, thick at center slightly soft at margin, and leathery when age when break. Pileus surface usually laccate, faded or week laccate when young, and strongly laccate when mature to age, shiny, silky, smooth, and soft when fresh, furrowed on the surface with sulcate to undulating, somewhat spathulate to uneven, incised, hard, and woody when old, and some occurred the brown (7D5) lined when older. Pileus color usually homogenous at base at the center with red (9A6–9A7, 9B7–9B8), brownish-red (9C6–9C8), and dark red (10C7–10C8), extended to the margin with reddish-yellow (4A7), but do not change the color when touched. Context up to 0.3–0.5 cm thick at base, abundant thick-walled, some thin-walled, with subsolid hyphae, bearing clamp connections, and occurred the dark brown (7F7–7F8) melanoid bands. Tube layers usually thin-walled, frequently branched with clamp connections, and hard and woody when mature, often brown (7D7). Stipe up to 3–8 cm in lenge, up to 1–3 cm in width, centrally stipitate, almost sub-cylindrical to cylindrical, often red (9A6) to brownish-red (9C6), and dark red (10C7) when mature. Margin obtuse from the center, some wavy, slippery when wet, softer, strong laccate edged, thin than the base and soft than the center, often reddish-yellow (4A7, 4B7–4B8), deep yellow (4A8), and orange (5A6–5A7) to golden yellow (5B7–5B8) when mature to old. Pore angular, subcircular to circular, 4–7 in number per mm, (40-)80–140(-155) μm. Pore surface white (11A1) to yellowish-white (3A2) when young to mature, turning yellow (3B8) to olive-yellow (3C7–3C8) when dried, as well as becoming discolored when touched, turns light brown (7D5), brown (7D7–7D8) to dark brown (7F6–7F8) when scratched or bruised.

*Hyphal structure*: Hyphal system trimitic, with bearing clamp connections, hyaline, with walls varying in thickness with simple septa, composed of narrow and sparingly branched; generative hyphae (1.3-)1.6–1.8–2.2(-2.4) μm broad (*n* = 30), thin-walled, hyaline, branched, with clamp connections; skeletal hyphae (1.6-)2.2–3.5–4.2(-4.9) μm broad (*n* = 30), walls varying in thickness, sometimes subsolid; binding hyphae (1.2-)1.5–1.7–2.0(-2.3) μm broad (*n* = 30), usually thick-walled with narrow to subsolid, with pale orange (5A3) to light orange (5A4) of thin-walled, and orange (6B7) to brownish-orange (6C8) of thick-walled in Melzer’s reagent, and occurred the melanoid bands. Pileipellis a hymeniderm, brown (7D7) with clavate-like cells, dextrinoid. *Basidiospores* mostly ellipsoid or oblong ellipsoid, truncate at maturity, with double walls, (8.0-)9.4–*11.3*–12.2(-13.3) × (4.9-)5.4–*6.5*–7.1(-7.4) μm, ($\bar{x}$ = 11.2 × 6.6 μm, *n* = 50) μm, with Q = 1.64–1.51, L = 11.24 µm, W = 6.69 µm (including myxosporium), (7.8-)8.1–8.6–9.9(-11.7) × (4.9-)5.2–5.9–6.7(-7.4) μm (Qm = 1.5, Q = 1.0–2.0, excluding outer myxosporium) (*n* = 50), ($\bar{x}$ = 8.7 × 5.8 μm, *n* = 50) μm, with Q = 1.46–1.53, L = 8.72 µm, W = 5.84 µm (excluding outer myxosporium), overlaid by hyaline, exosporium (outer wall) smooth and hyaline, endosporium (inner wall) coarse echinulate, with turgid vesicular appendix, light brown (7D7) to brown (7D8) in KOH, and reddish-brown (8E6) to dark brown (8F4) in Melzer’s reagent. Basidia 12–17 × 6–9 μm, with four sterigmata.

*Habitat:* Occasionally occurring on the stump of decaying *Pterocarpus* sp., living tree.

*Specimens examined:* THAILAND, Prachuap Khiri Khan Province, 12°08′52″ N, 99°45′41″ E, 491 m elev., 25 June 2018, T. Luangharn, MFLU 19-2164.

**Figure 31:**
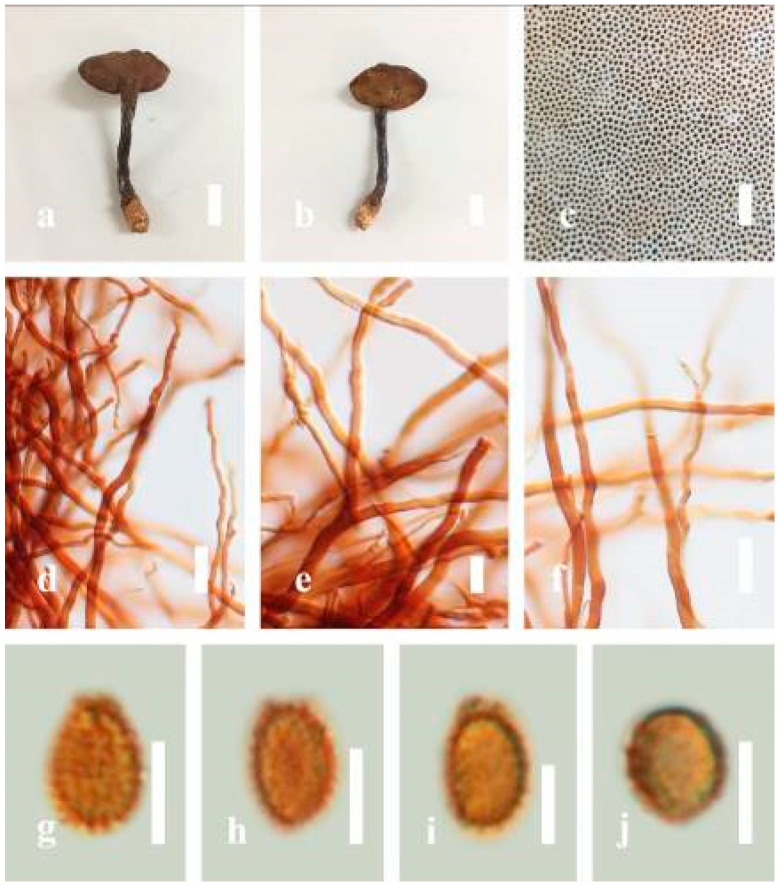
Morphology of *Ganoderma sinense* (MFLU 19-2172): (**a**,**b**) mature basidiomes; (**c**) pore characteristics; (**d**–**f**) generative skeletal and binding hyphae of context in KOH; (**g**–**j**) basidiospores in KOH. Scale bars: (**a**,**b**) = 2 cm, (**c**) = 1000 µm, (**d**–**f**) = 20 µm, (**g**–**j**) = 5 µm.

***Ganoderma sinense*** J.D. Zhao, L.W. Hsu, and X.Q. Zhang, Acta Mycologica Sinica. 19: 272 (1979) (Figure 31)

Facesoffungi number: FoF 06253

*Description:* Basidiomes annual, stipitate, subdimidiate. Pileus 4–10 cm in length, 6–12 cm in width, 0.3–1.3 cm thick at base. Pileus stipitate, subdimidiate to dimidiate, flabelliform, spathulate, umbonate, concentrically sulcate zone, radial from center extending to the margin, tough when broken, often thick at center, slightly soft at margin, light in weight when dried, and non-woody. Pileus surface laccate, convex, some radial furrowed, imbricate, incised, glossy, shiny, spathulate, shallow sulcate when fresh, umbonate or uneven, strongly laccate and glossy when mature, and weakly laccate where the new hyphae are in active development (margin), usually smooth layers at center from young to old age, irregularly ruptured crust overlying the context, and leathery when broken in old age. Pileus color usually homogenous and brownish-red (8C7–8C8) to reddish-brown (8D7–8D8) at center toward stipe and extending brownish-red (9C8) from the center slight to the margin when mature, usually reddish-brown (8E5–8E8) upper margin surface when old. Context up to 0.3–0.8 cm thick near the stipe, dried, upper layers brownish-orange (6C8) when fresh, lower layers grayish-orange (5B5), dark brown (8F7) when dried, soft and fibrous, covered with thin crust, some present woody, trimitic hyphae, hyaline, walls varying in thickness with simple septa, with branches. Tube 0.3–0.9 cm in length, brown (7D8). Stipe 3–12 cm in length, sub-cylindrical to cylindrical, almost stipitate, with broad and thick at base, irregularly ruptured crust overlying, usually strongly laccate, brown (7D8) to dark brown (8F8) when mature, usually dark brown (8F8) when old. Margin soft, some wavy, laccate when mature, weakly laccate when old, brownish-orange (6D8) from mature to old. Pore 4–6 in number per mm, subcircular to circular. Pore surface white (11A1) when present, to yellowish-white (2A2) when mature, turns brownish (6E7) to dark brown (6F7) when scratched or bruised.

*Hyphal structure*: Hyphal system trimitic, with clamp connections, usually light orange (5A5) to orange (5A7), golden yellow (5B7–5B8), sometimes brownish-red (8C7) in KOH; generative hyphae 1.2–2.1 µm broad (*n* = 30), hyaline, thin-walled, with clamp connections; skeletal hyphae 3.2–5.4 µm broad (*n* = 30), usually hyaline, thick-walled, unbranched, and solid; binding hyphae 3.3–5.7 µm width (*n* = 30), walls varying in thickness, with many branches, hymenial with sword-like apices in the context. Pileipellis a hymeniderm, grayish-orange (5B5) to brown (6E8), clavate-like cells, dextrinoid. Basidiospores mostly ellipsoid to broadly ellipsoid, double walls, (9.8-)10.4–11.7–12.5(-13.4) × (7.3-)7.7–8.9–10.0(-10.6) μm ($\bar{x}$ = 11.8 × 9.0 μm, *n* = 50) μm, with Q = 1.29–1.34, L = 11.82 µm, W = 9.02 µm (including myxosporium), (8.9-)9.8–10.4–12.0(-12.8) × (6.2-)6.8–7.2–7.9(-8.3) μm ($\bar{x}$ = 10.6 × 7.3 μm, *n* = 50) μm, with Q = 1.41–1.47, L = 10.47 µm, W = 7.25 µm (excluding outer myxosporium), overlaid by hyaline, apically and shortly echinulate, truncate, some turgid vesicular appendix, inner wall echinulate, orange (5A7), deep (5A8, 5B8), orange (6B8), brownish-orange (6B8), outer wall usually brownish-red (8C7–8C8) in 5% KOH.

Habitat: Solitary, on the living tree of Dendrocalamus strictus and Dipterocarpus sp.

*Specimens examined:* THAILAND, Nakhon Phanom Province, 22 December 2018, T. Luangharn, MFLU 19-2172.

**Figure 32:**
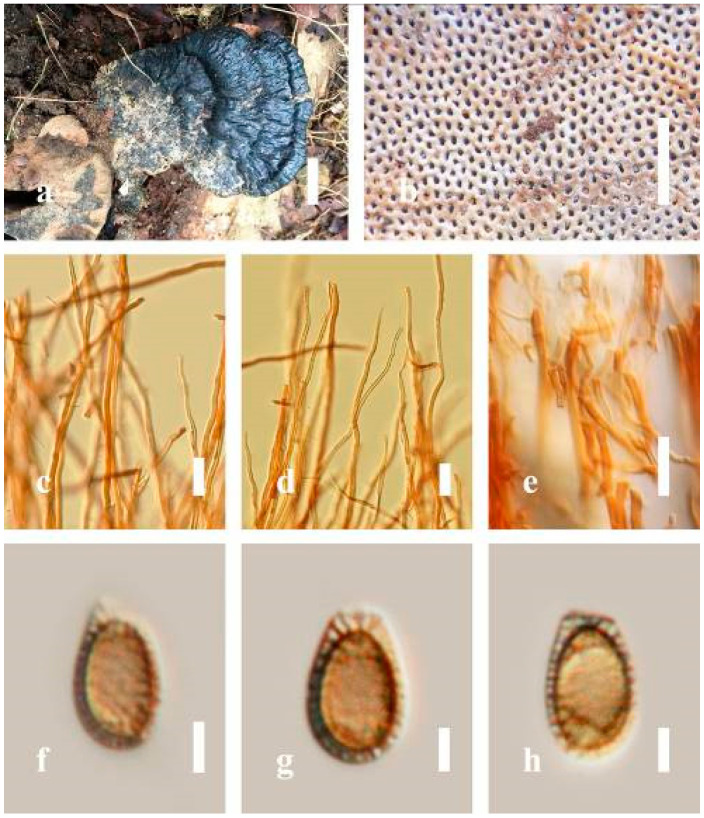
Morphology of *Ganoderma subresinosum* (MFLU 17-1912): (**a**) mature basidiomes; (**b**) pore characteristics; (**c**–**e**) generative hyphae of context in KOH; (**f**–**h**) basidiospores in KOH. Scale bars: (**a**) = 2 cm, (**b**) = 1000 µm, (**c**–**e**) = 20 µm, (**f**–**h**) = 5 µm.

***Ganoderma subresinosum*** (Murrill) C.J. Humphrey, Mycologia. 30 (3): 332 (1938) (Figure 32)

≡ *Fomes subresinosus* Murrill, Bulletin of the Torrey Botanical Club. 35: 410 (1908)

≡ *Trachyderma subresinosum* (Murrill) Imazeki, Bulletin of the Government Forest Experimental Station Meguro. 57: 119 (1952)

≡ *Magoderna subresinosum* (Murrill) Steyaert, Persoonia. 7(1): 112 (1973)

≡ *Amauroderma subresinosum* (Murrill) Corner, Beihefte zur Nova Hedwigia. 75: 93 (1983)

= *Polyporus mamelliporus* Beeli, Bulletin de la Société Royale de Botanique de Belgique. 62(1): 62 (1929)

Facesoffungi number: FoF 06259

*Description:* Basidiomes annual, subdimidiate, sessile. Pileus 6–13 cm in length, 4–10 cm in width, and 0.6–2 cm thick. Pileus stipitate, flabelliform, spathulate, umbonate, subdimidiate to dimidiate, single, concentrically sulcate zone, radial from center extending to the margin, tough when broken, often thicker at center, slightly soft at margin, and light in weight when dried. Pileus surface convex, glossy, shiny, usually frequently furrowed, shallow sulcate, mostly rugulose, spathulate, umbonate to uneven, laccate when mature, strongly laccate when old, concentrically sulcate with irregularly ruptured crust overlying the context, hard from mature to old, and woody or corky texture when old. Pileus color usually dark brown (9F7–9F8) from center to the margin. Context up to 0.3–1 cm thick near the base, dry, upper layers grayish-orange (5B6) when fresh, lower layers grayish-orange (5B5), dark brown (8F7) when dried, covered with crust, woody when dried, trimitic hyphae, hyaline, walls varying in thickness, with simple septa, with branches. Tube 0.4–1.2 cm in length, brown (7D8). Stipe almost sessile, blunt, broadly attached, and thick at base, irregularly ruptured crust overlying, and usually strongly laccate with brown dark brown (9F7) from mature to old. Margin strongly laccate and dark brown (9F7) when mature to old. Pore 4–5 in number per mm, subcircular to circular. Pore surface pale yellow (4A3) to pale orange (5A3), turns dark brown (6F7) when scratched or bruised.

*Hyphal structure*: Hyphal system di-trimitic, with clamp connections, usually light yellow (3A5) to grayish-yellow (3B5), pale yellow (4A5), light orange (5A5) to orange (5B8) in KOH; generative hyphae 1.0–2.1 µm broad (*n* = 30), thin-walled, hyaline, with clamp connections; skeletal hyphae 2.8–5.2 µm broad (*n* = 30), usually unbranched or few branches, and thick-walled; binding hyphae 2.5–5.0 µm width (*n* = 30), walls varying in thickness, many branches, some hymenial with sword-like apices in the context. *Pileipellis* a hymeniderm, light orange (5A5), clavate-like cells, dextrinoid. *Basidiospores* mostly ellipsoid to broadly ellipsoid, double walls, (11.6-)12.1–13.5–14.5(-15.8) × (8.1-)8.9–11.3–11.9(-12.5) μm ($\bar{x}$ = 13.2 × 11.4 μm, *n* = 50) μm, with Q = 1.12–1.19, L = 13.24 µm, W = 11.44 µm (including myxosporium), (10.2-)10.9–12.8–13.7(-14.6) × (6.8-)7.9–8.9–9.6(-10.2) μm ($\bar{x}$ = 12.6 × 8.8 μm, *n* = 50) μm, with Q = 1.41–1.46, L = 12.63 µm, W = 8.84 µm (excluding outer myxosporium), overlaid by hyaline, apically and echinulate, truncate, inner wall usually orange (6A7–6A8) to reddish-golden (6C7) in KOH, brownish-orange (5C5–5C6) in Melzer’s reagent, outer wall usually reddish-orange (7A8, 7B8) in 5% KOH and brown (6D7–6D8) in Melzer’s reagent.

*Habitat:* Solitary on the decaying hardwood of *Peltophorum pterocarpum* and *Castanopsis* sp.

*Specimens examined:* THAILAND, Chiang Rai Province, 20°15′03″ N, 100°14′17″ E, 732 m elev., 9 October 2017, T. Luangharn, MFLU 17-1912.

*Notes: Ganoderma subresinosum* was introduced as *Fomes subresinosus* with the specimen from the Philippines [43]. Next, Humphrey [159] verified this fungus species to the genus *Ganoderma*, and then Imazeki [160] included this species in the genus *Trachyderma*. *Ganoderma subresinosum* is a species that is distributed worldwide, known from the Philippines to other Asian countries, and distributed across Eastern and Central Africa [30,47,100,110]. This fungus is distinctive in form with its laccate pileus, sessile, dark brown pileus surface, and concentrically sulcate with irregularly ruptured crust overlying the context, with ellipsoid to elongate basidiospores. *Ganoderma subresinosum* was regarded as a synonym of *Trachyderma tsunodae* Imazeki [160], *Magoderna subresinosus* [110], and *Amauroderma subresinosum* [113]. In China, this fungus was reported by Chinese researchers as *Fomes subresinosum* [132,161,162] and verified to *A. subresinosum* by Zhao [61] and Zhao and Zhang [13]. Recently, several studies have suggested that those three synonymous fungal species are different from *G. subresinosum* based on emergent morphological and molecular data [163,164]. However, this fungus name is in the Index Fungorum as *G. subresinosum*. So, in this study, we present the first record of *G. subresinosum* from Thailand.

***Ganoderma thailandicum*** T. Luangharn, P.E. Mortimer, S.C. Karunarathna, and J.C. Xu, MycoKeys 59: 55 (2019)

Facesoffungi number: FoF 06129

Index Fungorum number: IF 556535

For characteristics, see Luangharn et al. [31].

*Notes: Ganoderma thailandicum* is characterized by its laccate, deep magenta close to stipe, brownish-red at center, and light yellow around active development toward the margin on pileal surface, white pore surface, brownish-red context, and absence of melanoid band.

***Ganoderma tropicum*** (Jungh.) Bres., Annales Mycologici 8(6): 586 (1910)

≡ *Polyporus tropicus* Jungh., Praemissa in floram cryptogamicam Javae insulae: 63 (1838)

≡ *Fomes tropicus* (Jungh.) Cooke, Grevillea. 14 (69): 19 (1885)

≡ *Scindalma tropicum* (Jungh.) Kuntze, Revisio generum plantarum. 3 (2): 519 (1898)

Facesoffungi number: FoF 05068

For characteristics, see Luangharn et al. [75].

*Notes: Ganoderma tropicum* was introduced as *Polyporus tropicus* by Junghuhn [165] with the specimen from Indonesia. Phylogenetic analysis has been well resolved with the aid of molecular data [5,129] and transferred this species to *Ganoderma* [166], where it is considered a member of the *G. lucidum* species complex [76,167]. The fungus is characterized by its distinctive reddish-brown pileal surface, dark brown context near the tubes, dense context layer, thick near the base, with strongly echinulate basidiospores. Its distribution is highly variable worldwide, scattered across tropical Asian regions, mainland China [4,5,30], South America [137], and Taiwan, PRC [33], and it causes white root and butt rot on several tree species [8]. Furthermore, there are some similarities between *G. tropicum* and other *Ganoderma* species. According to Cao et al. [4], among the Chinese *Ganoderma* species, *G. flexipes*, *G. multipileum*, *G. sichuanense*, and *G. tsugae* are similar to *G. tropicum*, having a reddish-brown pileus surface, dark brown context, ellipsoid basidiospores, strongly echinulate basidiospores, and irregular cuticle cells. Our new record of *G. tropicum* from Northern Thailand was described based on characteristics together with phylogenetic analyses, the details of which are shown in Luangharn et al. [75].

Taxonomy of *Ganoderma* from Vietnam

**Figure 33:**
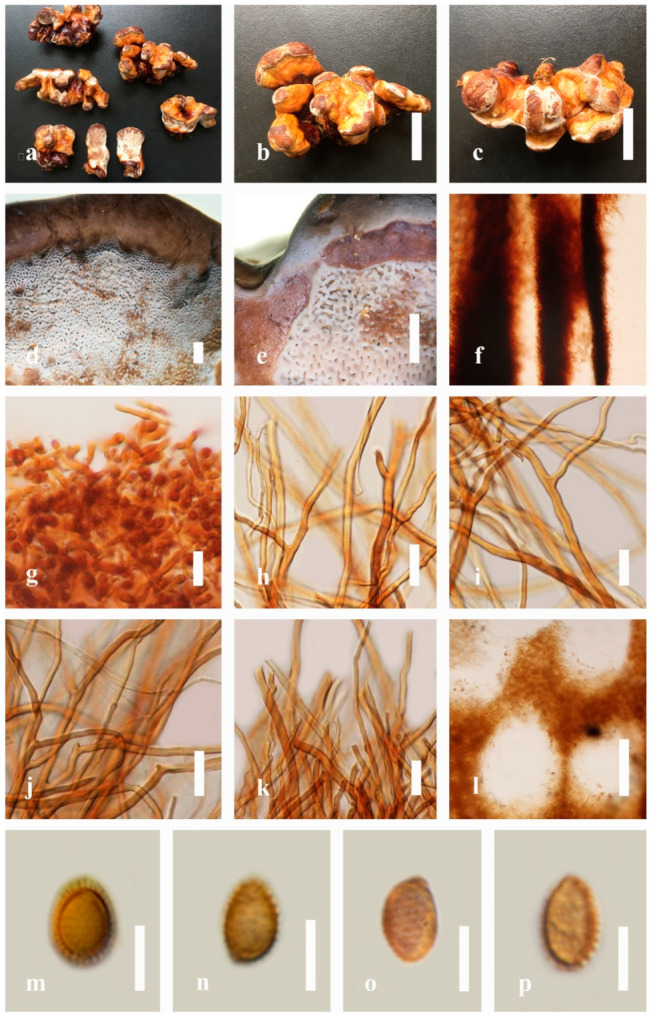
Characteristics of *Ganoderma hochiminhense*: (**a**) basidiomes of the strain MFLU 19-2224; (**b**) mature basidiomes of the strain MFLU 19-2224; (**c**) mature basidiomes of the strain MFLU 19-2225; (**d**,**e**) pores characteristics; (**f**) tube layers; (**g**) hyphae from tube layers; (**h**,**i**) hyphae from context in KOH; (**j**) clamp connection from context; (**k**) hyphae from trama in KOH; (**l**) pores characteristics; (**m**–**p**) basidiospores in KOH. Scale bars: (**b**,**c**) = 2 cm, (**d**,**e**) = 1000 µm, (**g**–**k**) = 20 µm, L = 150 µm, (**m**–**p**) = 5 µm.

***Ganoderma hochiminhense*** Karunarathna, Mortimer, & Luangharn, sp. nov. (Figure 33)

Facesoffungi number: FoF 06334

Index Fungorum number: IF 556794

*Diagnosis: Ganoderma hochiminhense* is characterized by its strongly laccate appearance, with a reddish-brown color near the stipe, a deep orange to brownish-yellow at the center, a white that indicates active development on the margin of the pileal surface, a yellowish-white pore surface when fresh, an orange, deep orange to reddish-orange context, and absence of melanoid bands.

*Holotype:* Vietnam, Hochiminh City, on *Areca* sp. (as described by the seller), 12 June 2019, LT2019 Gano 305 (MFLU 19-2224) and LT2019 Gano 306 (MFLU 19-2225).

*Etymology:* The specific epithet “*hochiminhense*” refers to the place in Vietnam from where the holotype specimen was collected.

*Description:* Basidiomes sessile, orbicular. Pileus up to 0.8–2.2 cm in length, 0.5–1.4 cm width, and 1.3 cm thick at base. Pileus orbicular, undulated, tuberculate, sulcate, single or fused at the base, broadly attached, hard when dried. Pileus surface distinctively laccate when young, strongly laccate when mature or dried. Pileus color reddish-brown from the base, light to deep orange, golden yellow, brownish- to reddish-yellow at the center, with deep yellow to orange yellow at the margin of the upper surface, generally white where new hyphae are in active development, glossy, shiny, smooth, spathulate, sulcate when fresh, with a thin crust overlying the pileus, which is thicker at the base than at the margin, light weight when dried. Hymenophore mostly orange to brownish-orange in KOH, up to 0.8 cm thick, with a dense but not fully homogenous context layer, bearing distinct layers of concentric growth zones at the center that extend to the margins, thick near the base, non-corky or woody texture when dried, bearing a simple septum at base, tough to break when dried, melanoid bands absent. Context reddish-orange in KOH; generative hyphae up to 2.72–3.82 μm ($\bar{x}$ = 3.54, *n* = 50) in diam, almost colorless, thin-walled, some expanded at the apex, unbranched, with clamp connections; skeletal hyphae-dominant, up to 3.56–7.47 μm ($\bar{x}$ = 5.85, *n* = 50), orange to brown, thick-walled, unbranched, without clamp connections; binding hyphae 3.23–5.96 µm ($\bar{x}$ = 4.32, *n* = 50), almost colorless, walls varying in thickness, with some narrow lumen to subsolid, frequently branched, tortuous, and interwoven at the distal end. Tubes hard, brown, up to 0.2–0.5 cm long. Tubes layers generative hyphae 2.21–3.03 µm in diam, pale brown to brown, thin-walled, some thick-walled, with clamp connections, unbranched; skeletal hyphae 3.23–6.15 µm in diam, distinctly brown, thick-walled, some narrow lumen to subsolid, frequently branched; binding hyphae 2.28–4.86 µm in diam, brownish-yellow, thick-walled to solid, and frequently branched. Stipe short stipe, laccate when developing to maturity, and strongly laccate from maturity to old age. Margin white when present to maturity, light brown to brown between the young to mature stages, turning light brown when dry, and silky, soft, and slippery to the touch between youth and maturity, usually bruising when touched and tough to break. Pore angular to round, 4–6 in number per mm, with dissepiments slightly thick to thick. Pore surface white when fresh, yellowish-white when dried, turns brown to dark brown when touched.

*Hyphal structure:* Hyphal system trimitic, with tissues yellow, light orange to orange and brown in KOH; generative hyphae up to 2.70–3.25 μm ($\bar{x}$ = 3.09, *n* = 50) in diam, almost colorless, thin-walled, unbranched, with clamp connections, some slightly swollen at the distal end; skeletal hyphae-dominant, up to 3.49–7.67 μm broad ($\bar{x}$ = 5.98, *n* = 50), pale brown to distinctly brown, thick-walled, unbranched, without clamp connections, occasionally with narrow lumen, some subsolid, interwoven; binding hyphae 3.12–5.87 µm broad ($\bar{x}$ = 4.28, *n* = 50), grayish-orange, walls varying in thickness, frequently branched, tortuous, and interwoven in the distal end. Basidia clavate, 4-sterigmatic, 10.2–14.3 × 8.6–12.5 µm, yellowish to pale brown in KOH. Basidiospores ellipsoid, sometimes broadly ellipsoid or almond-shaped at maturity, reddish-yellowish, light brown to brownish-orange, slightly truncate, double walls, exospore smooth, endospore with coarse echinulate, (6.8)8.5–10.4(11.8) × (5.8)6.9–9.3(10.2) μm ($\bar{x}$ = 9.4 × 8.8 μm, *n* = 50), with Q = 1.02–1.14, L = 9.38 µm, W = 8.82 µm (including myxosporium), (6.6)7.9–9.4(10.9) × (5.4)6.3–8.5(9.3) μm ($\bar{x}$ = 9.0 × 7.7 μm, *n* = 50) μm, with Q = 1.08–1.21, L = 8.98 µm, W = 7.73 µm (excluding outer myxosporium), strongly echinulate, cuticle cells irregular. Culture characteristics white active mycelium on PDA, reaching 8 cm diam after 14 days at 25 °C.

*Habitat*: solitary, on *Areca* spp. stumps.

*Specimens examined*: VIETNAM, Hochiminh City local market, 8°54′32″ N, 98°31′09″ E, 427 m elev., 12 June 2019, S.C. Karunarathna, LT2019 Gano 305 (MFLU 19-2224, holotype) and LT2019 Gano 306 (MFLU 19-2225, paratype).

## 4. Discussion and Conclusions

The present study demonstrates the nomenclatural status, characteristics, phylogenetic analysis, host preference, growing season, climate, and substrate details of *Ganoderma* species from the GMS. Detailed characteristics and molecular analyses allow us to define the *Ganoderma* species used in our study as a distinctive well-supported clade, within *Ganoderma*, with new insights to resolve species delimitation. Altogether, this study describes a total of six new records and two new species of *Ganoderma* comprising two new records (*G. hoehnelianum* and *G. williamsianum*) as well as a new species (*G. myanmarense*) from Myanmar, four new records (*G. ellipsoideum*, *G. multipileum*, *G. philippii*, *G. subresinosum*) from Thailand, and one new species (*G*. *hochiminhense*) from Vietnam.

*Ganodermaorbiforme* specimens were collected from living *Albizia mollis* and *Indochinese* spp. in a deciduous forest from Thailand. These *G. orbiforme* specimens from the Thailand group in the non-laccate *Ganoderma* clade with *G. orbiforme* from Laos [30] and China [37]. The Thai *G. orbiforme* shares similar features as mentioned by Hapuarachchi et al. [30] and Wang et al. [156], such as possessing an annual to perennial, flabelliform or applanate pileus, with a weakly laccate surface, the presence of several thick layers, an undulate margin, and subcircular or circular pores. The pileus size of the Thai collection of *G. orbiforme* (4–11 × 3–6 cm) is within the range of Asian collections (3.5–21 × 2.5–12 cm) [63] but exceeds the Chinese collection (3–8 × 2.5–6 cm) [88]. These findings are consistent with Ryvarden [136], who demonstrated that *G. orbiforme* are widely distributed in tropical and temperate regions. The present study also reports an additional record of *G. orbiforme* from a tropical region in Thailand.

Three *G. sinense* were collected from decaying as well as living *Albizia mollis* (Wall.) Boiv. and *Quercus* spp. in temperate Yunnan Province, China, and two collections were gathered from living *Dendrocalamus strictus* (Roxb.) Nees. and *Dipterocarpus* spp. in tropical Thailand. The present collections of *G. sinense* grouped together as a sister taxon to the *G. nasalaense* from Laos. *Ganoderma sinense* is regarded as having a high degree of phenotypic plasticity and genetic diversity and was associated with substantial intraspecific morphological variation [88,167]. When considering the morphological differences between these, the Chinese *G. sinense* has a smaller-sized pileus (2–6 × 2–4 cm) and brownish-orange context, while the Thai *G. sinense* has a larger-sized pileus (4–10 × 6–12 cm) with a brown to dark brown context. However, the Chinese and Thai *G. sinense* also share some characteristics, such as having stipitate, subdimidiate to dimidiate basidiocarps with a laccate radial pileus that extends from the center to the margin, a colored dark brown, cylindrical stipe, and basidiospores measuring 8.5–11.8 × 7.2–10.6 μm. The current findings of *G. sinense* are in agreement with Hapuarachchi et al. [88], who demonstrated morphological variations across *G. sinense* collected from Hainan and Guizhou Provinces, China. In this study, *G. sinense* from Yunnan Province, China, and Thailand were also recorded.

*Ganoderma tropicum* was introduced by Luangharn et al. [75] based on the collection made from northern Thailand. In this study, phylogenetic analysis demonstrated that this fungus served as the sister clade to *G. multipileum*, *G. parvulum*, and *G. destructans*, and the Thai *G. tropicum* also grouped together with the Chinese *G. tropicum*, forming a sister clade with *G. multiplicatum* and *G. philippii*.

The present study describes a new species, *G*. *hochiminhense* (MFLU 19-2224 and MFLU 19-2225), from Vietnam. Phylogenetic analyses revealed *G*. *hochiminhense* collections grouped as sister taxa to the laccate *Ganoderma* clade, and morphological characteristics and molecular analyses provided insights to resolve species delimitation. *Ganoderma hochiminhense* grouped together as a distinct clade with 100% ML, 98% MP, and 0.99 PP support. *Ganoderma hochiminhense* forms a sister clade with *G. zonatum* from the U.S. [76], the holotype *G. ryvardenii* from Cameroon [98], and the holotype *G. angustisporum* from Africa [37]. However, *G*. *hochiminhense*, *G. zonatum*, and *G. ryvardenii* share similarities and are sessile, laccate to strongly laccate on the upper pileus surface, and ellipsoid to broadly ellipsoid with truncated apices basidiospores at maturity [76,98].

Although in the present phylogenetic tree *G*. *hochiminhense* clustered with *G. zonatum* and *G. ryvardenii*, their macro- and micro-characteristics differ. *Ganoderma hochiminhense* can be easily distinguished from *G. zonatum* by the size range of its small fruiting bodies (0.8–2.2 × 0.5–1.4 cm diam), single or fused fruiting bodies on hosts, orbicular when present, undulated pileus, reddish-brown at base, deep orange, golden yellow, reddish-yellow at its center from maturity to old age, deep yellow and orange yellow on the margin, white pore, reddish-orange context, (6.6–11.8 × 5.4–10.2 μm) basidiospore size range, while the fruiting bodies of *G. zonatum* are brown on the upper pileus surface, have cream pores, are finely echinulated, (9.4–8.8 × 9.0–7.7) basidiospore size range. *Ganoderma ryvardenii* differs from *G*. *hochiminhense* by its large pileus size range (13 × 8.5 cm), annual, dimidiate, or circular pileus, reddish margin, dark brown context, and basidiospore size range (9.0–14.0 × 4–8 μm). *Ganoderma zonatum* was regarded as a species distributed in subtropical to tropical climates [57,136,168], and our *G. hochiminhense* was also collected from a tropical region. Consequently, we propose that taxa in this clade comprise species isolated from tropical regions. This study confirmed that the new *G. hochiminhense* was collected on *Areca* spp. stumps in tropical Vietnam. Considering overall morphology, *G. hochiminhense* appears to be similar to *G*. *boninense* Pat. However, *G*. *boninense* is readily distinguished from *G*. *hochiminhense* by its thicker and broader basidiocarp, reddish bay pileus with reddish-brown pores, and differently sized basidiospores 10–12 × 7–8 μm in *G*. *boninense* vs. 6.8–11.8 × 5.8–10.2 μm (including myxosporium), 6.6–10.9 × 5.4–9.3 μm (excluding outer myxosporium) in *G*. *hochiminhense* [41].

For detailed characteristics and phylogenetic analyses of *G. casuarinicola*, *G. thailandicum*, and *G. enigmaticum*, see Xing et al. [37] and Luangharn et al. [31]. In the present study, *G. calidophilum* collected from China formed a sister clade with the above three *Ganoderma* species, and the clade is distinguished with suitable statistical support values. Although *G. calidophilum* is sister to the laccate clade, its macro-characteristics are very distinctive, such as a stipitate, subdimidiate to dimidiate pileus, homogenous brownish-red, reddish-brown at the center, brownish-orange toward the stipe, light brown at the margin from maturity to old age, brownish-orange context, and cylindrical stipe.

*Ganoderma williamsianum* was collected from tropical Myanmar, and the phylogenetic analysis grouped the present collection with *G. williamsianum* collected from Thailand [37]. Both *G. williamsianum* strains grouped as sister taxa to the holotype *G. mbrekobenum* from Ghana [90], with low support. The description of our collected *G. williamsianum* is distinctive, having characteristics such as small fruiting bodies with a size range of 0.5–1.5 × 0.5–1 cm, rarely with a stipe-like base, subflabellate to circular fruiting bodies, smooth and soft, weakly laccate, and homogenous orange to deep orange context, though the basidiospore could not be observed. The current study allows for comparison between *G. williamsianum* and *G. mbrekobenum. G. mbrekobenum* is macro-micro morphologically very different from *G. williamsianum*, with its distinctive dimidiate, lateral stipe, dimitic hyphal system, with ovoid to broadly ellipsoid basidiospore [90]. Xing et al. [37] also reported the collection of *G. williamsianum* from Thailand with providing a phylogenic analysis of their specimens but failing to provide any morphological descriptions. *Ganoderma williamsianum* has been reported as a species distributed across China [154], Indonesia [110,155], Malaysia [110], and the Philippines [110,169]. Here, this study introduces a new record of *G. williamsianum* from Myanmar.

*Ganoderma adspersum* was collected near the root of the living tree *Mangifera indica* L. in Laos, while the Thai *G. adspersum* strain was collected from the decaying hardwood of *Pterocarpus* spp. Macro-characteristics of our collected *G. adspersum* share features with what has already been described by Moncalvo et al. [45], such as having a non-laccate, sessile, perennial, subdimidiate to dimidiate pileus, a sulcate, brown pileus surface and context, and a yellowish-white pore surface, with a similar basidiospore size range (6.9–10.6 × 4.7–7.8). However, some characteristics are also quite different, as the *G. adspersum* from Laos is mostly ellipsoid to broadly ellipsoid, with brownish-orange inner walls of basidiospores, while the Thai *G. adspersum* is mostly ellipsoid, with brownish-yellow inner walls and brown to dark brown outer walls. It was observed that our Thai *G. adspersum* had a di-trimitric hyphal system, while Hapuarachchi et al. [30] reported that their Thai *G. adspersum* was composed of a trimitic hyphal system. This fungus is frequently reported in temperate and tropical climates [45,152], where it grows on many hardwood tree species [152,170]. *Ganoderma adspersum* has been reported in Europe [171], India [150], Korea [92], Italy [172], Myanmar, and Thailand [30]. In this study, additional specimens of *G. adspersum* from Laos and Thailand are presented for use in updated descriptions of this species using detailed morphological observations and phylogenetic studies.

This study introduces a new record of *G. ellipsoideum*, collected from Chiang Mai Province, Thailand, growing on rotten wood. The newly collected *G. ellipsoideum* serves as the sister group to *G. adspersum* (ML = 97%, MP = 96%, PP = 0.95). It is also correlative to the results by Hapuarachchi et al. [88]. Our collection from Thailand allows us to conduct comparisons with the holotypes of *G. ellipsoideum* from Hainan Province, China. The current *G. ellipsoideum* specimen shares similarities with the holotype collection in its annual, sessile, several-layers-thick circular or subcircular pores, and presence of ellipsoid basidiospores. However, the Chinese holotype of *G. ellipsoideum* presents quite a different basidiospore size range (6.1–7.3 × 3.7–4.6 μm), including myxosporium, while the Thai *G. ellipsoideum* was (4.8–7.7 × 3.1–5.4 μm) including myxosporium. The Chinese *G. ellipsoideum* was initially yellowish-white, later having a yellowish-brown to brownish-yellow pore surface, while Thai *G. ellipsoideum* presented white (11A1) to pale yellow (2A3) coloration. *G. ellipsoideum* also serves as the sister group to the *G. gibbosum* and *G. austral* clade. Our *G. gibbosum* collections were collected from China, Laos, and Thailand. *G. gibbosum* macro- and micro-characteristics are quite different. For variations in *G. gibbosum,* see Luangharn et al. [173], while *G. australe* from Thailand has been described in Luangharn et al. [5].

*Ganoderma multipileum was* collected from Thailand after the rainy season. Phylogenetic analysis showed our *G. multipileum* specimen matched closely within the clade to *G. multipileum* (CWN 04670) from Taiwan. Our collection was grouped as a sister to *G. steyaertianum* B. J. Smith and Sivasith, *G. mizoramense* Zothanzama, Blanchette, Held, C.W., Barnes, *G. martinicense* Welti and Courtec, and *G. destructans* M.P.A. Coetzee, Marinc. and M.J. Wingf. These *Ganoderma* species, including *G. steyaertianum*, *G. mizoramense*, *G. martinicense*, *G. destructans*, and *G. multipileum*, share similarities in that they have a laccate to strongly laccate upper pileus surface at maturity. Although *G. steyaertianum* can be easily distinguished from *G. multipileum* by its distinctive kidney-shaped basiodomata with a dark brown pileus surface, this species is also similar to *G. multipileum* in that its basiodomata rarely extend completely to the margin [128]. *Ganoderma mizoramense* can be differentiated from *G. multipileum* by its reddish-brown when fresh to liver-brown when dry upper pileus surface, dark-brownish to dark-reddish brown context, and lower surface white when fresh [94]. *Ganoderma martinicense* can be differentiated from *G. multipileum* by its generally sessile basidiomata and dark-cinnamon-brown context [35], while our *G. multipileum* specimen rarely displayed sessile basidiomata and had a light brown to brown context; however, phylogenetic analysis revealed *G. martinicense* as a close relative to the Asian taxon *G. multipileum* [35,93]. *G. destructans* is significantly different, with its pileus containing a creamy and soft context and presenting mostly ovoid basidiospores [9].

*Ganoderma philippii* was collected in tropical Chiang Mai Province, Thailand. Phylogenetic analysis reveals it to be close to Malaysian *G. philippii* strains [97]. *Ganoderma philippii* is a unique species that grows especially actively in heavy soils with high water content [111]. Macro- and micro-characteristics were also correlative to results by Singh et al. [26], who reported this fungus as easily identifiable by its non-laccate pileus surface and sessile basidiome mostly presenting oblong basidiospores. *Ganoderma philippii* formed a sister clade to *G. multiplicatum* and *G. tropicum*. *G. philippii* are very different, with their distinctive non-laccate, often convex, umbonate, and plump pileus and sessile, with mostly oblong basidiospores, while *G. multiplicatum* is laccate to strongly laccate, radial or branched from the center, extending to the margin, and presenting mostly as ellipsoid basidiospores [33]. *G. tropicum* was collected from Thailand; although it closed a sister clade with *G. multiplicatum*, its macro- and micro-characteristics are noticeably different and were detailed by Luangharn et al. [31].

In this study, our *G. sichuanense* specimens were collected in the temperate climate zone of Yunnan Province, China, and a tropical part of Thailand. Phylogenetic analysis placed our collections as sister taxa to the laccate *Ganoderma* clade and close to the previously reported *G. sichuanense* from China [99]. Our collections also share macro- and micro-characteristics similar to the holotype described of *G. lingzhi* [4], with their shell-like, reniform to circular pileus, reddish-brown pileal surface, strongly laccate, stipitate, and mostly present ellipsoid basidiospores that truncate at maturity; however, *G. lingzhi* was verified to *G. sichunense* in the records of Index Fungorum. Among the Chinese *Ganoderma* species, *G. flexipes*, *G. multipileum*, *G. sichuanense*, *G. tropicum*, and *G. tsugae* are the species that most closely resemble *G. sichuanense*, as their macro- and micro-characteristics share a reddish-brown pileal surface, similar basidiospores, and cuticle cells. Nevertheless, differences between these *Ganoderma* species have been detailed in Dai et al. [34] and Yao et al. [174].

*Ganoderma resinaceum* has long been reported in temperate climates [38,76], and our collection is also from a temperate part of Yunnan Province, China. Our Chinese *G. resinaceum* is close to the *G. resinaceum* collected in England and the Netherlands, with suitable clade support. Our result is also similar to the phylogenetic analysis by Douanla-Meli and Langer [46]. However, *G. resinaceum* from England and the Netherlands lacks macro-micro descriptions. Moreover, *G. resinaceum* collections have also been reported from India [143], although the macro- and micro-characteristics of these Indian specimens differ from our Yunnan collections. The Yunnan strain of *G. resinaceum* is distinctive with its short stipe, varying reddish-brown upper pileus surface, grayish-yellow to dark brown context covered by an irregularly ruptured thin crust, and larger pileus size range (1.5–12.5 × 1–7 cm), while the Indian strain is stipitate, with a brown upper pileus surface, round margin, light brown context, and smaller pileus size range (8–9 × 6–7.5 cm) [143]. Therefore, we conclude that despite phylogenetic similarities placing the various specimens, from temperate and tropical regions, of *G. resinaceum* in the same clade, there remains a high degree of morphological variance within this species [38,46,76,143].

We introduce *G. hoehnelianum* as a new record from Myanmar. This mushroom was collected on the decaying stump of an unidentified dicotyledonous tree species in tropical Myanmar. The phylogeny of our *G. hoehnelianum* specimen is close to the Chinese *G. hoehnelianum* collection [91] and is well supported. Our collection shares similar macro- and micro-characteristics to Wang and Wu [154] and Hapuarachchi et al. [88], with its distinctive annual, sessile, plano concave, dark brown upper pileus surface and context, and concentrically sulcate zones with broadly ovoid basidiospores. Our *G. hoehnelianum* grouped with the *G. austroafricanum* H. Xing, B.K. Cui, and Y.C. Dai and *G. carocalcareum* Douanla-Meli clade. *G. austroafricanum* is distinctive from *G. hoehnelianum*, with its reddish-brown pileus surface, smooth surface, round margin, with subglobose basidiospores. *Ganoderma carocalcareum* collected from a tropical region in Cameroon is distinctive from *G. hoehnelianum*, with its friable context, and thick toward the margin and downwards to the pore margin (part of hymenophore), red-brown to brown-orange margin, and forming a concentrical aporoid zone [46].

*Ganoderma applanatum* was collected from temperate China and tropical Thailand. Phylogenetic analysis shows our *G. applanatum* clustering as a distinctive group with suitable support. The name *G. lipsiense* has been treated and corrected to *G. applanatum* [15], and it is mentioned that *G. applanatum* also belongs to this complex species. Macro- and micro-characteristics of our collections are similar to those described in detail in Ryvarden and Gilbertson [105]. Our Chinese and Thai *G. applanatum* collections have quite different macro-characteristics. The Chinese *G. applanatum* shows a smaller pileus size range (1.5–5.8 × 0.5–4.5 cm) and is up to 1.5 cm thick at the base, while the Thai *G. applanatum* was 2–12 × 2.5–6 cm and 0.8–3.1 cm thick at the base. The Chinese *G. applanatum* is distinctive and non-laccate, faded from maturity to old age, while Thai *G. applanatum* had differentiated zones of peach red and reddish-orange at the base, brownish-orange with the radius, light brown at the center, and extending to dark brown close to active mycelium (margin). Our results are also correlative to Wang et al. [5], who has reported on the high morphological variation within Chinese *Ganoderma*. *Ganoderma* species from different geographic areas have also shown separate lineages in phylogenetic analyses [38,153].

In this study, we describe a new *Ganoderma* species, *G*. *myanmarense*, collected in tropical Myanmar. The two *G*. *myanmarense* collections grouped as sister taxa to the laccate *Ganoderma* clade, and their characteristics and molecular analyses provided insights to resolve species delimitation. This new species, *G*. *myanmarense*, forms a sister clade with *G. wiiroense* E.C. Otto, Blanchette, C.W. Barnes and Held. from Ghana, *G. destructans* M.P.A. Coetzee, Marinc, M.J. Wingf. from South Africa, and our *G. flexipes* from China. *Ganoderma* *wiiroense* has an annual, dimidiate pileus; sessile, yellowish-brown to dark-reddish-brown basidiocarps [101], while our *G*. *myanmarense* has its distinctive sectorial to shell-shaped, sub-reniform to reniform pileus; golden yellow, yellowish-red at center, slightly reddish-orange and reddish-brown (8E8) basidiocarps. *Ganoderma destructans* has its distinctive globular pileus, with creamy soft non-poroid tissues [9], and *G. flexipes* is very different from *G*. *myanmarense* by having sub-reniform to reniform pileus, small pileus size range (0.5–3.2 × 0.5–3 cm), homogenous reddish-brown to dark brown upper pileus surface, and sub-cylindrical to the cylindrical stipe.

Our *G. lucidum* collections came from both China and Thailand. Phylogenetic analysis showed our strains clustered with *G. lucidum* from France, with suitable statistical support. The results indicated that our *G. lucidum* grouped as sister taxa to the laccate *G. leucocontextum* from China. *G. lucidum* has a high degree of morphological variability, often resulting in taxonomic and phylogenetic confusion [38]. However, our Chinese and Thai *G. lucidum* specimens displayed a degree of morphological variability. The Chinese *G. lucidum* possessed a cylindrical stipe, and when viewed from above, one can observe the presence of a central stipe at the center of the pileus, while the Thai *G. lucidum* presented a cylindrical and eccentric stipe. However, both strains also shared key features, with their distinctive stipitate, furrowed, incised, undulate to sulcate, undefined imbricate, yellowish-red, brownish-red to reddish-orange with laccate upper pileus surface, light brown to dark brown context, and presence of dark brown melanoid bands. Our results are also in agreement with those of Wang et al. [5], who reported that Chinese *G*. *lucidum* has a deeper-colored context that is even darker near the tube layer. Although both *G. lucidum* and *G. leucocontextum* form a sister clade, their morphological characteristics are different. *Ganoderma leucocontextum* shows its distinctive white context, thickset stipe, and broadly ellipsoid basidiospores (9.5–12.5 × 7–9 μm). For descriptive details, please see Li et al. [62].

*Ganoderma tsugae* is one of the *Ganoderma* species that share similar characteristics with *G. lucidum* [4]. In this study, we collected *G. tsugae* from Yunnan Province, China. In the phylogenetic analysis, it clustered with *G. tsugae* from the USA [76] and grouped as a sister to *G. oregonense* [76,175]. However, *G. tsugae* has smaller basidiospores (9–11 × 6–8 μm) [176] than *G. oregonense* (13–17 × 8–10 μm) [109].

In this study, we introduce a new record of *G. subresinosum* collected in Thailand during the rainy season. Phylogenetic analysis indicated our *G. subresinosum* to be a distinct clade with suitable support. Macro- and micro-characteristics of *G. subresinosum* are similar to Hapuarachchi et al. [30], who demonstrated that this fungal species has a distinctively annual, sessile, laccate, dark brown upper pileus surface, concentrically sulcate, irregularly grayish-orange when dried and woody when old, presenting ellipsoid to elongate basidiospores.

Based on comprehensive characteristics and molecular analyses, we report 23 Ganoderma species from GMS, including G. adspersum, G. applanatum, G. australe, G. calidophilum, G. ellipsoideum, G. flexipes, G. gibbosum, G. heohnelianum, G. hochiminhense, G. leucocontextum, G. lucidum, G. multiplicatum, G. multipileum, G. myanmarense, G. orbiforme, G. philippii, G. resinaceum, G. sichuanense, G. sinense, G. subresinosum, G. williamsianum, G. tropicum, and G. tsugae, of which 13 Ganoderma species were collected from Yunnan Province, China, 3 species from Laos, three species from Myanmar, and 12 species from Thailand, including G. hochiminhense from Vietnam and G. myanmarense from Myanmar. We noted a high degree of intraspecies morphological variability between specimens collected from different parts of this region, confirming why it is hard to correctly identify Ganoderma to the species level using only morphological observations and why phylogenetic analyses are crucial in maintaining correct taxonomic placement within this group.

## Figures and Tables

**Figure 28:**
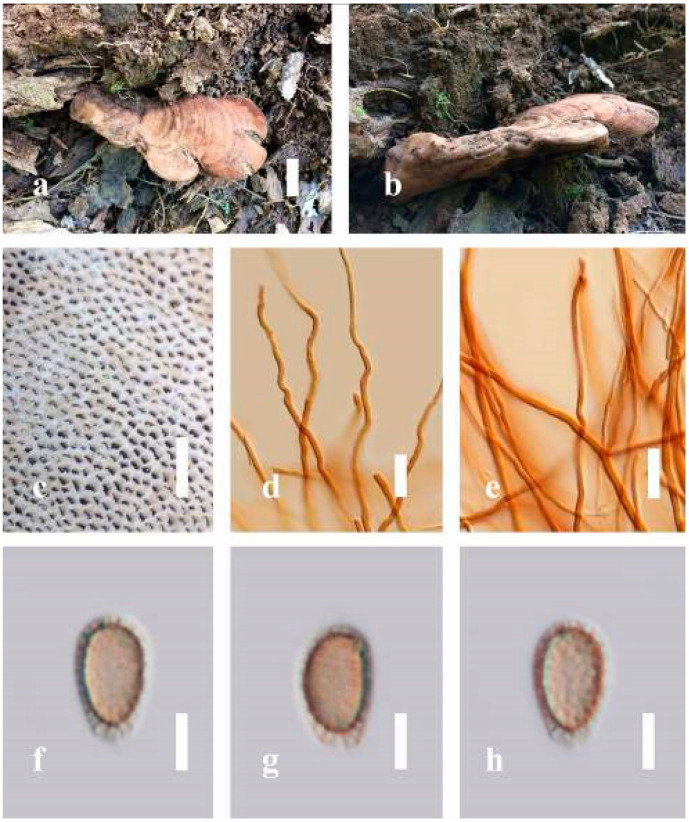
Morphology of *Ganoderma orbiforme* (MFLU 17-1933): (**a**,**b**) mature basidiomes; (**c**) pore characteristics; (**d**,**e**) context hyphae in KOH; (**f**–**h**) basidiospores in KOH. Scale bars: (**a**) = 2 cm, (**c**) = 1000 µm, (**d**,**e**) = 20 µm, (**f**–**h**) = 5 µm.

**Table 1:** Species of *Ganoderma*, host plants, diseases, and main regions of distribution within the Greater Mekong Subregion.

*Ganoderma* Species	Host Plant	Diseases	Region	Reference
*G.* *adspersum*	*Pterocarpus* sp.	Wood decay	*Thailand*	This study
	*Mangifera indica*	Wood decay	*Laos*	This study
*G.* *applanatum*	*Acer* sp.		Vietnam	[57]
	*Rhizophora apiculata*, *Machilus yunnanensis*, and hardwoods	Wood decay, Butt rot	Thailand, China, Myanmar, Laos	[30,58], this study
*G. applanatum*	*Machilus yunnanensis*	Wood decay	Yunnan, China	This study
	*Artocarpus* sp., and *Dipterocarpus* sp.	Wood decay	Thailand	This study
*G.* *australe*	*Anisoptera costata* and *Shorea robtusa*	*Wood decay*, rotten wood	Laos, Thailand, and Myanmar	[30,59], this study
	*Camellia sinensis* *var.* *assamica*	*Root rot*	*Thailand*	[60]
*G.* *calidophilum*	*Castanopsis* sp. and *Machilus yunnanensis*	*Wood decay*	*Yunnan, China*	This study
*G.* *casuarinicola*	*Pinus kesiya*	*White rot*	*Thailand*	[31], this study
*G.* *ellipsoideum*	Unknown	Wood decay	Thailand	This study
*G.* *flexipes*	*Pinus* sp. and unknown		China, Myanmar, Vietnam	[6,30,61], this study
*G*. *gibbosum*	*Albizia mollis*, *Machilus yunnanensis*, and *Pinus* sp.		Yunnan, China	This study
	*Albizia lebbeck*, *Dendrocalamus strictus*, *Dipterocarpus* sp., *Mangifera indica*, *P. pterocarpum*, and *Pinus* sp.	Wood decay, rotten wood	Thailand	This study
	Hardwoods	Wood decay	China, Thailand	[30], this study
*G.* *hochimin-hensis*	*Areca* sp.	Root rot	Vietnam	This study
*G.* *hoehnelianum*	Unknown	Wood decay	Myanmar	This study
*G.* *leucocontextum*	*Cyclobalanopsis glauca* and *C. glauca*	Wood decay, root rot	Yunnan, China	[62], this study
*G.* *lucidum*	*Quercus* sp.	Wood decay	*Yunnan, China*	This study
	*Acacia* sp., *Dendrocalamus strictus*, and *Pterocarpus* sp.	Wood decay	*Thailand*	This study
*G.* *multipileum*	Unknown decayed hardwood	Wood decay	*Yunnan*, China	[30], this study
	*Pinus merkusii*	Wood decay	Thailand	This study
*G.* *multiplicatum*	*Quercus* sp.	*Wood decay*	*Yunnan, China*	This study
*G.* *myanmarense*	Unknown tree	*Wood decay*	*Myanmar*	This study
*G.* *nasalaense*	Not mention	*Wood decay*	Laos	[36]
*G.* *neojaponicum*	Near hardwood roots	Wood decay	Myanmar	[30]
*G.* *orbiforme*	*Albizia mollis* and *Indochinese* sp.	Wood decay	*Thailand*	This study
	Unknown decayed hardwood	Wood decay	China, Laos	This study
	*Elaeis guineensis*	Basal stem rot	Thailand	[63]
*G.* *philippii*	Unknown	Root rot	Thailand	This study
*G.* *resinaceum*	*Albizia mollis*	*Basal stem rot*	*Yunnan, China*	This study
*G. sichuanense*	*Quercus* sp.		*China*	[4,40]
	*Pterocarpus* sp., *Mangifera indica*, *Sesbania grandiflora*, and *Peltophorum pterocarpum*	Wood decay	Thailand	This study
	*Castanopsis* sp., *Castanea* sp., *Cyclobalanopsis* sp, and *Graucoides schotky*	Wood decay, root rot	Yunnan, China	[4], this study
*G.* *sinense*	*Albizia mollis* and *Quercus* sp.	Rotten wood	Yunnan, China	[30], this study
	*Dendrocalamus strictus* and *Dipterocarpus* sp.	*Wood decay*, Rotten wood	Thailand	This study
*G.* *subresinosum*	*Peltophorum pterocarpum* and *Castanopsis* sp.	Wood decay	Thailand	This study
*G.* *thailandicum*	*Pinus merkusii*	Wood decay	Thailand	[31], this study
*G.* *tropicum*	*Dipterocarpus* sp.	Wood decay	Thailand	[31], this study
*G.* *tsugae*	*Larix* sp., *Picea* sp. and *Tsuga* sp.	*Butt rot*, Wood decay	*China*	[57,64], this study
*G*. *williamsianum*	Unknown	Wood decay	Myanmar	This study

**Table 2:** Regional information for collection sites in the Greater Mekong Subregion, where collections occurred.

Study Sites	China	Myanmar	Laos	Thailand	Vietnam
Climate Type	Temperate	Tropical	Tropical	Tropical	Tropical
Collecting date	December 2016; September– December 2017; September– November 2018	July 2019	June–July 2018	October–December 2017; June and November 2018	June 2019
Monthly temperature	16–22 °C	26–30 °C	25–32 °C	25–38 °C	28–32 °C
Forest type	Coniferous forest, dry evergreen forest, and evergreen coniferous forest	Mixed deciduous forest and tropical rain forest.	Deciduous forest and tropical rain forest	Coniferous forest, deciduous forest, dry evergreen forest, and tropical rain forest.	Deciduous forest
Host tree species	*Albizia mollis*, *Castanopsis* spp., *Fagus* spp., *Machilus yunnanensis*, *Pinus* spp., *Pterocarpus macrocarpus*, *Quercus* spp., and *Hevea brasiliensis*	Unidentified tree species	*Mangifera indica*	*Acacia* sp., *Albizia lebbeck*, *Artocarpus* spp., *Castanopsis* spp., *Dendrocalamus strictus*, *Dipterocarpus* spp., *Garuga pinnata*, *Indochinese* spp., *Maerus siamensis*, *Mangifera indica*, *Pterocarpus macrocarpus*, *Peltophorum pterocarpum*, and *Pinus* spp.	*Areca* spp.

## Supplementary Materials

The following are available online at https://www.mdpi.com/article/10.3390/jof7100819/s1, Figure S1: Collection sites of specimens used in this study in China, Laos, Myan-mar, Thailand, and Vietnam. Black triangles indicate collection locations for the specimens; Table S1: The extant *Ganoderma* species in the GMS.
