# Supplementary material for: Ganoderma (Ganodermataceae, Basidiomycota) Species from the Greater Mekong Subregion
Source: J Fungi (Basel). 2021 Sep 30;7(10):819. doi: 10.3390/jof7100819 (PMC8541142; doi:10.3390/jof7100819)
Supplement: Supplementary file 1 [file jof-07-00819-s001.zip › jof-1339161-Supplementary.pdf]

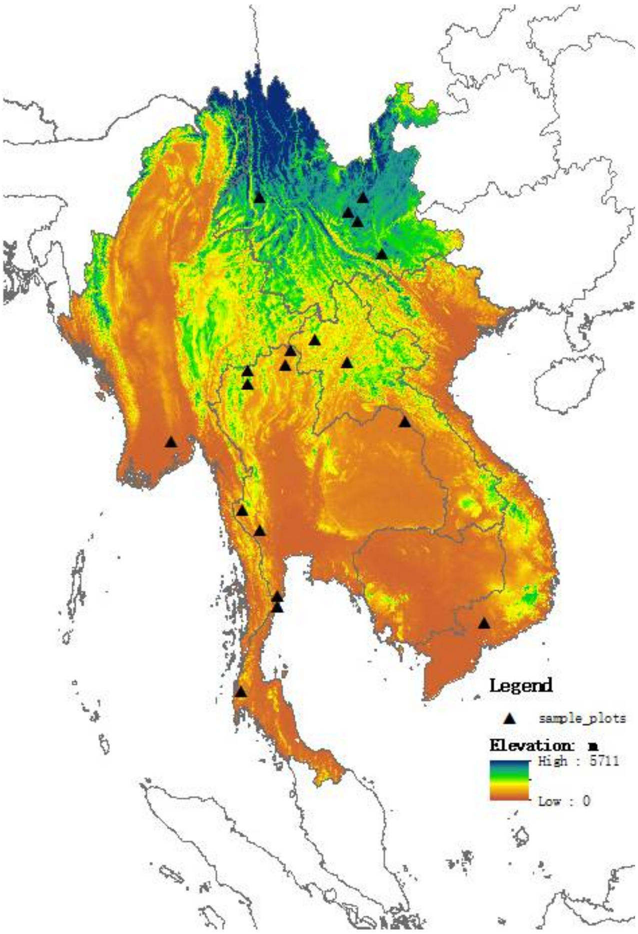

Figure S1. Collection sites of specimens used in this study in China, Laos, Myanmar, Thailand, and Vietnam. Black triangles indicate collection locations of the specimens.

Table S1. The extant *Ganoderma* species in the GMS.

Countries	*Ganoderma* species
Laos	*Ganoderma adspersum, G. australe, G. gibbosum, G. flexipes, G. luteomarginatum, G. lucidum, G. neojaponicum, G. nasalanense, G. subresinosum, G. tropicum*
Myanmar	*G. amboinense, G. applanatum, G. australe, G. gibbosum, G. hoehnelianum, G. multiplicatum, G. myanmarense, G. neojaponicum, G. williamsianum*
Thailand	*G. adspersum, G. applanatum, G. australe, G. boninense, G. casuarinicola, G. donkii, G. ellipsoideum, G. gibbosum, G. lucidum, G. multipileum, G. orbiforme, G. philippii, G. sichuanense, G. sinense, G. subfornicatum, G. thailandicum, G. tropicum*
Vietnam	*G. applanatum, G. flexipes, G. gibbosum, G. hochiminhense, G. philippii*
Yunnan Province, China	*G. applanatum, G. australe, G. calidophilum, G. flexipes, G. gibbosum, G. leucocontextum, G. lucidum, G. multiplicatum, G. resinaceum, G. sichuanense, G. sinense, G. tropicum, G. tsugae, G. weixiensis*
